# SPLICEFINDER – A Fast and Easy Screening Method for Active Protein Trans-Splicing Positions

**DOI:** 10.1371/journal.pone.0072925

**Published:** 2013-09-02

**Authors:** Joachim Zettler, Simone Eppmann, Alena Busche, Dina Dikovskaya, Volker Dötsch, Henning D. Mootz, Tim Sonntag

**Affiliations:** 1 Department of Chemistry and Chemical Biology, TU Dortmund University, Dortmund, Germany; 2 Institute of Biophysical Chemistry and Center for Biomolecular Magnetic Resonance, Goethe University, Frankfurt/Main, Germany; 3 CRUK Beatson Laboratories, University of Glasgow, Glasgow, United Kingdom; International Centre for Genetic Engineering and Biotechnology, Italy

## Abstract

Split intein enabled protein *trans*-splicing (PTS) is a powerful method for the ligation of two protein fragments, thereby paving the way for various protein modification or protein function control applications. PTS activity is strongly influenced by the amino acids directly flanking the splice junctions. However, to date no reliable prediction can be made whether or not a split intein is active in a particular foreign extein context. Here we describe SPLICEFINDER, a PCR-based method, allowing fast and easy screening for active split intein insertions in any target protein. Furthermore we demonstrate the applicability of SPLICEFINDER for segmental isotopic labeling as well as for the generation of multi-domain and enzymatically active proteins.

## Introduction

**Figure 1:**
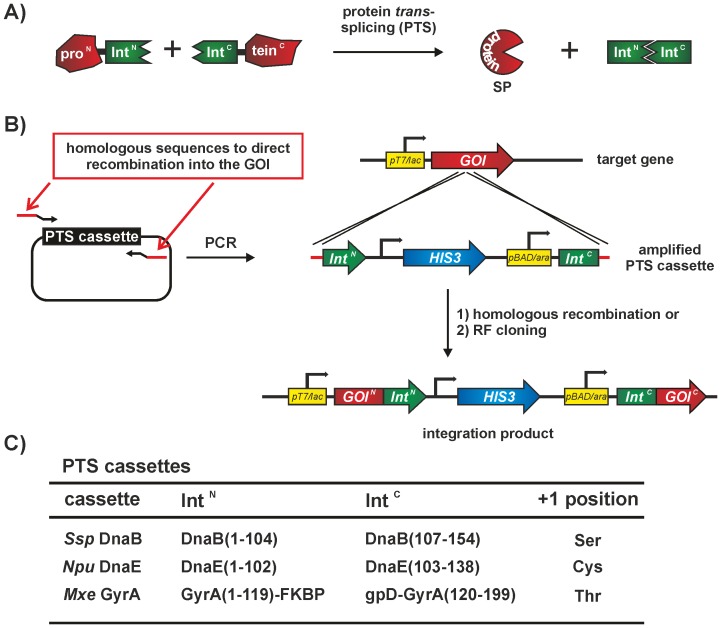
Principle of PTS and SPLICEFINDER. (A) The PTS reaction scheme. B) Schematic representation of the PTS cassette amplification and insertion procedure. The point of integration into the target gene (gene of interest; *GOI*) is controlled by the sequence of the PCR primers used for PTS cassette amplification. The integration of the PTS cassettes can be achieved in two ways: approach **1)** uses homologous recombination in *S. cerevisiae* and approach **2)** is based upon restriction-free (RF) cloning. Notably, in both approaches the amino acids flanking the intein can be adapted by the primer sequence. C) The PTS cassettes constructed and used in this study. (SP = splice product).

In recent years protein *trans*-splicing (PTS) [1], [2] has become an important tool for both the chemical modification of proteins [3], [4] and for the control of protein function [5], [6]. PTS relies on the capability of two split intein fragments to efficiently link their flanking sequences, known as the exteins, through a native peptide bond (Figure 1A). The influence of the extein substrates on the success of the ligation reaction remains poorly understood. In addition to the C-terminal amino acid at the splice junction (+1 position; can be either Cys, Ser or Thr), which is directly involved in the protein splicing mechanism and is the only invariant splice product remnant, the extein amino acids flanking the splice junction (−2, −1, +2 and +3 positions) also contribute to the ligation efficiency [7]–[13]. Several efforts have been made to catalogue these extein dependencies for the *Npu* and *Ssp* DnaE split inteins [8], [9] and the influence of the *Npu* DnaE intein +2 position was explained in detail on the structural level [14]. Furthermore, a FRET based assay for the fused *Ssp* DnaE intein [7] and a genetic screen for the fused *Npu* DnaE intein [9] were performed, both studying the impact of the amino acids immediately flanking the intein. In addition, structural studies of the *Pyrococcus horikoshii* RadA mini *cis*-intein allowed the engineering of a more promiscuous intein towards the N-terminal extein junction [15] and directed evolution approaches were able to directly change or improve the extein tolerance of the *Ssp* DnaE and *Npu* DnaE inteins [10], [16]. Taken together, these studies strengthen the hypothesis that protein splicing is strongly extein-dependent. While the use of modelling approaches or computer based programs can assist in choosing possible split sites in proteins [17], [18], increased success rate relies upon the existence of a 3D structure of the desired protein, which is often not available.

Despite all the efforts mentioned above, at the moment no reliable *a priori* prediction can be made about whether an intein will be active in a particular non-native extein context or estimations about the splicing efficiency. One approach to retain or increase the activity of a split intein is to adjust the splice junction amino acid composition towards the natural extein sequence. To bypass the tedious cloning steps required for generating and testing multiple insertion positions, our group has recently developed a split intein cassette based integration approach, utilizing homologous recombination in yeast [6]. This conditional split intein system depends on the addition of the small molecule rapamycin and was successfully applied to control the function of the tobacco etch virus (TEV) protease.

In the work presented here, we have developed SPLICEFINDER, which extends the above method to non-conditional split inteins, which are often used for protein modification. SPLICEFINDER can be used to identify active split intein insertion positions in any desired target protein. However, it is specifically designed to facilitate the production of segmental isotopically labelled proteins for NMR investigations via *in vivo* PTS. Fully labelled proteins may suffer from signal overlap due to a high number of signals or poorly dispersed spectra. Reduction of NMR signals can be achieved by incorporating NMR active isotopes only specific protein parts, therefore enabling the capture of NMR spectra with reduced signal overlap [4], [19], [20]. To date a large number of publications report successful NMR investigations on segmental isotopically labelled large or multi-domain proteins. These proteins are either produced via PTS or through other ligation methods, such as expressed protein ligation (EPL) [21]–[25]. Even the labelling of central protein domains is possible with the help of orthogonal intein pairs (in the approach of PTS) or with kinetically controlled ligation reactions and protected N-terminal cysteine residues (in the approach of EPL) [26]–[29]. The production of segmental isotopically labelled proteins via *in vivo* PTS is especially attractive, because it eliminates the need for the purification of the individual precursor intein fusion proteins. This can be achieved through selective expression of the two corresponding intein fusion proteins in *E. coli* cells in media containing different isotopes [30]–[32].

Here, we present the advantage and feasibility of the SPLICEFINDER method for the *in vitro* as well as *in vivo* production of segmental isotopically labelled proteins for NMR spectroscopy and demonstrate the successful incorporation of split inteins in a complex multi-domain protein as well as in a catalytically active enzyme.

## Materials and Methods

All Materials and Methods as well as an extended experimental procedure section can be found in the Supplementary Information (File S1).

## Results and Discussion

### General Concept

A successful PTS reaction between an N- and C-terminal intein fusion protein leads to the assembly of a ligated splice product (see Figure 1A). In two steps, the SPLICEFINDER system generates a bi-inducible plasmid, comprising both intein fusion genes (see Figure 1B). For our dual induction system we decided to use the well described IPTG/Arabinose expression systems [30], [32], [33] and generated three PTS cassettes based on the naturally split *Npu* DnaE [8], [34], the artificially split *Ssp* DnaB [35], [36] and *Mxe* GyrA inteins [37] (see Figure 1C – a detailed description of the Mxe GyrA intein cassette can be found in the SI and Figure S4). All split inteins have different nucleophiles at the +1 position and are active at physiological conditions, such that no denaturing and refolding steps are necessary for splicing activity.

In detail, the PTS cassette plasmids consist of the N-terminal intein region, a T7 terminator, the HIS3 marker gene and the C-terminal intein region under the control of the pBAD/ara promoter/operator (see Figure S1 for sequences). The only requirement for the target vector is a coding gene of interest (GOI) under the control of the T7/lac promoter/operator (ideally fused to 5′ and 3′affinity tags). After integration into the selected position, the result is a bi-inducible plasmid, with the N-terminal fusion protein being under the control of IPTG inducible expression, and the C-terminal fusion protein being controlled by arabinose. To genetically integrate the intein cassettes we utilized two different approaches (**1)** and **2)** in Figure 1B, and see SI for extended protocols), homologous recombination in *S. cerevisiae* [38] and restriction-free cloning [39]. The common step is the PCR amplification of the PTS intein cassette from the template. In this reaction primers add 40 bp of homologous sequence to the gene of interest on each side of the intein cassette, enabling the site-specific genetic integration via approaches **1)** and **2)**. The advantage of our system is that different insertion positions with variable amino acid codons at the splice junctions can be generated in parallel, by simply modulating the primer sequence. Since the integration process is site-specific and traceless (without the addition of extra nucleotides in the target gene) the resulting PTS cassettes are applicable to a wide range of available T7 expression system. As such this process eliminates the need to clone the intein gene fragments individually. After co-transformation with a helper plasmid (see SI for construction details) that codes for the regulatory proteins, LacI and AraC, small-scale *E. coli* test expressions and subsequent Western blot analysis will determine whether a certain insertion position is splice active.

### Proof of Concept – Segmental Isotopic Labelling

**Figure 2:**
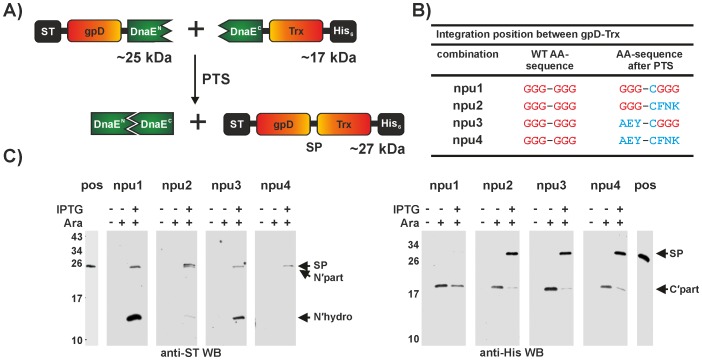
PTS reaction to generate ST-gpD-Trx-His_6_. A) Schematic representation of the *Npu* DnaE intein PTS reaction following the integration into the example protein ST-gpD-Trx-His_6_. B) Amino acid sequences at the splice junction in the linker region of ST-gpD-Trx-His_6_. Linker amino acids that differ from the original sequence (WT AA-sequence) are shown in blue. C) Western blot analysis of the *in vivo* PTS reaction to assemble ST-gpD-Trx-His_6_. The theoretical molecular masses of the proteins are as follows: Splice product (**SP**) = 26.5–26.9 kDa; N-terminal precursor protein (**N’part**) = 25.0–25.2 kDa; C-terminal precursor protein (**C′part**) = 17.4–17.6 kDa; N-terminal hydrolysis product – ST-gpD (**N′hydro**) = 13.2–13.4 kDa. (pos = full length ST-gpD-Trx-His_6_)_._

The first model protein used to test SPLICEFINDER consisted of two stably folded protein domains, the bacteriophage lambda head protein D (gpD) and thioredoxin, linked through six glycine residues. We integrated both the *Npu* DnaE (see Figure 2 and Table S2) as well as the *Ssp* DnaB intein cassette (see Figure S5 and Table S1) into the linker region. For both inteins we generated four different combinations of flanking amino acids at the splice junction. Although both integration approaches were successful, the restriction-free cloning procedure (approach **2)**) is preferred, because it is more efficient and requires only standard molecular biology techniques without the need for yeast cultivation (see SI for protocol and Table S5 for statistical evaluation). In one of our generated integration variants (npu1), the splice product only contains the insertion of the +1 nucleophile (GGG**C**GGG for the DnaE). In the other three versions the flanking amino acids are exchanged to the wild type extein sequence (AEY**C**FNK) of the intein, either at the N- or C-terminal junction or at both (see Figure 2B). No splice product was detected in the case of the *Npu* DnaE with three glycines at both sites (npu1). Furthermore, the presence of the N-terminal hydrolysis product in the anti-Strep-Tag (ST) Western blot indicated that the complex formation as well as the first N-S acyl occurred (see Figure 2C). However, the splicing pathway was blocked in subsequent reaction steps with these “unnatural” extein substrates. Adjusting the N-terminal splice junction (combination AEY**C**GGG, npu3) shows N-terminal hydrolysis as well as generation of the splice product. Recently another study described a certain level of tolerated sequence variability for the *Npu* DnaE intein on both the N- and C-terminal splice junction [9]. Moreover a bulky side chain at the +2 extein position seems to be an important factor in the rate determining step of the splice reaction [14]. Our result, a splice active glycine at +2 (npu3), is in contrast to the previous findings and therefore support our presumption that it is difficult to predict the activity of an intein in a foreign extein context and, currently, only an experimental study can fully answer this question. Both combinations with adjusted C-terminal splice junctions (npu2 and npu4) were splice active and no hydrolysis by-products could be detected (Figure 2C). To confirm the observed discrimination between splice active and inactive combinations via small-scale expression and Western blot analysis, we recloned the intein fusion genes, expressed and purified the individual proteins and conducted *in vitro* splice assays. The results confirmed our initial observations in all four cases (data not shown), indicating that Western blot analysis is a sufficient method for the determination of PTS activity.

**Figure 3:**
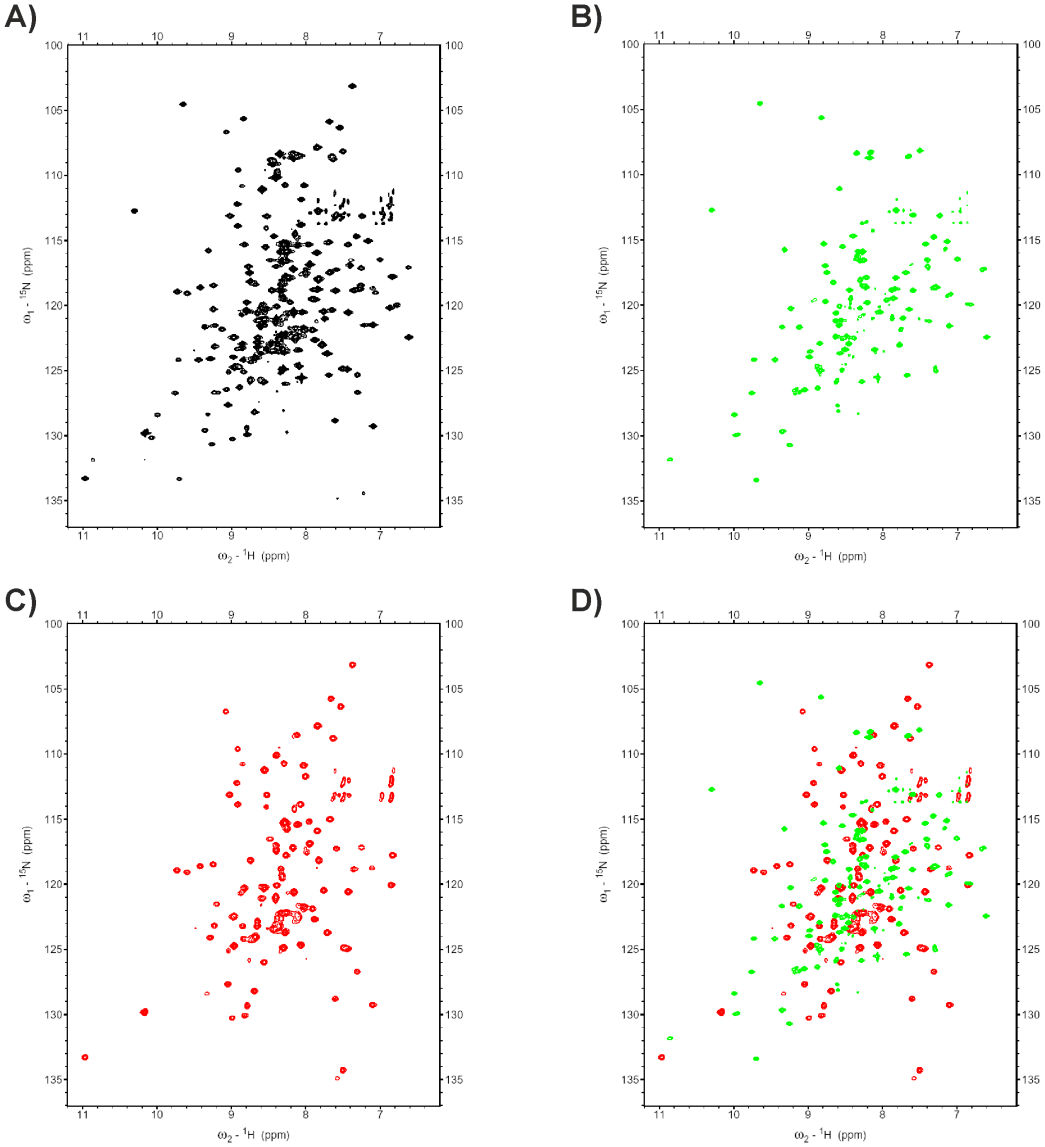
Segmental labelling of the model protein ST-gpD-Trx-His_6_. ^15^N-^1^H HSQC NMR spectra (900 and 700 MHz at 293K) of A) the uniformly ^15^N-labelled ST-gpD-Trx-His_6_ (black), B) C-terminally ^15^N-labelled ST-gpD-[^15^N]-Trx-[^15^N]-His_6_ (produced via *in vitro* splicing with the *Npu* DnaE intein) (green), and C) N-terminally ^15^N-labelled [^15^N]-ST-[^15^N]-gpD-Trx-His_6_ (produced via *in vivo* splicing with the *Ssp* DnaB intein) (red). D) Overlay of B) and C).

To demonstrate that the SPLICEFINDER system can be used to obtain segmental isotopically labelled proteins for NMR studies, we produced an N- and a C-terminal ^15^N-segmental isotopically labelled gpD-Trx fusion protein via *in vivo* and *in vitro* PTS on a larger scale and analysed it via NMR spectroscopy. The labeling of the C-terminal Trx-His6 fragment was achieved by *in vitro* PTS using the purified *Npu* DnaE intein proteins (see Figures S6 and S7). The isotopic enrichment of the N-terminal ST-gpD fragment was done via *in vivo* PTS with the *Ssp* DnaB intein (for details see SI and Figure S8). The comparison of their ^15^N-^1^H HSQC spectra with that of a completely ^15^N-labelled model protein confirmed that the segmental isotopic labelling was successful (Figure 3).

The advantage of an *in vivo* production of the splice product compared with an *in vitro* assembly of the two protein parts is the reduced number of purification steps. However, *in vivo* segmental labelling of proteins via PTS requires highly selective expression of the protein precursors in the special growth media to avoid “scrambling effects” of the isotopes. To analyse the efficiency of the isotopic labelling during different expression conditions, we employed small-scale expressions in ^15^N-labelled media, followed by tryptic digest of the SDS-PAGE band corresponding to the splice product, and subsequently MALDI-TOF MS analysis (for a detailed analysis and discussion see the SI, Figure S2 & S3). This procedure allows for rapid determination of the optimal expression condition, without the need for large scale splice product purification and consecutive NMR spectra recording.

### Application to Large Multi-domain Proteins

Based on the successful application to our model protein and the use for *in vitro* or *in vivo* segmental isotopic labelling, we wanted to expand SPLICEFINDER to more complex target proteins. Therefore we next choose a non-ribosomal peptide synthetase (NRPS) module. Non-ribosomal peptide synthetases (NRPS) are large, multi-domain proteins that produce a variety of secondary metabolites in bacteria and fungi [40]–[42]. For SPLICEFINDER, we chose the second module of the Gramicidin S biosynthesis pathway (see Figure S9), which included the first three domains of Gramicidin S synthetase II (GrsB1). With an additional N-terminal ST and a C-terminal His_6_-Tag, the protein consisted of 1071 amino acids corresponding to a molecular weight of 124 kDa.

**Figure 4:**
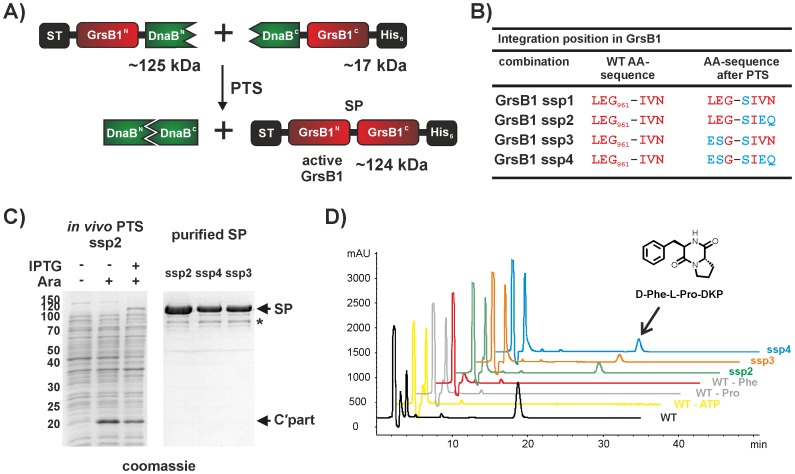
Integration of the *Ssp* DnaB intein cassette into the NRPS module Gramicidin S Synthetase B1. A) Schematic representation of the PTS reaction following the integration of the *Ssp* DnaB intein into GrsB1. B) Amino acid sequences at the splice junction at position 961. Deviations after splicing from the original sequence (WT AA-sequence) are shown in blue. C) (left) − SDS-PAGE analysis of the individually and dual induced combination GrsB1 ssp2; (right) SDS-PAGE analysis of purified splice products (**SP**) (combinations GrsB1 ssp2–4). The theoretical molecular masses of the proteins are as follows: N-terminal precursor protein (**N’part**)** = **124.9 kDa; Splice product (**SP**) = 124.2 kDa; C-terminal precursor protein (**C’part**) = 16.7 kDa. Impurities (*) are indicated. D) Activity assay for the GrsB1 splice products assembled through *in vivo* PTS with the *Ssp* DnaB intein. Analytical HPLC chromatograms (absorbance at 210 nm) are shown. The trace for the positive control, the full length wild type (WT) GrsB1 is shown in black. The peak at around 18.5 min was assigned via MS to the cyclic dipeptide D-Phe-L-Pro-diketopiperazine (DKP). When one of the substrates is omitted (ATP - yellow trace; Pro - grey trace; Phe - red trace) no product formation is detected. All purified GrsB1 splice products show DKP formation (GrsB1 ssp2–4).

We chose the linker region between the adenylation (A) domain and the PCP domain for the position of *Ssp* DnaB intein cassette insertion (Figure 4) and tested four different variations of flanking amino acids (see Figure 4B and Table S3). The four different combinations were analysed for their splice activity in small-scale expressions (see Figure S10). Out of these four cases, only in one the splice product was not detectable (GrsB1 ssp1) due to an insertion of a single serine residue in the theoretical splice product without any natively flanking amino acids of the intein. The presence of bands corresponding to the C-terminal half in the anti-His and in the N-terminal half in the anti-ST Western, indicated that this combination is indeed not splice active (Figure S10). Interestingly, adjusting two amino acids at the +3 and +4 positions to the native extein residues of the DnaB intein restored intein activity (GrsB1 ssp2). Similar results were observed for the −3 and −2 positions in the N-terminal part (GrsB1 ssp3) as well as for the double adjustment combination (GrsB1 ssp4).

The formation of the GrsB1 splice products on a larger scale was achieved via *in vivo* PTS with consecutive protein induction (see Figure 4C and SI for a detailed description). The splice products were purified via Ni^2+^-NTA affinity chromatography and additional gel filtration. To test whether the GrsB1 splice products were still able to perform nonribosomal peptide assembly we used the previously described assay for D-Phe-L-Pro-diketopiperazine (DKP) formation (see Figure S11) [43], [44]. Briefly, the incubation of the first two modules of the Gramicidin S biosynthesis pathway together with the appropriate substrates yields the D-Phe-Pro dipeptide tethered as thioester onto the PCP domain of GrsB1. Spontaneous, uncatalysed cyclization results in the release of D-Phe-L-Pro-DKP, which can be detected by HPLC analysis.

All three GrsB1 proteins obtained through PTS with the *Ssp* DnaB intein were able to catalyse the formation of DKP (see Figure 4D). Additionally, we confirmed that a recombinantly generated serine insertion after G_961_ in the linker region between the A and the PCP domain of GrsB1, resulting in the sequence of combination GrsB1 ssp1, yielded an active protein in the DKP assay (data not shown). This suggests that the linker region between the A- and the PCP-domains in NRPS tolerates amino acid insertions and substitutions.

Taken together, we were able to show that the SPLICEFINDER technology is also applicable to large multi-domain proteins with molecular weights larger than 100 kDa. The obtained splice products were still enzymatically active, enabling intein mediated site specific incorporation of biophysical probes, like fluorophores [37].

### Integration into a Folded Domain of a Catalytically Active Protein

To further explore the application range of the SPLICEFINDER method, we integrated one of the PTS cassettes directly into the functional domain of an enzyme. In this case the enzymatic activity should only be restored upon successful protein splicing. We choose the uroporphyrinogen III methyltransferase (CobA) of *Propionibacterium freudenreichii* [45], [46]. This class of enzymes is involved in the tetrapyrrole biosynthesis in diverse organisms catalysing the conversion of uro(porphyrino)gen III to precorrin-2 [45]–[47]. S-Adenosylmethionin (SAM) acts as the methyl group donor for methylation reactions at the tetrapyrrol ring (see Figure S12A for the reaction scheme). Overproduction of the CobA enzyme leads to the accumulation of red fluorescent compounds, either because accumulated precorrin-2 is oxidized to sirohydrochlorin or CobA further methylates precorrin-2 to trimethylpyrocorphin or to tetramethylated compounds [46].

**Figure 5:**
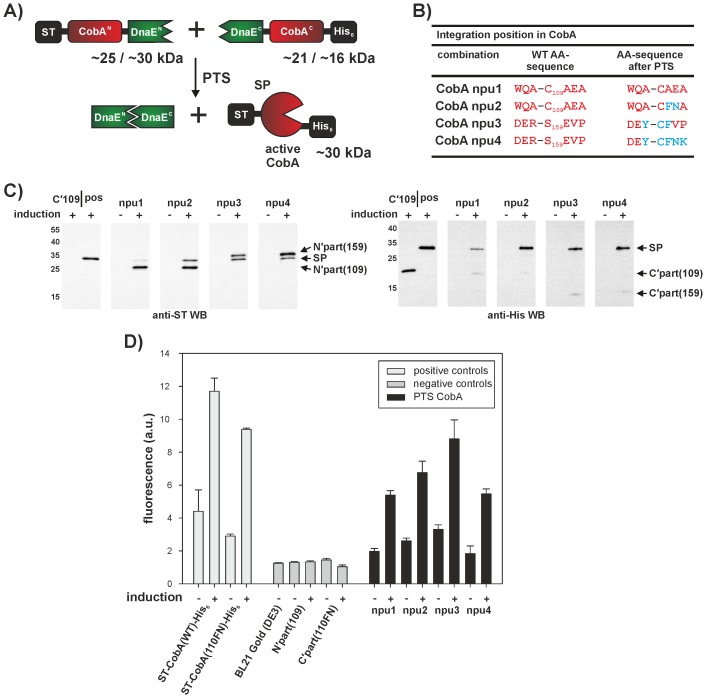
Integration of the *Npu* DnaE intein cassette into the uroporphyrinogen III methyltransferase CobA. A) Schematic representation of the PTS reaction after integration of the *Npu* DnaE PTS cassette into *cobA*. B) Amino acid sequences at the splice junctions of position 109 and at position 159. Amino acids differing from the original sequence (WT AA-sequence) are shown in blue. C) Western blot analysis of the *in vivo* PTS to assemble ST-CobA-His_6_. Expressions of the complete proteins, as well as of the individual halves and the co-induced integration plasmids were done on a small-scale at 20°C for 48 h. The theoretical molecular masses of the proteins are as follows: **ST-CobA-His_6_** = 29.5 kDa; **N’part(109)** = 24.6 kDa; **C’part(109)** = 20.7 kDa; **N’part(159)** = 29.7 kDa; **C’part(159)** = 15.7 kDa. (pos = full length ST-CobA-His_6_)_._ D) Activity test of ST-CobA-His_6_ splice products. Fluorescence intensity of the induced (+) and non-induced (-) samples after 48 h (Exc. 357 nm, Em. 605 nm, see SI for details). As one of the negative controls, the value for *E. coli* BL21 Gold (DE3) cells without any plasmid is shown. All measurements were performed at least in duplicate.

Because of these properties, CobA was used as a red fluorescent transcriptional reporter in *E. coli*, yeast, and mammalian cells [48], and also as a whole-cell sensing system in *E. coli* [49]. Although CobA does not require the addition of exogenous substrates, the addition of δ-aminolevulinic acid (ALA), a precursor of the tetrapyrrol biosynthesis, resulted in a more stable and reproducible readout [49]. We decided to integrate the *Npu* DnaE PTS cassette into the *cobA* gene (see Figure 5A for PTS reaction scheme), because this intein is superior to the other two in terms of the reaction velocity [34]. The *Npu* DnaE possesses a cysteine residue at the +1 position, so we choose the only native cysteine at position 109 as an integration position (see Figure S12B). Alternatively, we mutated a serine at position 159, an unconserved region of the protein, into a cysteine (detailed description in the SI, see Figure S12B and C).

We conducted the integration of the split intein cassette at both positions and produced two different combinations of flanking amino acids at the C-terminal splice junction, respectively (see Figure 5B and Table S4). The PTS reaction for the split CobA at residue 109 would result either in a ST-CobA-His_6_ protein with the native amino acid composition (CobA npu1) or a version with residues +2 and +3 adjusted to the native *Npu* DnaE intein extein residues (CobA npu2). After the PTS reaction, one combination at position 159 should yield a CobA variant with 3 amino acid substitutions (CobA npu3), while the other additionally adjusts the +3 and +4 residues (CobA npu4).

The small-scale expression experiments showed the formation of the splice product for all combinations (see Figure 5C). However, for the combination with the wild type CobA sequence at the splice junction (CobA npu1), the amount of splice product was reduced relative to the other combinations. This observation is consistent with previous results, which indicated, that the canonical CFN tripeptide at the C-terminal splice junction of the DnaE inteins is not a strict prerequisite for splicing activity, but adjusting the +2 and +3 positions to the native extein residues, can enhance the PTS activity.

Subsequently, we utilized the formation of red fluorescent compounds as a read-out for CobA activity. Because of insolubility issues (see SI and Figure S13) the expression was performed for 48 hours at 20°C and samples of the small-scale expressions were used to determine the fluorescence intensity of the *E. coli* cells (see Figure 5D, and SI for experimental details).

The intensity measurements indicated that all PTS assembled CobA proteins were enzymatically active. CobA npu1 and CobA npu4 reached approximately half of the intensity produced by the wild-type ST-CobA-His_6_. The CobA npu2 and CobA npu3 displayed up to two-thirds of the wild-type activity. The PTS controls (only the C-terminal and N-terminal intein fusion proteins) showed a similar background intensity as plasmid-free BL21-Gold(DE3) cells. The uninduced samples of all PTS combinations still showed significantly higher fluorescence intensity than the control BL21-Gold(DE3) cells, which might be due to leakiness of the promoters.

## Conclusion

In this report we presented SPLICEFINDER, a method facilitating the easy screening for active split intein insertions in any target protein. The steps include the PCR amplification of the intein cassettes from a donor vector, one round of integration, and an analysis of the small-scale expression. The entire procedure can efficiently be accomplished within two weeks. In the future we expect our approach to be expanded to novel split inteins, that either possess superior reaction kinetics or are highly promiscuous with regard to foreign extein sequences [16], [50].

Currently the strength of SPLICEFINDER relies upon the simplicity and the availability of the components necessary to conduct the insertion procedure. In conclusion we have created a new tool, which we expect will support the dissemination and more widespread application of split inteins, especially in the context of segmental isotopic labelling of proteins for NMR studies.

## Supporting Information

Figure S1 DNA-sequences of the intein cassettes. Sequences are shown from position 1 of Int^N^ to position +1 of Int^C^. The plasmid carrying the *Ssp* DnaB intein cassette (2386 bp) is pCasDnaB2, the plasmid of the *Npu* DnaE intein cassette (2338 bp) is pCasDnaE2, and the plasmid encoding the *Mxe* GyrA intein cassette (3329 bp) is pCasGyrA2.
(TIF)

Figure S2 Labelling efficiency determination. MALDI-TOF MS analysis of an N-terminal A) and a C-terminal B) peptide fragment after tryptic digest of the unlabelled (black) and completely (red) ^15^N-labelled model protein ST-gpD-Trx-His_6_ (AS denotes amino acid sequence).
(TIF)

Figure S3 MALDI-TOF MS analysis of two different expression conditions for segmental isotopic labelling via *in vivo* PTS of the model protein ST-gpD-Trx-His_6_. A) and B) MS spectra for condition 1 (green); C) and D) MS spectra for condition 2 (blue). All spectra are shown in comparison with a completely unlabelled (black) and a completely ^15^N-labelled (red) sample. A) and C) show an N-terminal fragment (amino acid sequence 15–41) and B) and D) show a C-terminal fragment (amino acid sequence 230–242) (for details on the expression conditions and MS analysis see text).
(TIF)

Figure S4 The Mxe GyrA intein cassette. A) Schematic representation of the Mxe GyrA intein cassette mediated splice reaction B) Western blot analysis of small-scale expression of *E. coli* cells containing the intein cassette plasmid, as well as the helper plasmid pRSFara. The single inductions were done for 4 h at 37°C (0.2% arabinose or 0.4 mM IPTG). The dual inductions: 0.2% arabinose for 2 h at 37°C, then media exchange, and subsequent induction with 0.4 mM IPTG for 4 h, 25°C. The theoretical molecular masses of the proteins are: **SP** = 44.7 kDa; **N’Part** = 69.3 kDa; **C’Part** = 22.2 kDa.
(TIF)

Figure S5 Integration of the *Ssp* DnaB intein cassette into gpD-Trx. A) Schematic representation of the PTS reaction after the integration of the *Ssp* DnaB PTS cassette into ST-gpD-Trx-His_6_. B) Amino acid sequences at the splice junctions for the produced combinations in the linker region of ST-gpD-Trx-His_6_. Amino acids deviations after splicing from the original sequence (WT AA-sequence) are shown in blue. C) Western blot analysis of the four different flanking amino acids variations at the splice junction. All four combinations are splice active. The calculated molecular weights of the proteins are as follows: **SP** = 26.5–26.8 kDa; **N’Part** = 24.9–25.0 kDa; **C’Part** = 18.8–19.0 kDa; **N’Hydro** = 13.2 kDa. (pos = full length ST-gpD-Trx-His_6_)_._
(TIF)

Figure S6 *In vitro* PTS to obtain segmental labelled ST-gpD-^15^N(Trx-His_6_) with the *Npu* DnaE intein. The SDS-PAGE gel of the PTS reaction and of the purification steps is shown in the Coomassie-staining. Lane 1: purified N-terminal part ST-gpD-Int^N^; lane 2: purified C-terminal part Int^C^-Trx-His_6_; lane 3: PTS-reaction at 0h; lane 4: PTS-reaction at 16 h; lane 5: combined elution fractions after Ni^2+^-NTA chromatography; lane 6: combined elution fractions after Strep-Tactin purification. The theoretical molecular masses of the proteins are as follows: **SP** = 26.7 kDa; **Part N** = 25.0 kDa; **Part C** = 17.6 kDa; **Cleav N** = 13.2 kDa; **Cleav C** = 13.5 kDa; **Int N** = 11.9 kDa; **Int C** = 4.1 kDa.
(TIF)

Figure S7 MALDI-TOF MS analysis of the segmental labelled gpD-^15^N(Trx) splice product. A) Analysis of an N-terminal fragment AS 15–41, B) Analysis of a C-terminal fragment AS 230–242. Spectra of the unlabelled (black) and complete ^15^N labelled references (red) are shown in comparison with the segmental isotopically labelled gpD-^15^N(Trx) splice product (green) obtained through *in vitro* splicing with the *Npu* DnaE intein.
(TIF)

Figure S8 MALDI-TOF MS analysis of the segmental labelled ^15^N(gpD)-Trx splice product. A) Analysis of an N-terminal fragment AS 15–41, B) Analysis of a C-terminal fragment AS 230–242. Spectra of the unlabelled (black) and complete ^15^N labelled references (red) are shown in comparison with the segmental isotopically labelled ^15^N(gpD)-Trx splice product (green) obtained through *in vivo* splicing with the *Ssp* DnaB intein.
(TIF)

Figure S9 Biosynthesis of the antibiotic Gramicidin S. Two NRPS multi-domain proteins are responsible for the formation of Gramicidin S, namely Gramicidin S Synthetase I (GrsA) and Gramicidin S Synthetase II (GrsB). In the first round, each module (one in GrsA and four in GrsB) incorporates one amino acid into the growing peptide chain tethered as thioesters on the phosphopantetheinyl group of the peptidyl carrier protein (PCP) domains. This leads to a pentapeptide (*D*-Phe-Pro-Val-Orn-Leu) which is transferred onto the thioesterase (TE) domain. After a second round of pentapeptide formation, the two peptides are dimerized and cyclized in a head to tail manner to yield Gramicidin S.
(TIF)

Figure S10 Integration of the *Ssp* DnaB intein cassette into ST-GrsB1-His_6_. Western blot analysis of the four of flanking amino acids combinations at the splice junction (GrsB1 ssp1–4, Figure 4B). Arabinose induction lasted for 2 h; with an additional 3 h for the IPTG double-induction. Purified WT ST-GrsB1-His_6_ protein is indicated as pos. The theoretical molecular masses of the proteins are as follows: **SP** = 124.2 kDa; **Part N** = 124.9 kDa; **Part C** = 16.7 kDa.
(TIF)

Figure S11 Scheme of the formation of D-Phe-L-Pro-DKP with the first two modules of Gramicidin S biosynthesis, GrsA and GrsB1.
(TIF)

Figure S12 The uroporphyrinogen III methyltransferase (CobA). A) Reaction pathway of the uroporphyrinogen III methyltransferase (CobA). CobA catalyzes the conversion of uroporphyrinogen III to precorrin-2 through the consumption of two molecules of SAM. An overproduction of CobA results in an accumulation of the red fluorescent compounds sirohydrochlorin and trimethylpyrrocorphin. (A = acetate, P = propionate). B) Sequence alignment of uroporphyrinogen III methyltransferases from diverse organisms. Complete invariant residues are coloured in red, conserved residues with at least 8 out of 13 are shown in green; the two insertion positions of the *Npu* DnaE intein are indicated. C) Crystal structure of the uroporphyrin III methyltransferase from *Thermus thermophilus* (pdb-code 1V9A [51]). After sequence alignment with the uroporphyrinogen III methyltransferase of *P. freudenreichii*, the equivalent insertion positions are indicated.
(TIF)

Figure S13 SDS-PAGE analysis of the expression and subsequent purification of full length and spliced ST-CobA-His_6_ proteins at different temperatures. A) The mutant protein ST-CobA110FN-His_6_. B) CobA splice product formation (identically to ST-CobA_110FN_-His_6_) after co-induction of both fusion genes. (I = insoluble fraction after cell lysis; S = soluble fraction after cell lysis; elution = the first three elution fractions of the Ni^2+^-NTA affinity chromatography)
(TIF)

Table S1 Plasmids generated after the integration of the *Ssp* DnaB intein cassette into gpD-Trx.
(TIF)

Table S2 Plasmids generated after the integration of the *Npu* DnaE intein cassette into gpD-Trx.
(TIF)

Table S3 Plasmids constructed in this study for identifying an active split intein insertion in GrsB1^S961^.
(TIF)

Table S4 Plasmids constructed in this study for identifying an active split intein insertion in CobA^C109^ and in CobA^S159C^.
(TIF)

Table S5 Analysis of the generation of the model protein integration plasmids via RF-PCR.
(TIF)

File S1 (DOC)
