# Supplementary figures and images for: SPLICEFINDER – A Fast and Easy Screening Method for Active Protein Trans-Splicing Positions
Source: PLoS One. 2013 Sep 2;8(9):e72925. doi: 10.1371/journal.pone.0072925 (PMC3759424; doi:10.1371/journal.pone.0072925)

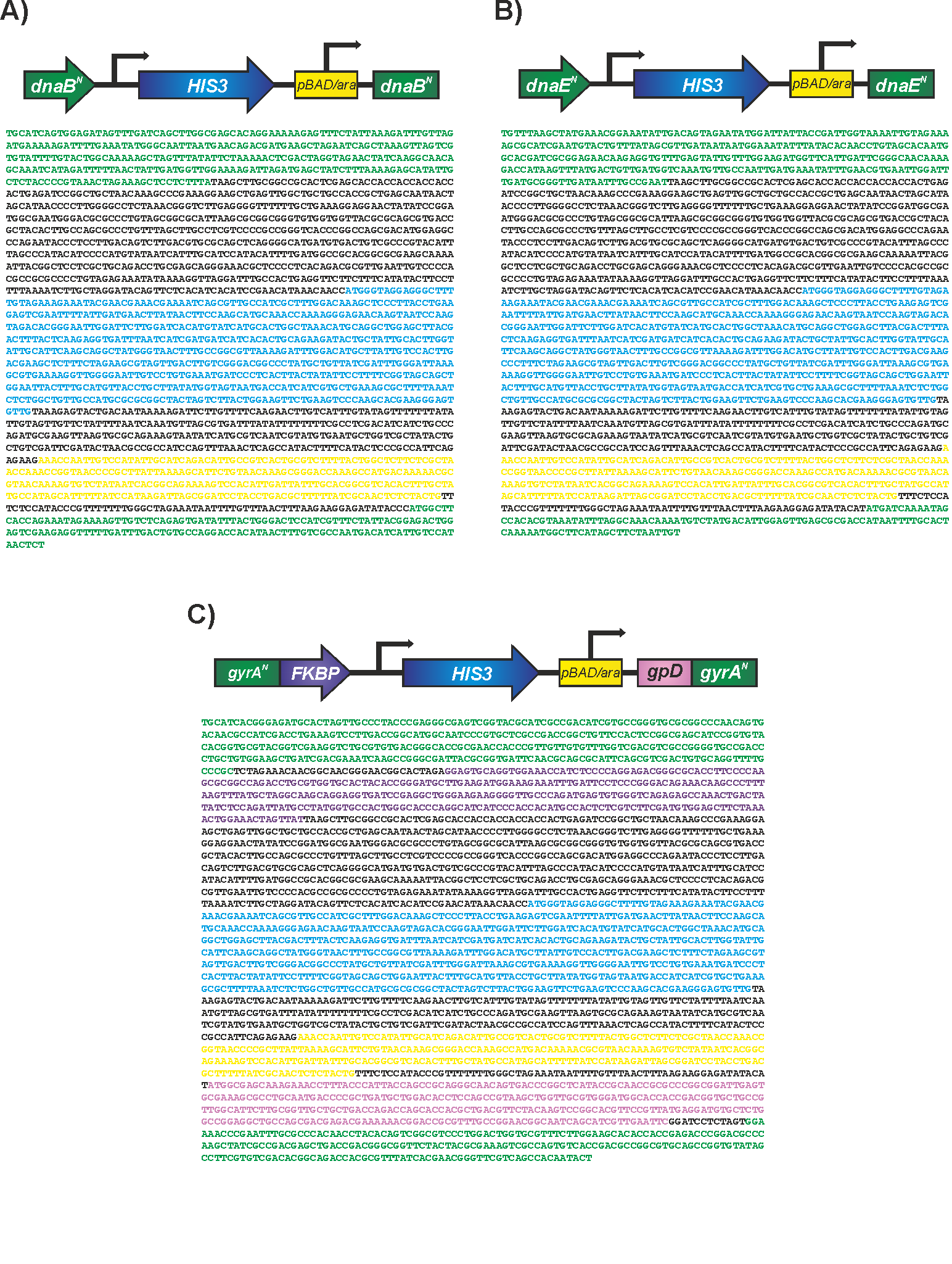

Supplement: Figure S1 — DNA-sequences of the intein cassettes. Sequences are shown from position 1 of IntN to position +1 of IntC. The plasmid carrying the Ssp DnaB intein cassette (2386 bp) is pCasDnaB2, the plasmid of the Npu DnaE intein cassette (2338 bp) is pCasDnaE2, and the plasmid encoding the Mxe GyrA intein cassette (3329 bp) is pCasGyrA2. (TIF) [file pone.0072925.s001.tif]

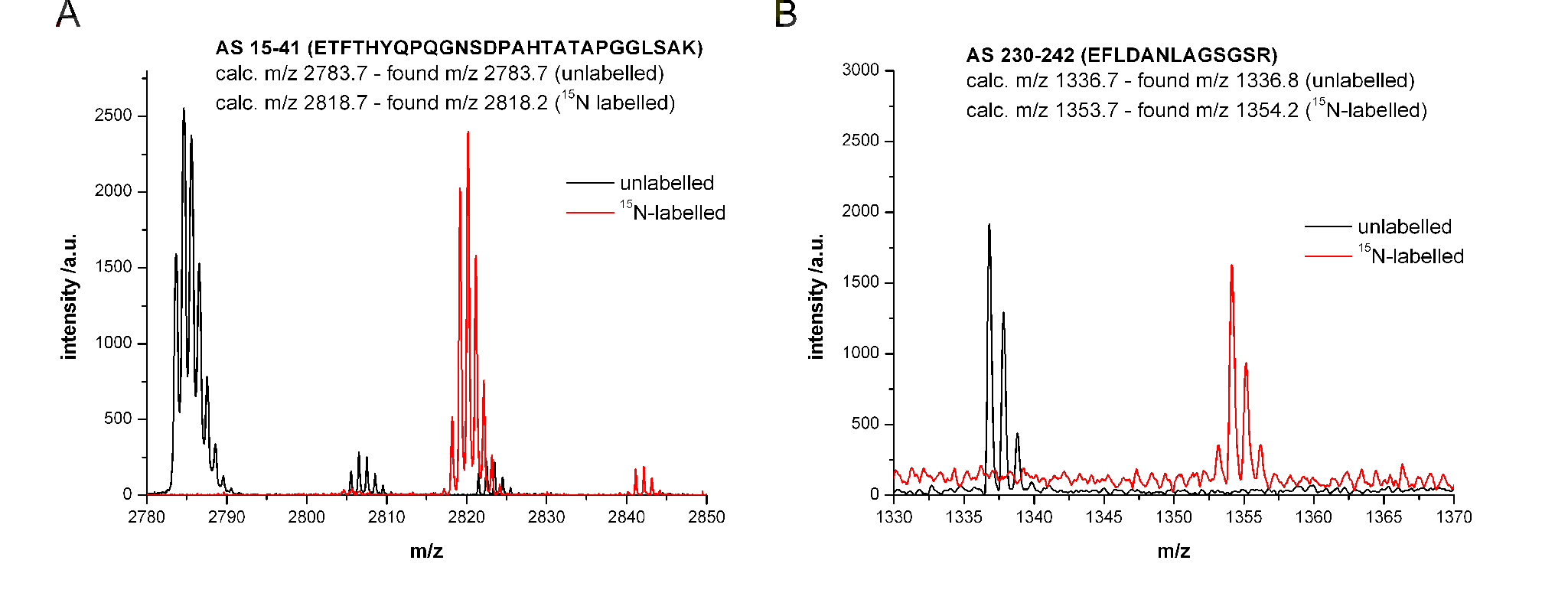

Supplement: Figure S2 — Labelling efficiency determination. MALDI-TOF MS analysis of an N-terminal A) and a C-terminal B) peptide fragment after tryptic digest of the unlabelled (black) and completely (red) 15N-labelled model protein ST-gpD-Trx-His6 (AS denotes amino acid sequence). (TIF) [file pone.0072925.s002.tif]

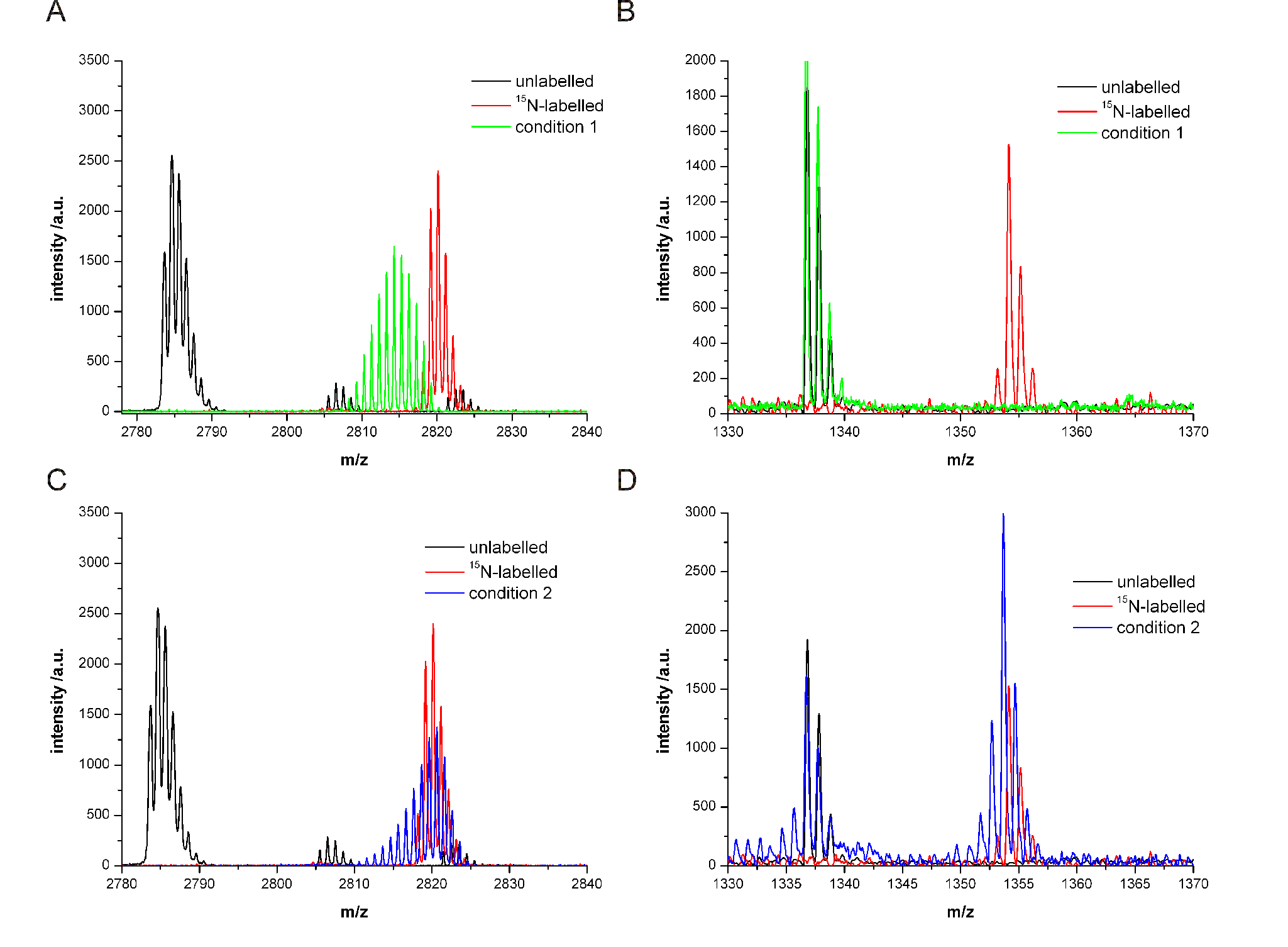

Supplement: Figure S3 — MALDI-TOF MS analysis of two different expression conditions for segmental isotopic labelling via in vivo PTS of the model protein ST-gpD-Trx-His6. A) and B) MS spectra for condition 1 (green); C) and D) MS spectra for condition 2 (blue). All spectra are shown in comparison with a completely unlabelled (black) and a completely 15N-labelled (red) sample. A) and C) show an N-terminal fragment (amino acid sequence 15–41) and B) and D) show a C-terminal fragment (amino acid sequence 230–242) (for details on the expression conditions and MS analysis see text). (TIF) [file pone.0072925.s003.tif]

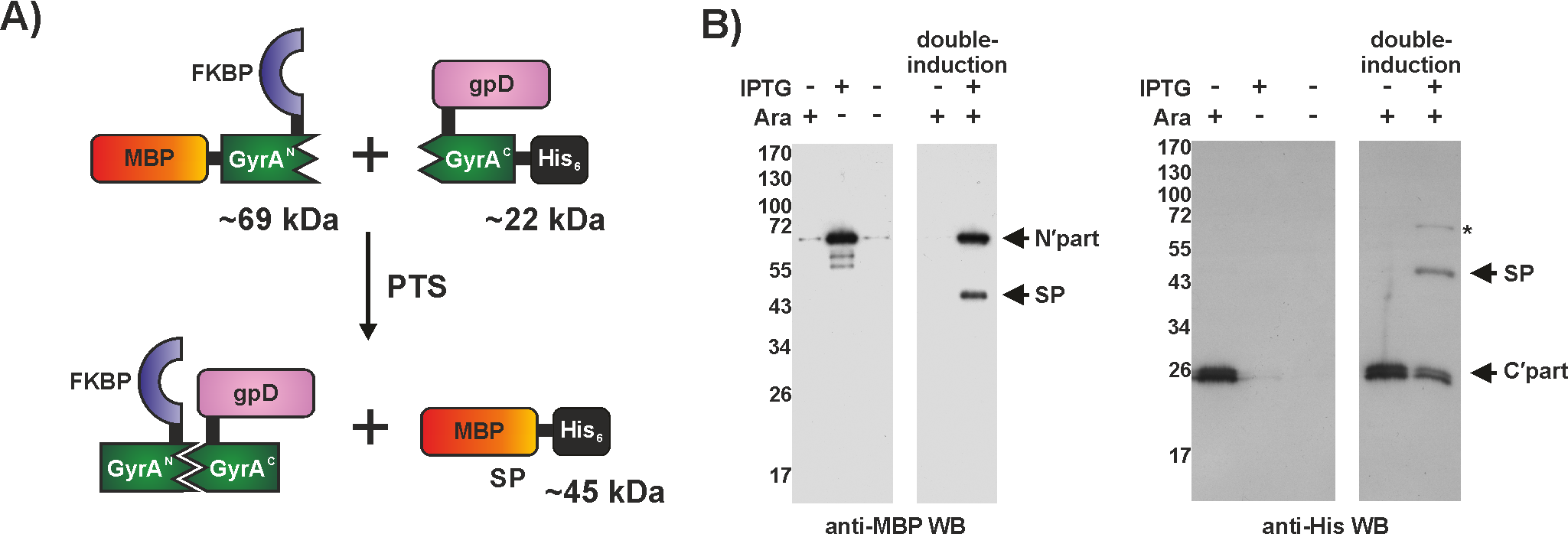

Supplement: Figure S4 — The Mxe GyrA intein cassette. A) Schematic representation of the Mxe GyrA intein cassette mediated splice reaction B) Western blot analysis of small-scale expression of E. coli cells containing the intein cassette plasmid, as well as the helper plasmid pRSFara. The single inductions were done for 4 h at 37°C (0.2% arabinose or 0.4 mM IPTG). The dual inductions: 0.2% arabinose for 2 h at 37°C, then media exchange, and subsequent induction with 0.4 mM IPTG for 4 h, 25°C. The theoretical molecular masses of the proteins are: SP = 44.7 kDa; N’Part = 69.3 kDa; C’Part = 22.2 kDa. (TIF) [file pone.0072925.s004.tif]

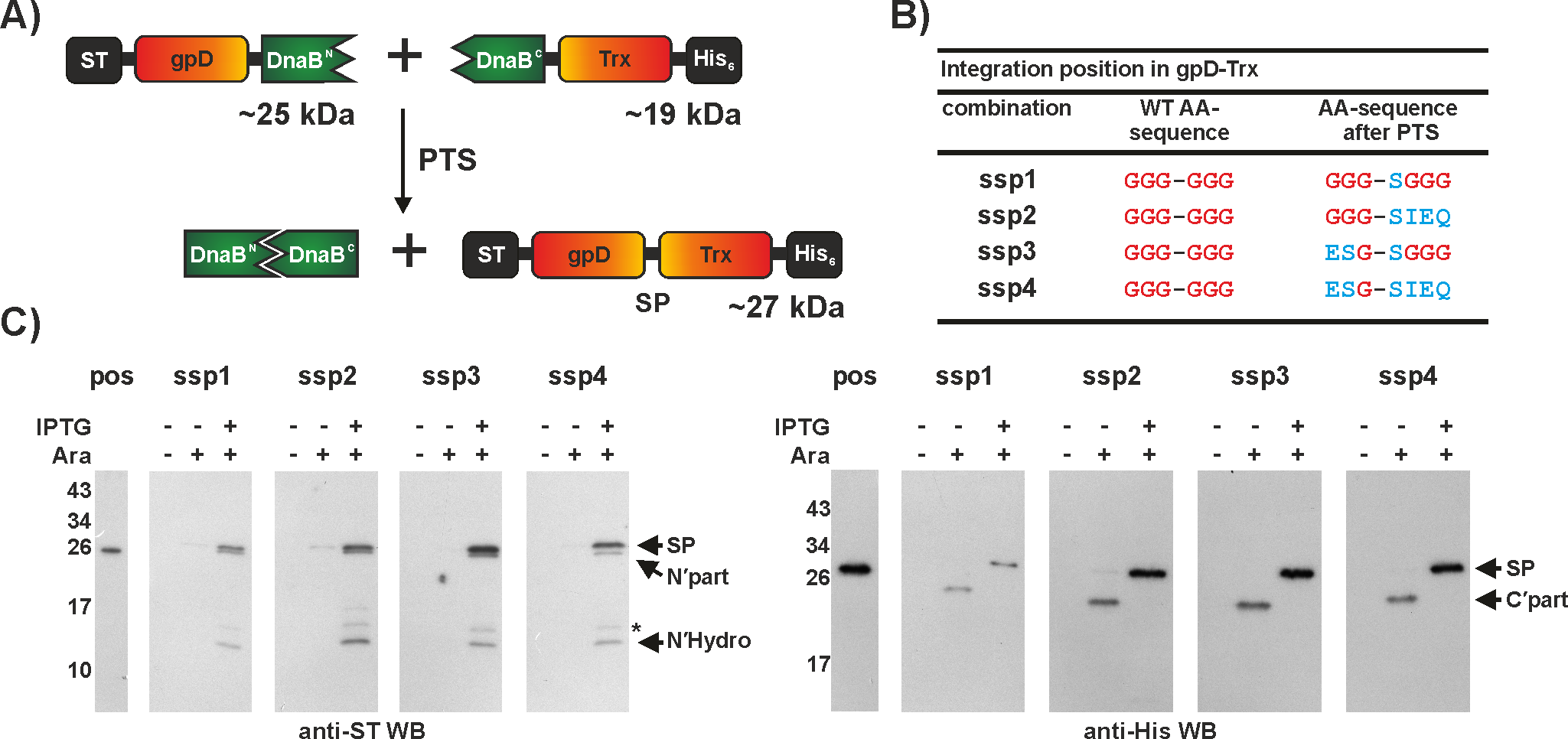

Supplement: Figure S5 — Integration of the Ssp DnaB intein cassette into gpD-Trx. A) Schematic representation of the PTS reaction after the integration of the Ssp DnaB PTS cassette into ST-gpD-Trx-His6. B) Amino acid sequences at the splice junctions for the produced combinations in the linker region of ST-gpD-Trx-His6. Amino acids deviations after splicing from the original sequence (WT AA-sequence) are shown in blue. C) Western blot analysis of the four different flanking amino acids variations at the splice junction. All four combinations are splice active. The calculated molecular weights of the proteins are as follows: SP = 26.5–26.8 kDa; N’Part = 24.9–25.0 kDa; C’Part = 18.8–19.0 kDa; N’Hydro = 13.2 kDa. (pos = full length ST-gpD-Trx-His6). (TIF) [file pone.0072925.s005.tif]

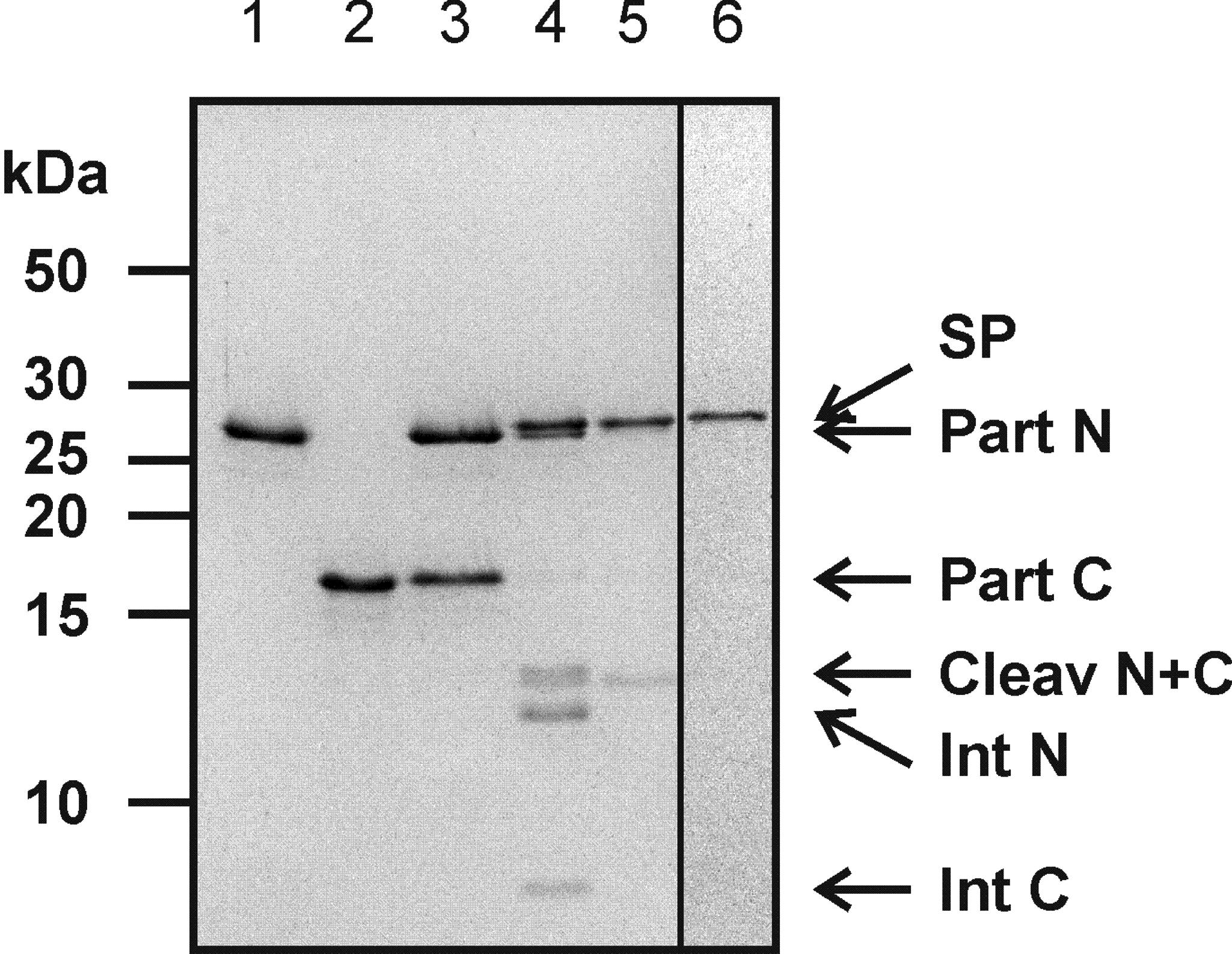

Supplement: Figure S6 — In vitro PTS to obtain segmental labelled ST-gpD-15N(Trx-His6) with the Npu DnaE intein. The SDS-PAGE gel of the PTS reaction and of the purification steps is shown in the Coomassie-staining. Lane 1: purified N-terminal part ST-gpD-IntN; lane 2: purified C-terminal part IntC-Trx-His6; lane 3: PTS-reaction at 0h; lane 4: PTS-reaction at 16 h; lane 5: combined elution fractions after Ni2+-NTA chromatography; lane 6: combined elution fractions after Strep-Tactin purification. The theoretical molecular masses of the proteins are as follows: SP = 26.7 kDa; Part N = 25.0 kDa; Part C = 17.6 kDa; Cleav N = 13.2 kDa; Cleav C = 13.5 kDa; Int N = 11.9 kDa; Int C = 4.1 kDa. (TIF) [file pone.0072925.s006.tif]

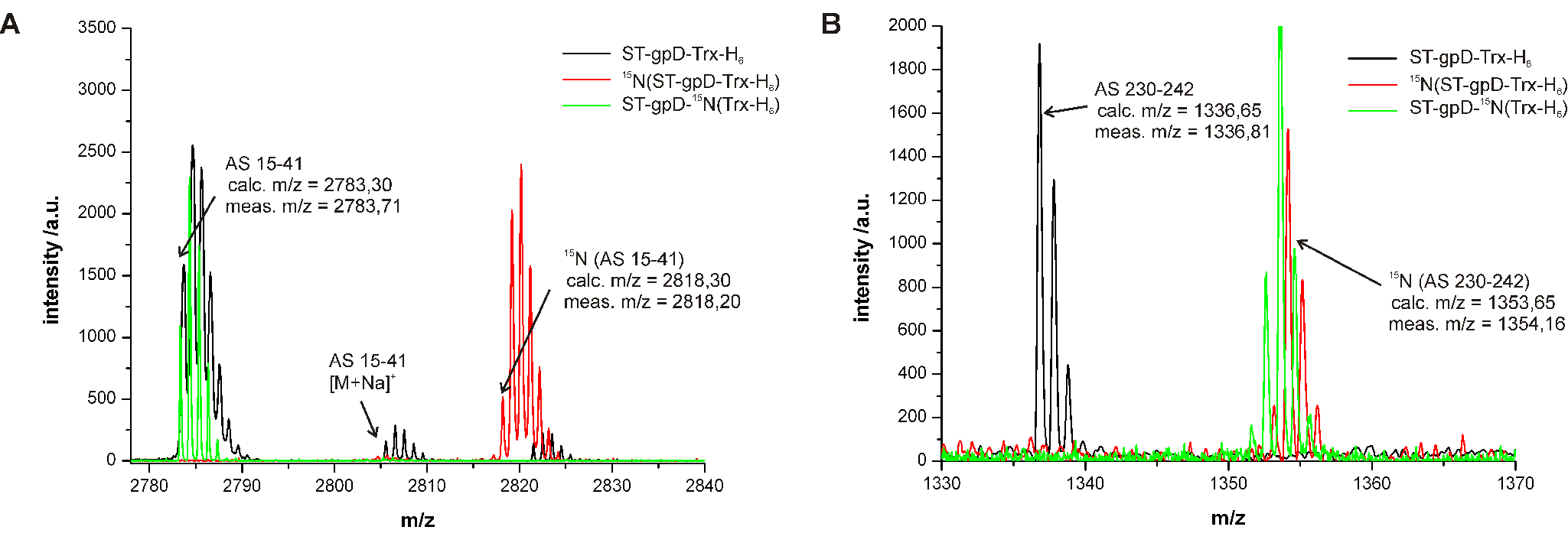

Supplement: Figure S7 — MALDI-TOF MS analysis of the segmental labelled gpD-15N(Trx) splice product. A) Analysis of an N-terminal fragment AS 15–41, B) Analysis of a C-terminal fragment AS 230–242. Spectra of the unlabelled (black) and complete 15N labelled references (red) are shown in comparison with the segmental isotopically labelled gpD-15N(Trx) splice product (green) obtained through in vitro splicing with the Npu DnaE intein. (TIF) [file pone.0072925.s007.tif]

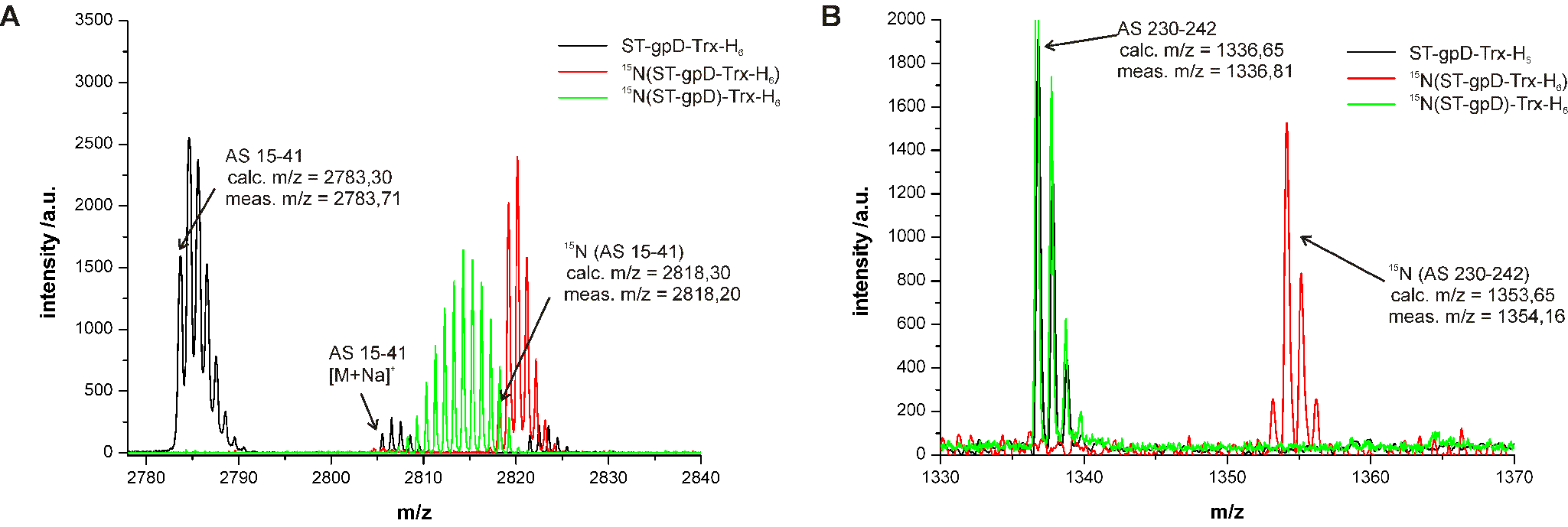

Supplement: Figure S8 — MALDI-TOF MS analysis of the segmental labelled 15N(gpD)-Trx splice product. A) Analysis of an N-terminal fragment AS 15–41, B) Analysis of a C-terminal fragment AS 230–242. Spectra of the unlabelled (black) and complete 15N labelled references (red) are shown in comparison with the segmental isotopically labelled 15N(gpD)-Trx splice product (green) obtained through in vivo splicing with the Ssp DnaB intein. (TIF) [file pone.0072925.s008.tif]

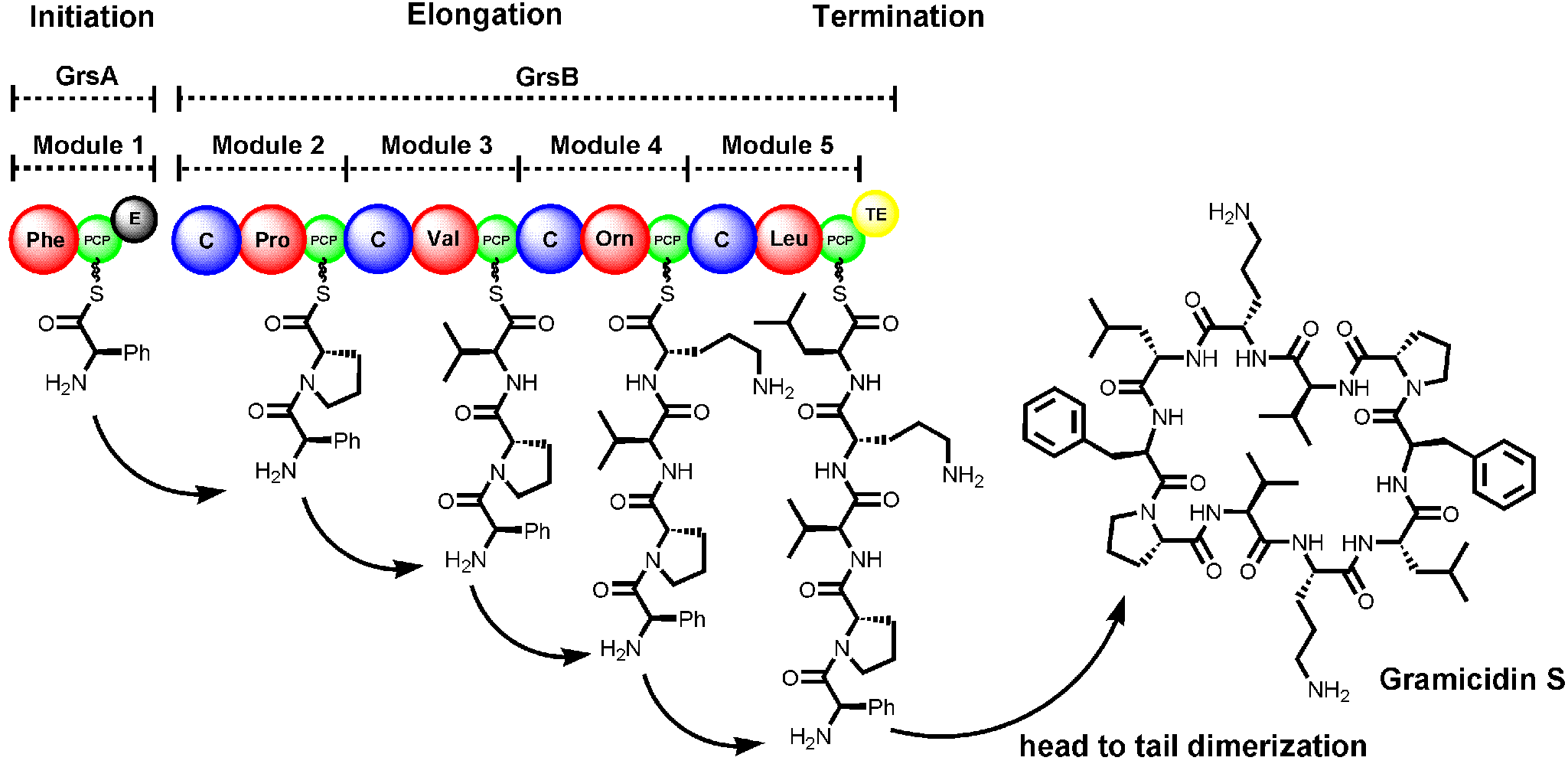

Supplement: Figure S9 — Biosynthesis of the antibiotic Gramicidin S. Two NRPS multi-domain proteins are responsible for the formation of Gramicidin S, namely Gramicidin S Synthetase I (GrsA) and Gramicidin S Synthetase II (GrsB). In the first round, each module (one in GrsA and four in GrsB) incorporates one amino acid into the growing peptide chain tethered as thioesters on the phosphopantetheinyl group of the peptidyl carrier protein (PCP) domains. This leads to a pentapeptide (D-Phe-Pro-Val-Orn-Leu) which is transferred onto the thioesterase (TE) domain. After a second round of pentapeptide formation, the two peptides are dimerized and cyclized in a head to tail manner to yield Gramicidin S. (TIF) [file pone.0072925.s009.tif]

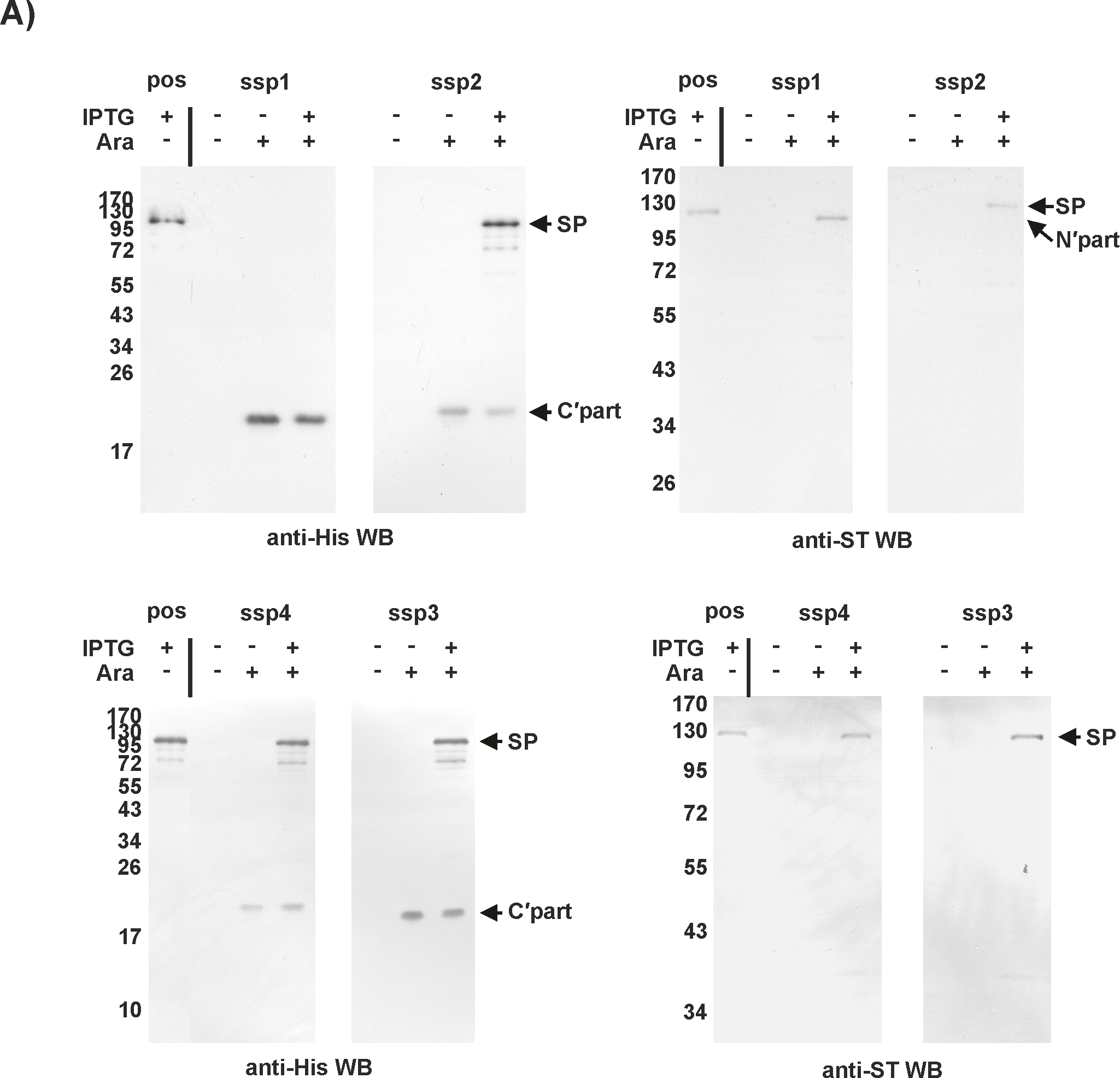

Supplement: Figure S10 — Integration of the Ssp DnaB intein cassette into ST-GrsB1-His6. Western blot analysis of the four of flanking amino acids combinations at the splice junction (GrsB1 ssp1–4, Figure 4B). Arabinose induction lasted for 2 h; with an additional 3 h for the IPTG double-induction. Purified WT ST-GrsB1-His6 protein is indicated as pos. The theoretical molecular masses of the proteins are as follows: SP = 124.2 kDa; Part N = 124.9 kDa; Part C = 16.7 kDa. (TIF) [file pone.0072925.s010.tif]

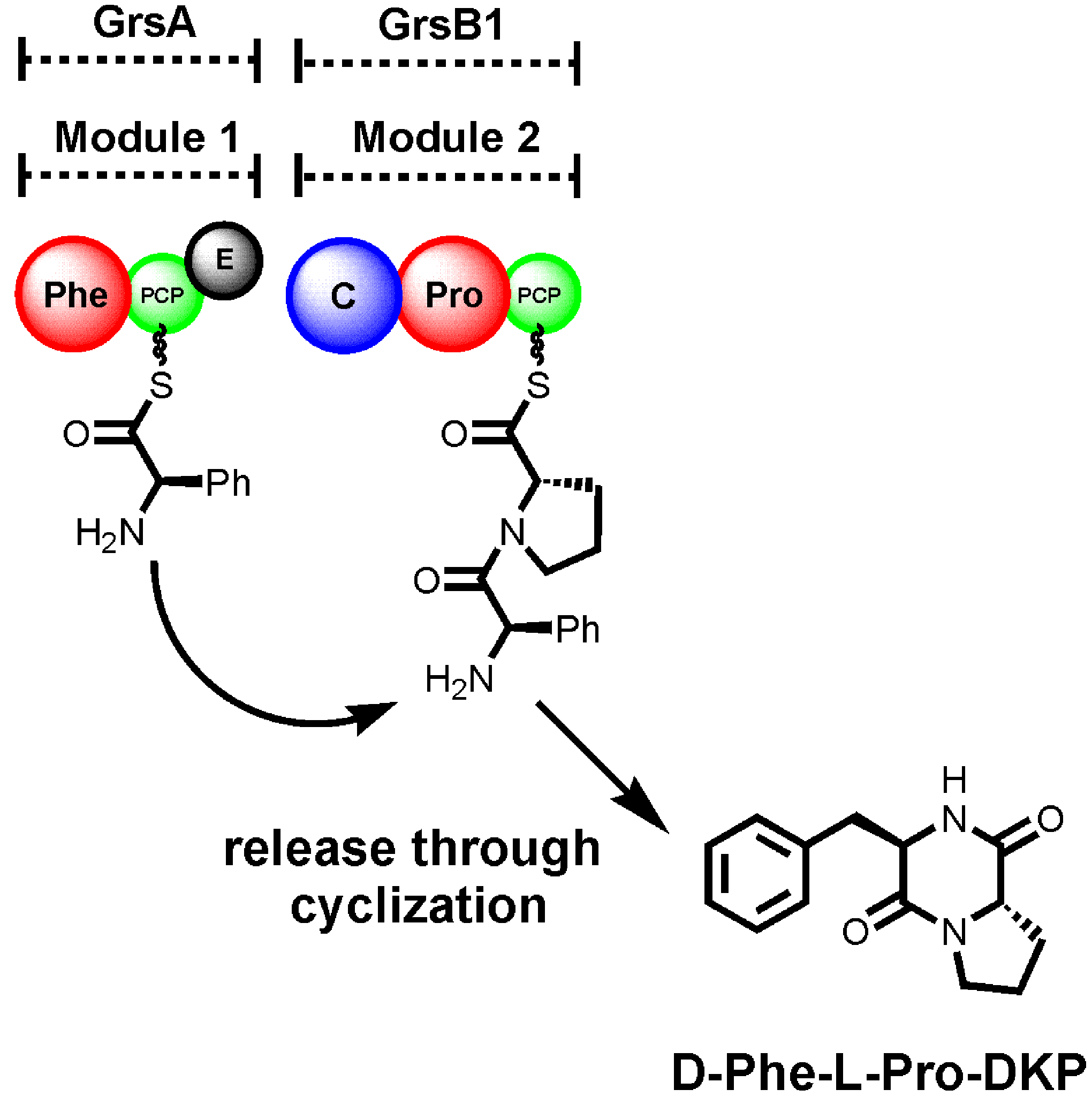

Supplement: Figure S11 — Scheme of the formation of D-Phe-L-Pro-DKP with the first two modules of Gramicidin S biosynthesis, GrsA and GrsB1. (TIF) [file pone.0072925.s011.tif]

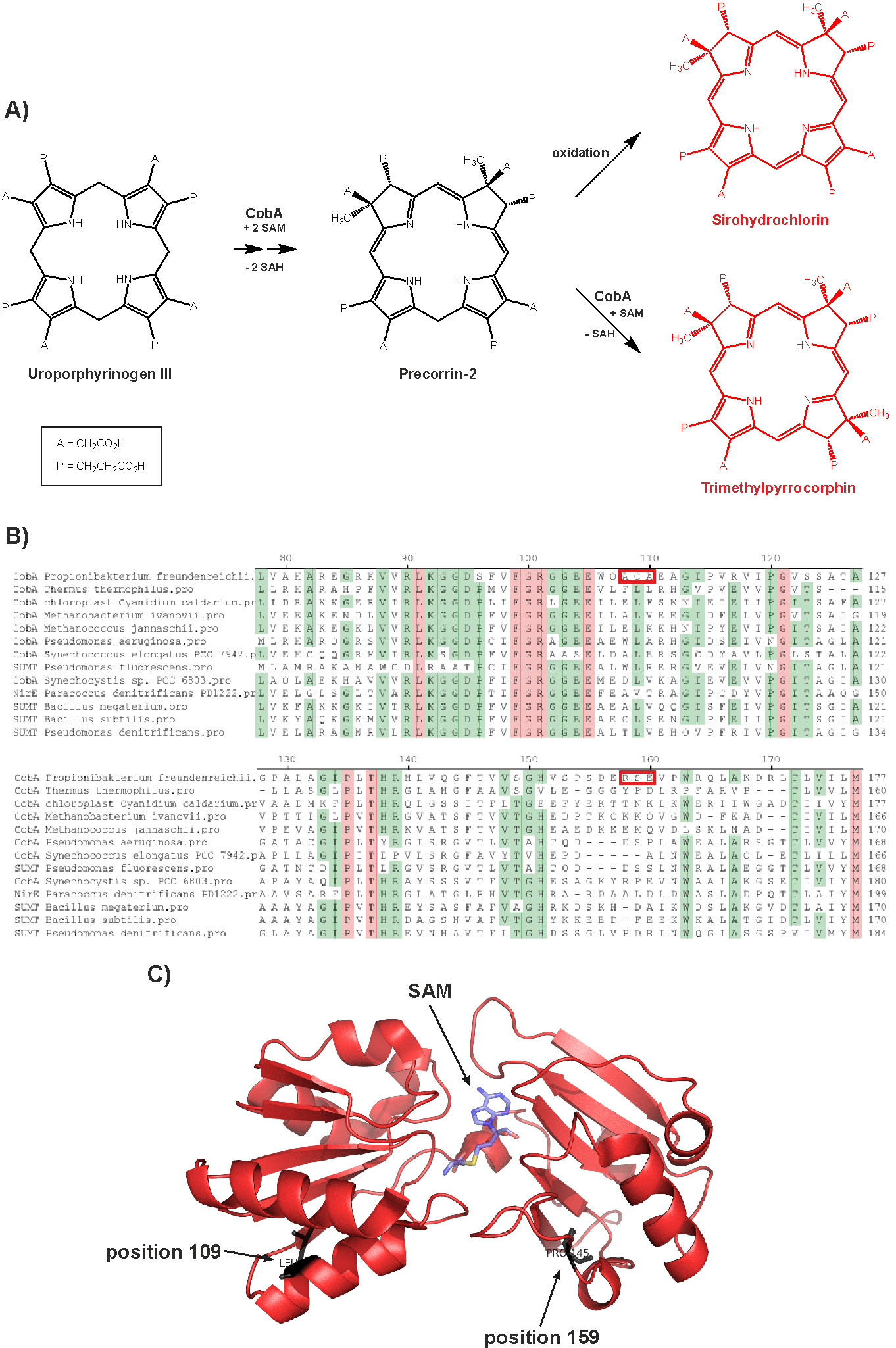

Supplement: Figure S12 — The uroporphyrinogen III methyltransferase (CobA). A) Reaction pathway of the uroporphyrinogen III methyltransferase (CobA). CobA catalyzes the conversion of uroporphyrinogen III to precorrin-2 through the consumption of two molecules of SAM. An overproduction of CobA results in an accumulation of the red fluorescent compounds sirohydrochlorin and trimethylpyrrocorphin. (A = acetate, P = propionate). B) Sequence alignment of uroporphyrinogen III methyltransferases from diverse organisms. Complete invariant residues are coloured in red, conserved residues with at least 8 out of 13 are shown in green; the two insertion positions of the Npu DnaE intein are indicated. C) Crystal structure of the uroporphyrin III methyltransferase from Thermus thermophilus (pdb-code 1V9A [51]). After sequence alignment with the uroporphyrinogen III methyltransferase of P. freudenreichii, the equivalent insertion positions are indicated. (TIF) [file pone.0072925.s012.tif]

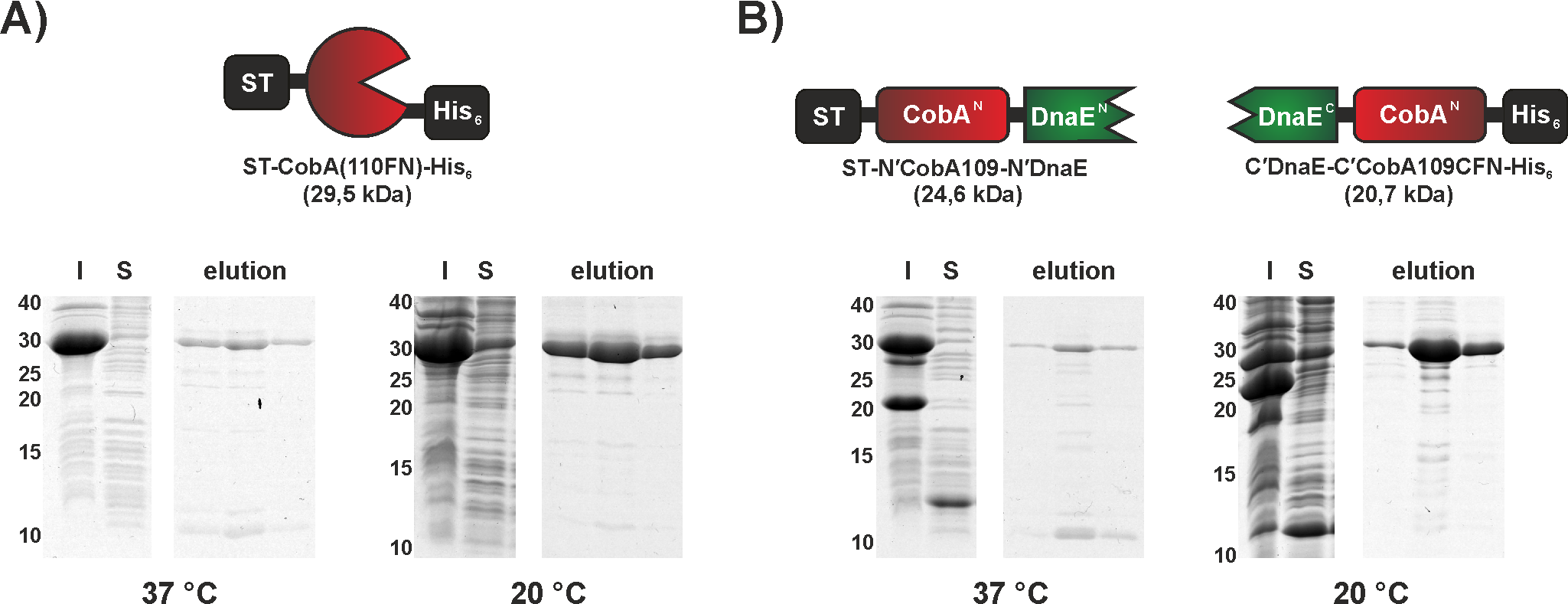

Supplement: Figure S13 — SDS-PAGE analysis of the expression and subsequent purification of full length and spliced ST-CobA-His6 proteins at different temperatures. A) The mutant protein ST-CobA110FN-His6. B) CobA splice product formation (identically to ST-CobA110FN-His6) after co-induction of both fusion genes. (I = insoluble fraction after cell lysis; S = soluble fraction after cell lysis; elution = the first three elution fractions of the Ni2+-NTA affinity chromatography) (TIF) [file pone.0072925.s013.tif]

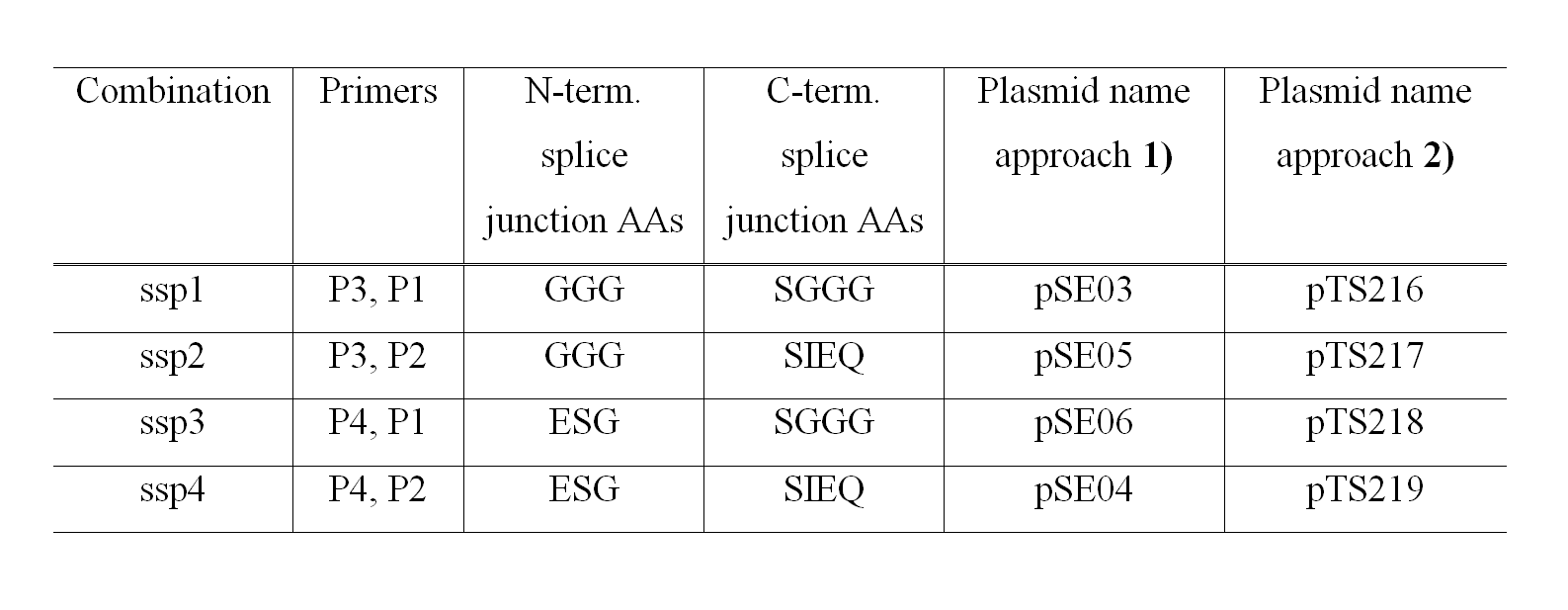

Supplement: Table S1 — Plasmids generated after the integration of the Ssp DnaB intein cassette into gpD-Trx. (TIF) [file pone.0072925.s014.tif]

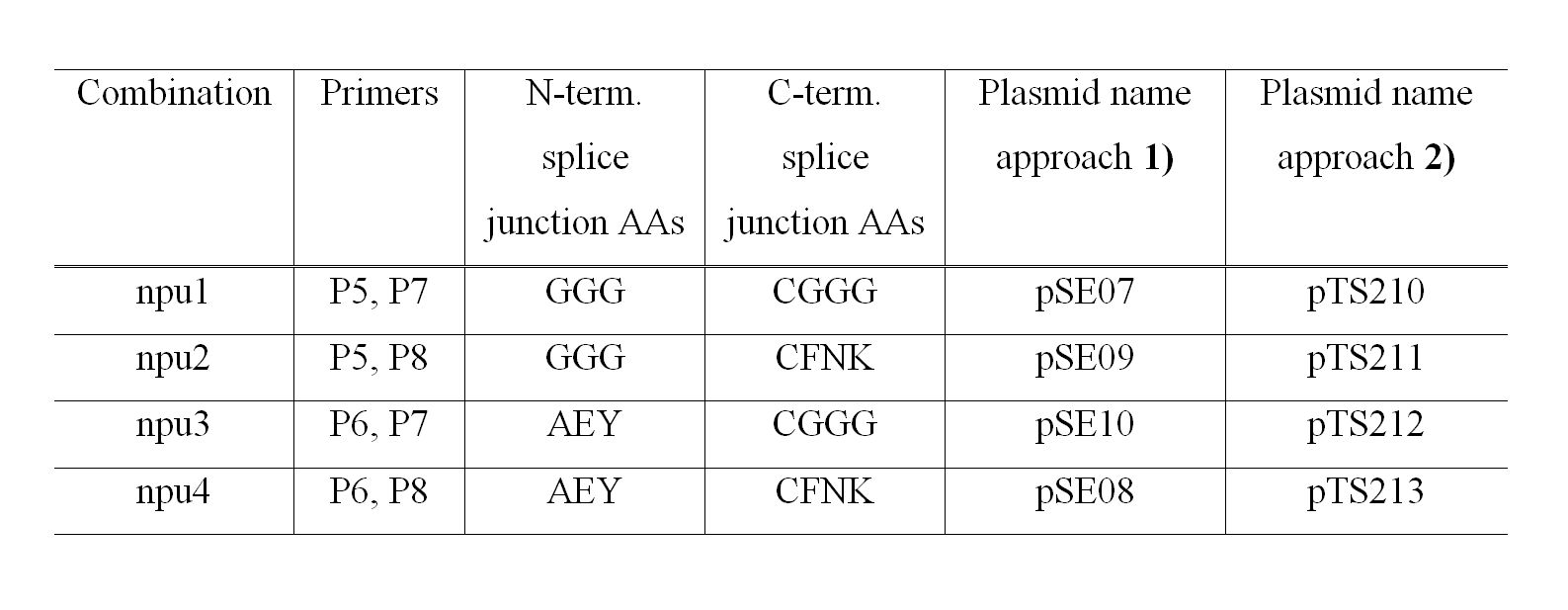

Supplement: Table S2 — Plasmids generated after the integration of the Npu DnaE intein cassette into gpD-Trx. (TIF) [file pone.0072925.s015.tif]

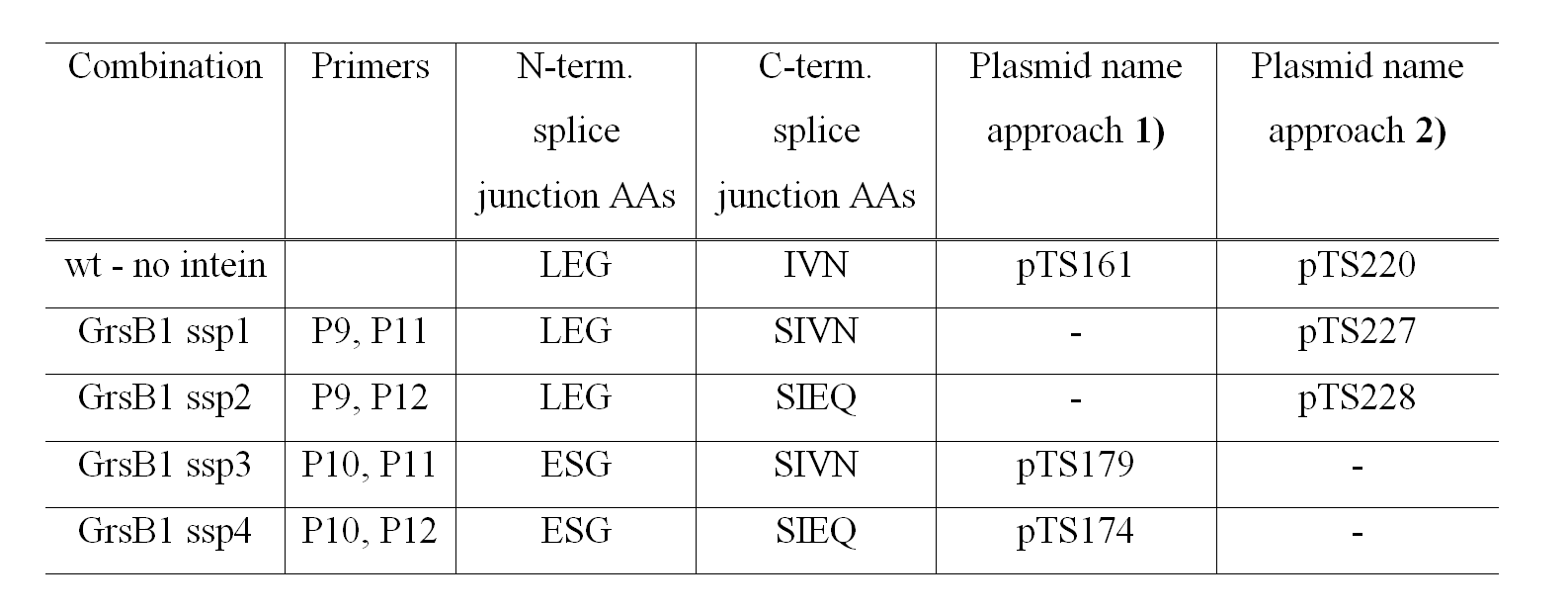

Supplement: Table S3 — Plasmids constructed in this study for identifying an active split intein insertion in GrsB1S961. (TIF) [file pone.0072925.s016.tif]

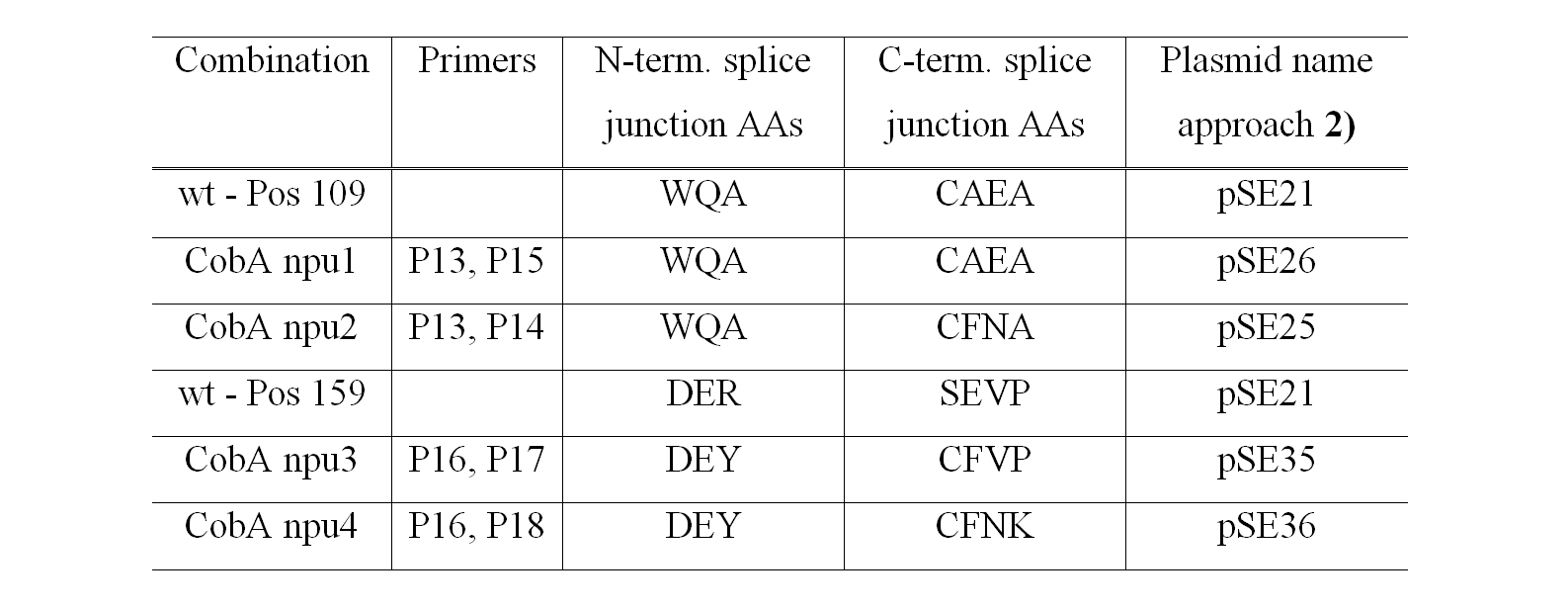

Supplement: Table S4 — Plasmids constructed in this study for identifying an active split intein insertion in CobAC109 and in CobAS159C. (TIF) [file pone.0072925.s017.tif]

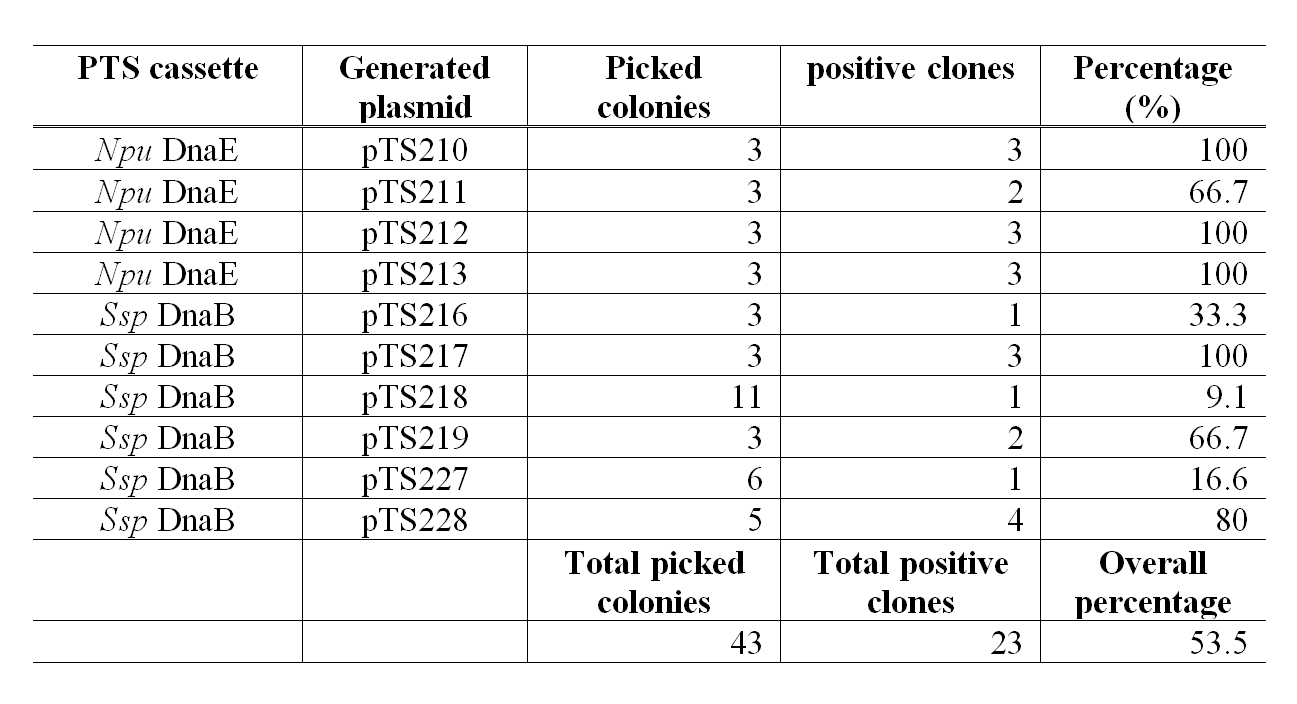

Supplement: Table S5 — Analysis of the generation of the model protein integration plasmids via RF-PCR. (TIF) [file pone.0072925.s018.tif]
