# Supplementary material for: SPLICEFINDER – A Fast and Easy Screening Method for Active Protein Trans-Splicing Positions
Source: PLoS One. 2013 Sep 2;8(9):e72925. doi: 10.1371/journal.pone.0072925 (PMC3759424; doi:10.1371/journal.pone.0072925)
Supplement: File S1 — (DOC) [file pone.0072925.s019.doc]

**SUPPLEMENTARY INFORMATION**

Joachim Zettler^1,a,#^, Simone Eppmann^1^, Alena Busche^2^, Dina Dikovskaya^b^, Volker Dötsch^2^, Henning D. Mootz^1,c^, Tim Sonntag^1,d,#,*^

(^#^) these authors contributed equally to this work

Contribution from 1:

Department of Chemistry and Chemical Biology, TU Dortmund University, Dortmund, Germany

Contribution from 2:

Institute of Biophysical Chemistry and Center for Biomolecular Magnetic Resonance, Goethe University, Frankfurt/Main, Germany

Present addresses:

(a) Ascendis Pharma GmbH, Heidelberg, Germany

(b) CRUK Beatson Laboratories, University of Glasgow, Glasgow, United Kingdom

(c) Institute of Biochemistry, University of Muenster, Münster, Germany

(d) Peptide Biology Laboratories, The Salk Institute for Biological Studies, La Jolla, California, United States of America

(*) To whom correspondence should be addressed. E-mail: [tsonntag@salk.edu](mailto:tsonntag@salk.edu)

**Table of Contents**

Material and Methods [3](#__RefHeading___Toc360962330)

Plasmid construction [3](#__RefHeading___Toc360962331)

Split intein cassettes [3](#__RefHeading___Toc360962332)

Plasmids encoding the model gene gpD-Trx [5](#__RefHeading___Toc360962333)

The helper plasmid pRSFara (*lacI* and *araC*) [5](#__RefHeading___Toc360962334)

Integration of the *Ssp* DnaB and the *Np*u DnaE split inteins into gpD-Trx [6](#__RefHeading___Toc360962335)

Plasmids for the individual expression of the *Npu* DnaE intein-fusion proteins [7](#__RefHeading___Toc360962336)

Plasmids encoding the NRPS genes *grsB1* and *grsA* [7](#__RefHeading___Toc360962337)

Integration of the *Ssp* DnaB split intein cassette into *grsB*1 [8](#__RefHeading___Toc360962338)

Plasmid encoding for the uroporphyrinogen III methyltransferase CobA [9](#__RefHeading___Toc360962339)

Integration of the *Npu* DnaE split intein cassette into *cobA* [10](#__RefHeading___Toc360962340)

Yeast intein cassette integration [11](#__RefHeading___Toc360962341)

Restriction-free (RF) cloning - intein cassette integration [11](#__RefHeading___Toc360962342)

Small-scale expression protocol [12](#__RefHeading___Toc360962343)

Protein purification of the individual Npu DnaE fusion proteins [13](#__RefHeading___Toc360962344)

Western Blot [14](#__RefHeading___Toc360962345)

NMR spectroscopy [14](#__RefHeading___Toc360962346)

Tryptic digest of SDS-PAGE bands [14](#__RefHeading___Toc360962347)

Determination of the isotopic labelling efficiency via MALDI-TOF MS analysis [15](#__RefHeading___Toc360962348)

Activity test for GrsB1 proteins - formation of D-Phe-L-Pro-Diketopiperazine [18](#__RefHeading___Toc360962349)

CobA activity test in *E. coli* [18](#__RefHeading___Toc360962350)

Extended Experimental Procedures [20](#__RefHeading___Toc360962351)

The *Mxe* GyrA intein cassette [20](#__RefHeading___Toc360962352)

Integration of the Ssp DnaB intein into the model protein gpD-Trx [21](#__RefHeading___Toc360962353)

Preparation of ^15^N-labelled gpD-Trx samples for NMR studies [22](#__RefHeading___Toc360962354)

Integration of the Ssp DnaB intein into the complete non-ribosomal peptide synthetase module GrsB1 [25](#__RefHeading___Toc360962355)

Integration of the Npu DnaE intein into the uroporphyrinogen III methyltransferase CobA [30](#__RefHeading___Toc360962356)

Acknowledgements [34](#__RefHeading___Toc360962357)

Literature [34](#__RefHeading___Toc360962358)

# Material and Methods

## Plasmid construction

### Split intein cassettes

All intein cassettes are based on a plasmid, which was generated via a fusion PCR (split VMA intein divided by HIS3 into pBluescript), where the HIS3 marker with its promoter and terminator was amplified from the pFA6a-His3MX6 plasmid . The resulting plasmid was termed pTS13 . In subsequent RF-PCRs the 5’- and 3’-terminal regions were replaced with intein fusion constructs (see Restriction-free (RF) PCR cloning for- intein cassette integration for protocol). In the case of the *Ssp* DnaB intein cassette the C-coding region was amplified via PCR from pAU04 (*Ssp* DnaB^C^ (105-154)-GrsA A^Sub B^-PCP-His_6_) with the primers (5’-TACTAACGCCG CCATCCAGTT TAAACTCAGC CATACTTTTC ATACTCC) and (5’-CCCTCACTAA AGGGAACAAA AGCTGGCGGA TACATATTTG AATGTAT). The PCR product included the araBAD promoter region of the commercially available pBAD expression system (Invitrogen) and was used as the insert in a RF-PCR with pTS13 resulting in the plasmid pCasDnaB1. A second RF-PCR of pCasDnaB1 with the PCR amplified insert from pJZ28 (His_6_-GrsA A^Sub A^- DnaB^N^(1-104)) using the primers (5’-AATACGACTC ACTATAGGGC GAATTGCGAG ATCTCGATCC CGCGAAAT) and (5’-ATCTGGCGCG CCTTAATTAA CCCGGGGCGC TGGCAAGTGT AGCGGTC) exchanged the 5’-terminal part and resulted in the actual *Ssp* DnaB intein cassette, pCasDnaB2. Two additional RF-PCR cloning steps were necessary to obtain the *Npu* DnaE intein cassette. First the gene sequence coding for the N-terminal region was substituted with the fragment ST-gpD-*Npu* DnaE^N^(1-102) which was amplified from pAU08 with the primers (5’-AATTGTGAGC GGATAACAAT TCCCCTGTAG AAATAATTTT G) and (5’-TGGTGGTGGT GCTCGAGTGC GGCCGCAAGC TTAATT). In the resulting plasmid pCasDnaE1 the C-terminal region was exchanged with an insert containing the sequence *Npu* DnaE^C^(103-138)-eGPF-His_6_ amplified from pAU07 with the primers (5’-CGCTTTTTAT CGCAACTCTC TACTG) and (5’-AAGCTTAGTG ATGGTGATGG TGATG). The final *Npu* DnaE intein cassette was termed pCasDnaE2. Analogous to the generation of the two former mentioned intein cassettes the *Mxe* GyrA intein cassette was obtained through two subsequent RF-PCR cloning steps from pCasDnaE2. The 5’-coding region was amplified with the primers (5’-ACTTTAATAA GGAGATATAC CATGGAAATC GAAGAAGG) and (5’-GTGCTCGAGT GCGGCCGCAA GCTTAATAAC TAGTTTCCAG TTTTAG) from pAI13 and contained the sequence coding for MBP-*Mxe* GyrA^N^(1-119)-FKBP. The intermediate plasmid was pCasGyrA1, which served as template in the second cloning step. Here the 3’-coding region was exchanged with the insert gpD-*Mxe* GyrA^C^(120-199)-His_6_ obtained from pDK01 with the primers (5’-ACTTTAAGAA GGAGATATAC ATATGGCGAG CAAAGAAACC TTTAC) and (5’-AAGCTTAGTG ATGGTGATGG TGATG). For the final cassette DNA sequences please consult Figure S1.


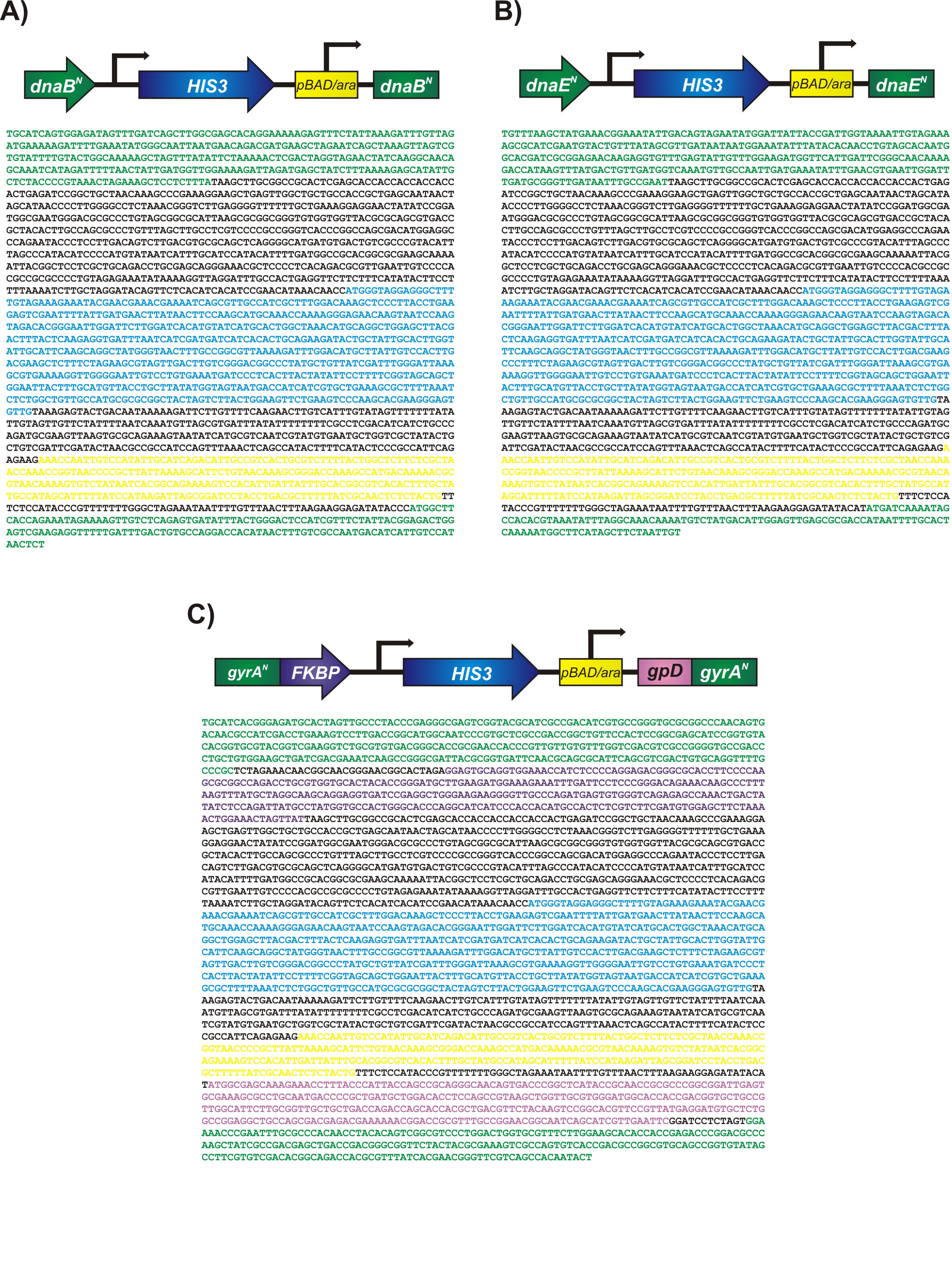


**Figure S1**.

**DNA-sequences of the intein cassettes**. Sequences are shown from position 1 of Int^N^ to position +1 of Int^C^. The plasmid carrying the *Ssp* DnaB intein cassette (2386 bp) is pCasDnaB2, the plasmid of the *Npu* DnaE intein cassette (2338 bp) is pCasDnaE2, and the plasmid encoding the *Mxe* GyrA intein cassette (3329 bp) is pCasGyrA2.

### Plasmids encoding the model gene gpD-Trx

In approach **1)** (homologous recombination in *S. cerevisiae* ) we used a modified p425-TDH shuttle vector in which the TDH promoter and CYC1 terminator regions were replaced via restriction-free PCR (RF-PCR) with the controlling elements of gene expression from the pET16b plasmid (Novagen). This plasmid was termed pTS160. In approach **2)**, the integration of the intein cassettes was done via RF-PCR and the commercially available pET16b vector (Novagen) was used. The plasmids coding for ST-gpD-Trx-His_6_ in approach **1)** and **2)** were assembled using a three fragment ligation. The vector fragments resulted from *Nco*I, *Xho*I digests of pTS160 and pET16b, respectively. An *Nco*I, *Eco*RI fragment coding for ST-gpD was obtained from the plasmid pJZ42 (ST-gpD-*Ssp* DnaB^N^(1-104)). The second fragment (Trx-His_6_) was generated via PCR from pCL20 as a template with the primers (5’-ATAGAATTCG GCGGAGGCGG AGGAGGATCT GATAAAAT) and (5’-TATCTCGAGT TAGTGATGGT GATGGTGATG AG) and a subsequent *Eco*RI, *Xho*I digest. The first primer introduced the additional six glycine linker codons. Ligation of the two insert fragments with the corresponding vector fragments gave pSE01 (approach **1)**) and pTS209 (approach **2)**). For high expression yields of the fusion protein for NMR studies we subcloned the insert via an *Nco*I, *Xho*I digest into a commercially available pET28a plasmid (Novagen) yielding pSE02.

### The helper plasmid pRSFara (*lacI* and *araC*)

After integration of the intein cassettes into a target gene, selective expressions are only possible in the presence of the two regulatory proteins LacI and AraC. In approach **1)**, e.g. in plasmid pTS160, both gene sequences coding for these proteins are missing. In approach **2)** the pET16b vector backbone contains the *lacI* gene. Therefore an additional helper plasmid is necessary, containing both regulatory proteins. The so called pRSFara was constructed from a commercially available RSFDuet vector (Novagen, kanamycin resistance, *lacI*) where both multiple cloning sites were replaced with the *araC* gen and the corresponding promoter region via RF-PCR. The insert for this cloning step was obtained through a PCR from a commercially available pBAD-vector and with the primers (5’-CGCTCTCCCT TATGCGACTC CTGCAGGAGA AACAGTAGAG AGTTGCG) and (5’-TTCGCAGCAG CGGTTTCTTT ACCAGGCCTG CCACCATACC CACGCCG). All test expressions of the integration plasmids either generated via approach **1)** or **2)** were carried out in cells containing the pRSFara plasmid.

### Integration of the *Ssp* DnaB and the *Np*u DnaE split inteins into gpD-Trx

The following primers were used in both approaches **1)** and **2)** to integrate the *Ssp* DnaB intein cassette into the model protein ST-gpD-Trx-His_6_. P1 (5’-AATCATCAGT CAGATGAATA ATTTTATCAG ATCCTCCTCC AGAGTTATGG ACAATGATG), P2 (5’-ATCAAAAGAA TCATCAGTCA GATGAATAAT TTTATCAGAC TGTTCAATAG AGTTATGGAC), P3 (5’-TGCCGGAACG GCAATCAGCA TCGTTGAATT CGGCGGAGGC TGCATCAGTG GAGATAGTT), P4 (5’-GCGTTTGCCG GAACGGCAAT CAGCATCGTT GAATTCGAAA GCGGATGCAT CAGTGGAGAT). First, the intein cassette was amplified from pCasDnaB2 using four different primer combinations as indicated in Table S1. And the plasmids generated either via homologous recombination in *S. cerevisiae* into plasmid pSE01 or after RF-PCR cloning using pTS209 are also shown in Table S1.

**Table S1 Plasmids generated after the integration of the *Ssp*** DnaB intein cassette into gpD-Trx.

| Combination | Primers | N-term. splice junction AAs | C-term. splice junction AAs | Plasmid name approach **1)** | Plasmid name approach **2)** |
| --- | --- | --- | --- | --- | --- |
| ssp1 | P3, P1 | GGG | SGGG | pSE03 | pTS216 |
| ssp2 | P3, P2 | GGG | SIEQ | pSE05 | pTS217 |
| ssp3 | P4, P1 | ESG | SGGG | pSE06 | pTS218 |
| ssp4 | P4, P2 | ESG | SIEQ | pSE04 | pTS219 |

We also tested four different combinations of flanking amino acids to integrate the *Npu* DnaE intein cassette into the model gene. The used primer sequences were as followed. P5 (5’- TGCCGGAACG GCAATCAGCA TCGTTGAATT CGGCGGAGGC TGTTTAAGCT ATGAAACGG), P6 (5’-GACCGCGTTT GCCGGAACGG CAATCAGCAT CGTTGAATTC GCGGAGTACT GTTTAAGCTA TGAAAC), P7 (5’-AATCATCAGT CAGATGAATA ATTTTATCAG ATCCTCCTCC ACAATTAGAA GCTATGAAGC), and P8 (5’-ATCAAAAGAA TCATCAGTCA GATGAATAATT TTATCAGATT TATTGAAACA ATTAGAAGC). The intein cassette was amplified from pCasDnaE2 with the primer combinations shown in Table S2. The corresponding names of the integration plasmids, as well as the resulting flanking amino acids are also listed.

**Table S2 Plasmids generated after the integration of the *Npu*** DnaE intein cassette into gpD-Trx.

| Combination | Primers | N-term. splice junction AAs | C-term. splice junction AAs | Plasmid name approach **1)** | Plasmid name approach **2)** |
| --- | --- | --- | --- | --- | --- |
| npu1 | P5, P7 | GGG | CGGG | pSE07 | pTS210 |
| npu2 | P5, P8 | GGG | CFNK | pSE09 | pTS211 |
| npu3 | P6, P7 | AEY | CGGG | pSE10 | pTS212 |
| npu4 | P6, P8 | AEY | CFNK | pSE08 | pTS213 |

### Plasmids for the individual expression of the *Npu* DnaE intein-fusion proteins

For individual expression and purification of the *Npu* DnaE intein-fusion protein halves, their coding DNA sequences were cut out of the integration plasmids and cloned into expression vectors. The vector fragment for the 5’-fusion gene resulted from an *Nco*I, *Hind*III digest of a commercially available pRSFDuet vector (Novagen) and was ligated with *Nco*I, *Hind*III fragments of either pSE07 (ST-gpD-GGG-*Npu* DnaE^N^) or pSE10 (ST-gpD-AEY-*Npu* DnaE^N^) resulting in the plasmids pSE13 and pSE14, respectively. The 3’-fusion genes were cloned into the pBAD vector (Invitrogen). The vector backbone was obtained through a restriction digest of pAU07 with *Nde*I, *Bgl*II. The *Npu* DnaE^C^-CGGG-Trx-His_6_ insert resulted from a *Nde*I, *Bgl*II digest of pSE07 and the ligation gave pSE11. The other 3’-fusion gene *Npu* DnaE^C^-CFNK-Trx-His_6_, termed pSE12, was constructed through an *Nde*I, *Bgl*II digest of pSE10 and the subsequent ligation into the same vector fragment.

### Plasmids encoding the NRPS genes *grsB1* and *grsA*

The gene fragment encoding Gramicidin S Synthetase II C-A-PCP was PCR amplified from genomic DNA of *Bacillus brevis* ATCC 9999 with the primers (5’-ATAGCTAGCA GTACATTTAA AAAAGAACAT G) and (5’-ATAGCTAGCA GTACATTTAA AAAAGAACAT G). The *Nhe*I, *Bam*HI digested PCR product was cloned in an identical digested pET28a (Novagen, kanamycin resistance, *lacI*) vector. The vector fragment contained an additional 5’ StrepTag II (ST) to the *Nhe*I site and a 3’ His_6_-Tag to the *Bam*HI site. The resulting plasmid encoding for ST-GrsB1 C-A-PCP-His_6_ was termed pJZ56b. This plasmid was used to express and purify the wild-type GrsB1 protein. The S961 insertion into ST-GrsB1 C-A-PCP-His_6_ was cloned according to the QuikChange Site Directed Mutagenesis protocol (Stratagene), therefore, pJZ56b was used as the template together with the primers (5’-CTTCCGAATC TAGAGGGATC CATTGTGAAT ACAAACGC) and (5’-GCGTTTGTAT TCACAATGGA TCCCTCTAGA TTCGGAAG). pJZ75 is the plasmid encoding for this S961 insertion. The parental plasmid for approach **1)** (encoding ST-GrsB1 C-A-PCP-His_6_ in a p425-TDH shuttle vector) was termed pTS161 and resulted from a ligation of an *Nco*I, *Bam*HI digested pJZ56b insert into the identically cut pSE01 vector fragment (see above). The same insert was ligated with the *Nco*I, *Bam*HI cut pSE02 vector fragment (pET16b backbone, Novagen) to yield pTS220, the parental plasmid for approach **2)**. The gene encoding for the first module of the Gramicidin S biosynthesis Gramicidin S Synthetase I (GrsA A-PCP-E) was PCR amplified from genomic DNA of *Bacillus brevis* ATCC 9999 using the primers (5’-ATACCATGGC CAGTTGGAGC CACCCGCAGT TCGAAAAAGC TAGCATGTTA AACAGTTCTA AAAG) and (5’-ATAGGATCCC GTTAATGAAT CGGCC). The first primer introduced an *Nco*I restriction site and a 5’-StrepTag II sequence into the PCR product. The *Nco*I, *Bam*HI cut insert was ligated into an *Nco*I, *Bgl*II fragment of the pBAD vector (ampicillin resistance, *araC*, Invitrogen) which contained an additional 3’-His_6_-Tag. The expression plasmid encoding ST-GrsA A-PCP-E-His_6_ was termed pJZ50.

The GrsB1 DNA fragment amplified from the genomic DNA of *Bacillus brevis* ATCC 9999 showed minor differences to the previously published sequence . The DNA sequence used in this study results in amino acid substitutions at the positions 942-944 and 946-947 of the GrsB1 protein (Q_942_LPLTP instead of H_942_VRLHL). This region corresponds to the end of the small sub-domain of the A-domain.

### Integration of the *Ssp* DnaB split intein cassette into *grsB*1

The following primers were used in both approaches **1)** and **2)** to integrate the *Ssp* DnaB intein cassette into the non-ribosomal elongation module ST-GrsB1 C-A-PCP-His_6_. P9 (5’-CGGAAAAATA GACAGAAAAT CTCTTCCGAA TCTAGAGGGG TGCATCAGTG GAGATAGTTT), P10 (5’-TACACCAAAC GGAAAAATAG ACAGAAAATC TCTTCCGAAT GAATCTGGAT GCATCAGTGG AGATAG), P11 (5’-CATTTGTAGG TACTACATAT TTTGCGTTTG TATTCACAAT AGAGTTATGG ACAATGATGT), P12 (5’-CTTCCAGCTC ATTTGTAGGT ACTACATATT TTGCGTTTGT CTGTTCAATA GAGTTATGGA CAAT). First, the intein cassette was amplified from pCasDnaB2 using four different primer combinations as indicated in Table S3. Moreover the table shows the plasmids generated either via homologous recombination into *S. cerevisiae* in plasmid pTS161 or after RF-PCR cloning using pTS220.

**Table S3 Plasmids constructed in this study for identifying an active split intein insertion in GrsB1^S961^**.

| Combination | Primers | N-term. splice junction AAs | C-term. splice junction AAs | Plasmid name approach **1)** | Plasmid name approach **2)** |
| --- | --- | --- | --- | --- | --- |
| wt - no intein |  | LEG | IVN | pTS161 | pTS220 |
| GrsB1 ssp1 | P9, P11 | LEG | SIVN | - | pTS227 |
| GrsB1 ssp2 | P9, P12 | LEG | SIEQ | - | pTS228 |
| GrsB1 ssp3 | P10, P11 | ESG | SIVN | pTS179 | - |
| GrsB1 ssp4 | P10, P12 | ESG | SIEQ | pTS174 | - |

The combinations GrsB1 ssp3 & ssp4 showed reasonable test expressions yield with the helper plasmid pRSFara on the small-scale in *E. coli* BL21-Gold (DE3) cells (Stratagene). However, only small amount of protein could be purified from large scale expressions. Therefore, we recloned the 5’-coding region ST-GrsB1 C-A-ESG-Int^N^ and the two 3’-coding regions Int^C^-GrsB1-SIVN-PCP-His_6_ and Int^C^-GrsB1-SIEQ-PCP-His_6_ in three individual vectors. The insert coding for the complete N-terminal half was obtained through a restriction digest of pTS179 with the enzymes *Nco*I and *Not*I. The ligation with an *Nco*I, *Not*I cut pRSFDuet-1 (Novagene) vector fragment yielded pJZ76 (kanamycin resistance, *lacI*). The 3’-coding regions were PCR-amplified from template pTS179 and pTS174, respectively, with the primers (5’-ATACATATGG CTTCACCAGA AATAGAAAAG) and (5’-ATAAAGCTTA GTGATGGTGA TGGTG). The PCR products were digested with *Nde*I and *Hind*III and ligated with an identically cut pBAD vector (Invitrogen, Ampicillin resistance, *araC*). The resulting plasmids were pJZ78 (Int^C^-GrsB1-SIVN-PCP-His_6_) and pJZ79 (Int^C^-GrsB1-SIEQ-PCP-His_6_).

### Plasmid encoding for the uroporphyrinogen III methyltransferase CobA

The gene of the uroporphyrinogen III methyltransferase CobA from *propionibacterium freudenreichii* was PCR amplified from the plasmid pISA417 (obtained from the BCCM/LMBP plasmid collection) with the primers (5’ATAGCTAGCA CCACCACACT GTTGCCCGG) and (5’-TATGCGGCCG CTTAGTGATG GTGATGGTGA TGACTAGTGT GGTCGCTGGG CGCGCGATG). The first primer introduced a 5’-*Nhe*I restriction site, whereas the second primer introduced a 3’-His_6_-Tag sequence and an additional *Not*I restriction site to the PCR product. Then, the *Nhe*I and *Not*I cut PCR product was ligated with the identically cut vector fragment from pTS220 (pET16b backbone (Novagen), ampicillin resistance, *lacI*) which resulted in pSE21 (encoding for ST-CobA-His_6_). A FN (110-111) mutant of pSE21 was generated with the primers (5’-GAATGGCAGG CATGCTTCAA CGCCGGCATC CCGGTG) and (5’-CACCGGGATG CCGGCGTTGA AGCATGCCTG CCATTC) using the QuikChange Site Directed Mutagenesis protocol (Stratagene), resulting in pSE33 (ST-CobA_110FN_-His_6_).

### Integration of the *Npu* DnaE split intein cassette into *cobA*

The integration of the *Npu* DnaE intein cassette into the uroporphyrinogen III methyltransferase at cysteine 109 was achieved via RF-PCR (approach **2)**). The following primers were used: P13 (5’-CTCGTTCGTC TTCGGGCGTG GCGGCGAGGA ATGGCAGGCC TGTTTAAGCT ATGAAACGG), P14 (5’-CCGAGGAGAC TCCCGGGATC ACGCGCACCG GGATGCCGGC ATTGAAACAA TTAGAAGC), P15 (5’-AGACTCCCGG GATCACGCGC ACCGGGATGC CGGCCTCGGC ACAATTAGAA GCTATGAAGC), P16 (5’-GTTCACCGTC GTGTCGGGGC ATGTATCGCC CAGCGACGAG TACTGTTTAA GCTATGAAAC GG), P17 (5’-CCAGCGTGAG CCGGTCCTTG GCGAGTTGGC GCCATGGCAC GAAACAATTA GAAGCTATG), and P18 (5’-GGATCACCAG CGTGAGCCGG TCCTTGGCGA GTTGGCGCCA TTTATTGAAA CAATTAGAAG CTA). First, the intein cassette was amplified from pCasDnaE2 using two different primer combinations as indicated in Table S4. The four resulting plasmids (pSE25, pSE26, pSE35 and pSE36) are also described in Table S4.

**Table S4 Plasmids constructed in this study for identifying an active split intein insertion in CobA^C109^ and in CobA^S159C^.**

| Combination | Primers | N-term. splice junction AAs | C-term. splice junction AAs | Plasmid name approach **2)** |
| --- | --- | --- | --- | --- |
| wt - Pos 109 |  | WQA | CAEA | pSE21 |
| CobA npu1 | P13, P15 | WQA | CAEA | pSE26 |
| CobA npu2 | P13, P14 | WQA | CFNA | pSE25 |
| wt - Pos 159 |  | DER | SEVP | pSE21 |
| CobA npu3 | P16, P17 | DEY | CFVP | pSE35 |
| CobA npu4 | P16, P18 | DEY | CFNK | pSE36 |

For control reasons, we also cloned the individual split intein-fusion precursors of the C109 insertion position into separate vectors. The insert fragment for the N-terminal part (ST-CobA(109)^N^-Int^N^) was prepared through an *Nco*I, *Not*I restriction digest of pSE25 and ligated with an identically treated pET28a vector (Novagen). The resulting plasmid was termed pSE22. The two 3’-coding regions were transferred from the integration plasmids pSE25 and pSE26, respectively, via a *Bam*HI restriction digest into a *Bam*HI cut and dephosporylated pBAD vector (Invitrogen). pSE23 is encoding for Int^C^-CFNA-CobA(109)^C^-His_6_ and pSE24 codes for Int^C^-CAEA-CobA(109)^C^-His_6_.

## Yeast intein cassette integration

The generation of the integration plasmids in *S. cerevisiae* (approach **1)**) was done via homologous recombination of the amplified PTS cassettes into the target vector (procedure according to ). First an over day culture of the W303 strain harbouring the target vector was inoculated at an OD_600_ = 0.3, then the cells were grown in –Leu SD medium until they reached exponential growth phase (OD_600_ ~ 0.5). Second followed a lithium acetate mediated transformation of the yeast cells with an un-purified PCR mixture of the amplified intein cassettes combinations (see Tables S1-S3) and a subsequent selection on –Leu –His SD medium plates. After colony formation, selected colonies were used for overnight cultures in –Leu –His SD medium and a subsequent phenol-chloroform extraction was performed. A second yeast transformation with the DNA/RNA mixture was performed with selection on –Leu –His SD medium plates. The same protocol for a phenol-chloroform extraction was used on colonies that grew in this second selection step (required for a homogenous 2 micron plasmid solution). In the final step to isolate the integration plasmids 1 µl of the DNA/RNA mixture was used in a *E. coli* transformation of electro-competent Top10 (Invitrogen) or XL10-Gold® (Stratagene) cells.

## Restriction-free (RF) cloning - intein cassette integration

The insertion of the intein cassettes on the genetic level via approach **2**) was achieved following the previously published restriction-free cloning protocol . In short, standard PCR conditions were used to amplify the intein cassettes with the corresponding primers. For the second PCR 50 ng of template DNA and 300 ng of purified insert DNA were used (50 µl scale). The amplification was done with the PfuUltra II Fusion HS DNA Polymerase (Stratagene), 35 cycles: 95 °C for 30 s, 55 °C for 1 min and 68 °C for 30 s/kbp. Without further treatment, the PCR mixture was incubated with *Dpn*I at 37 °C overnight, and subsequently transformed into electro-competent Top10 (Invitrogen) or XL10-Gold® (Stratagene) cells. Positive clones were first screened via restriction digests and then further analysed via DNA sequencing. On average, 53.5 % of the picked colonies contained the desired integration plasmid (see Table S5).

**Table S5 Analysis of the generation of the model protein integration plasmids via RF-PCR.**

| **PTS cassette** | **Generated plasmid** | **Picked colonies** | **positive clones** | **Percentage (%)** |
| --- | --- | --- | --- | --- |
| *Npu* DnaE | pTS210 | 3 | 3 | 100 |
| *Npu* DnaE | pTS211 | 3 | 2 | 66.7 |
| *Npu* DnaE | pTS212 | 3 | 3 | 100 |
| *Npu* DnaE | pTS213 | 3 | 3 | 100 |
| *Ssp* DnaB | pTS216 | 3 | 1 | 33.3 |
| *Ssp* DnaB | pTS217 | 3 | 3 | 100 |
| *Ssp* DnaB | pTS218 | 11 | 1 | 9.1 |
| *Ssp* DnaB | pTS219 | 3 | 2 | 66.7 |
| *Ssp* DnaB | pTS227 | 6 | 1 | 16.6 |
| *Ssp* DnaB | pTS228 | 5 | 4 | 80 |
|  |  | **Total picked colonies** | **Total positive clones** | **Overall percentage** |
|  |  | 43 | 23 | 53.5 |

## Small-scale expression protocol

For both approaches **1)** and **2)**, selective induction can only be achieved with the help of the two regulatory proteins, LacI and AraC. Therefore, we first prepared heat competent *E. coli* BL21-Gold (DE3) cells (Stratagene) which contained the pRSFara plasmid (see above). These cells were then transformed with the integration plasmids obtained via approaches **1)** and **2)**. Selection occurred on ampicillin and kanamycin plates. The test expressions were done in 4 mL of LB media with 100 mg/L ampicillin, as well as with 50 mg/L kanamycin.

After growth overnight, an overday culture was inoculated at an OD_600_ = 0.2 and the cells were grown to an OD_600_ of 0.5 - 0.7 at 37 °C, then induced with 0.2 % Arabinose for 2 hours. After a washing step (first centrifugation at 5.000 rpm for one minute and resuspending the cells in fresh LB media with antibiotics and another centrifugation step) the cells were resuspended in LB media again containing both antibiotics. For the *Npu* DnaE integration plasmids the cells were incubated at 37 °C for 15 minutes, before the second induction with 1.2 mM IPTG for 4.5 hours was initiated. In case of the *Ssp* DnaB integration plasmids, the expression temperature for the N-terminal half was lowered to 25 °C. At the indicated time points samples were taken for SDS-PAGE and western blot analysis.

To determine the segmental isotopic labelling efficiency of an *in vivo* obtained splice product ST-gpD-Trx-His_6_ we used in gel-tryptic digest with subsequent MALDI-TOF MS analysis (for details of the procedure see below). This allows the screening of several different small-scale expression conditions to optimize the selective isotope incorporation in the splice product. Because, in the case of our model protein ST-gpD-Trx-His_6_, the N-terminal intein fusion protein and the splice product are very similar in size and it is difficult to separate them on an SDS-PAGE gel, we had to perform a Ni^2+^-NTA purification of the splice product before the tryptic digest. Therefore, we lysed a complete 8 mL expression culture using the EmulsiFlex-C3 high-pressure homogenizer (Avestin) and applied the supernatant to a standard Ni^2+^-NTA purification (Qiagen) according to the manufacturer’s recommendation. In cases where the splice product is clearly separated from the individual halves on an SDS-PAGE gel, the tryptic digest can directly be performed from the sample of the cell extract after the inductions.

## Protein purification of the individual Npu DnaE fusion proteins

For the expression of the individual *Npu* DnaE fusion proteins (ST-gpD-XXX-Int^N^ and Int^C^-XXX-Trx-His_6_), *E. coli* BL21Gold (DE3) cells (Stratagene) were transformed with the plasmids pSE11, pSE12, pSE13 and pSE14, respectively. Cells were grown in 300 mL of LB medium containing either 50 µg/mL kanamycin or 100 µg/mL ampicillin at 37 °C to an OD_600_ of ~ 0.6. Then the temperature was shifted to 28 °C and expression induced with 0.4 mM isopropyl-*β*-D-thiogalactopyranoside (IPTG) for the ST-containing Int^N^-constructs or with 0.02 % L-Arabinose for the His_6_-tag containing Int^C^-constructs. After 4-5 h cells were pelleted by centrifugation and stored at -80 °C. For protein purification of the Int^N^-constructs, the cell pellet was resuspended in wash buffer (100 mM Tris/HCl at pH 8.0, 150 mM NaCl, 1 mM EDTA). To purify the Int^C^-constructs under denaturing conditions, resuspension was done in buffer A-urea (50 mM Tris/HCl at pH 8.0, 300 mM NaCl, 8 M urea, 20 mM imidazole). Cells were lysed using the EmulsiFlex-C3 high-pressure homogenizer (Avestin), insoluble cell material was removed by centrifugation for 30 min at 35000 g, and proteins were purified on Strep-Tactin (Iba) and Ni^2+^-NTA (Qiagen) columns, respectively, according to the manufacturers’ recommendations. Elution fractions containing the purified proteins were pooled and dialyzed against splicing buffer (50 mM Tris/HCl at pH 7.0, 300 mM NaCl, 1 mM EDTA and 10 % (v/v) glycerol) and stored in aliquots at -80 °C. Protein concentrations were determined using the calculated absorbance at 280 nm.

## Western Blot

For all Western Blots shown in this paper the ECL Western Blotting Kit (GE Healthcare) was used after the manufacturer’s protocol. The dilutions for the primary antibodies were as followed: Anti-His (Qiagen) 1:2000, Anti-ST (IBA) 1:2000, Anti-MBP (Fermentas) 1: 10.000. In all cases a secondary HRP labelled anti-mouse antibody from GE Healthcare was used in a dilution of 1:4000.

## NMR spectroscopy

1 mM ST-gpD-Trx-His_6_, 0.5 mM ST-gpD-[^15^N]-Trx-His_6_ and 0.15 mM [^15^N]-ST-[^15^N]-gpD-Trx-His_6_ of purified, isotopically labelled 15N-labelled protein were prepared in 25 mM Tris/HCl, 50 mM NaCl, 2 mM DTT at pH 7.0 containing 5 % D_2_O and 0,15 mM DSS (4, 4-dimethyl-4-silapentane-1-sulfonate). NMR spectra were collected at a temperature of 293 K on 900 MHz (in case of ST-gpD-Trx-His_6_ and ST-gpD-[^15^N]-Trx-His_6_) and 700 MHz ([^15^N]-ST-[^15^N]-gpD-Trx-His_6_) Bruker Avance spectrometers equipped with cryogenic 5 mm z-axis gradient triple resonance probes. All spectra were processed with TopSpin (Bruker Biospin) and analysed with Sparky .

## Tryptic digest of SDS-PAGE bands

After separation by SDS-PAGE, the protein bands were cut out of the gel and incubated in 300 µL of washing solution (200 mM NH_4_HCO_3_, 50 % ACN) while shaking at 250 rpm for 30 min at 37 °C in an Eppendorf tube. The washing solution was removed and the gel band freeze-dried. Approximately 10 µL of digest solution (0.02 µg/µL modified Trypsin, 40 mM NH_4_HCO_3_, 10 % ACN, pH 8.1) was added to the gel band and incubated at RT for 45 min. After removal of a possible excess of digest solution the gel band was kept for 16-18 h at 37 °C. 15 µL of a diffusion solution (10 % ACN, 1 % TFA) was added and the sample was treated by ultrasonication for 45 min. The resulting solution was applied to MALDI-TOF-MS analysis on a Bruker autoflex II instrument.

## Determination of the isotopic labelling efficiency via MALDI-TOF MS analysis

The success of the *in vivo* small-scale isotope labelling reaction was determined by tryptic digest of the SDS-PAGE band corresponding to the splice product and subsequent MALDI-TOF MS analysis. In our experience, not all of the theoretically generated peptides by a tryptic digest can be found in the MS spectra. Depending on the ionisation probability some peptides are more readily detected than others. The detection of only one peptide fragment from each of the differently labelled protein parts is sufficient to determine the amount of isotope incorporation in each of the parts. If the two protein parts out of which the splice product is assembled differ highly in size, it might not be possible to detect a peptide fragment from the smaller part. However, we have not observed such a case with the proteins examined here. For comparison with the segmentally labelled splice products two reference samples were needed, one which was produced in completely unlabelled media and the other in which both inductions were conducted in ^15^N-minimal media. A mass shift depending on the number of nitrogen atoms in the peptide fragment is expected for the ^15^N-labelled sample compared with the unlabelled one (see Figure S2A, the fragment AS 15-41 in the N-terminal part (amino acid sequence ETFTHYQPQGNSDPAHTATAPGGLSAK) contains 35 nitrogen atoms and the mass of the monoisotopic peak shifts approx. 35 Daltons from m/z 2783.7 (black spectra) to m/z 2818.2 (red spectra); see Figure S2B for fragment AS 230-242 in the C-terminal part).


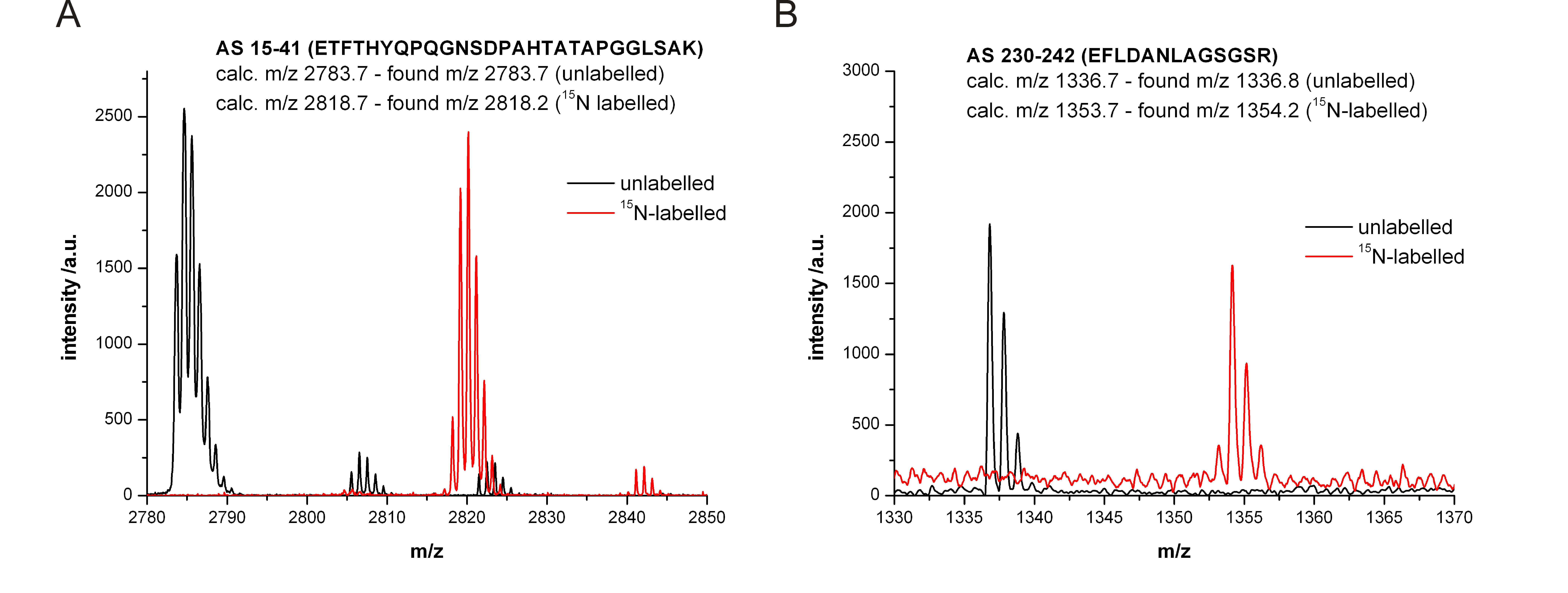


**Figure S2**.

**Labelling efficiency determination.** MALDI-TOF MS analysis of an N-terminal A) and a C-terminal B) peptide fragment after tryptic digest of the unlabelled (black) and completely (red) ^15^N-labelled model protein ST-gpD-Trx-His_6_ (AS denotes amino acid sequence).

In general, the percentage of isotope incorporation into one protein can best be calculated from the average molecular weight of a tryptic peptide (or of a peptide fraction, if two fractions are present as a result of undesired background labelling) and comparison with the average molecular weights of the control peptides of the unlabelled and the completely ^15^N labelled samples. The average molecular weight of one peptide MW_Peptide_ is just the sum of multiplying the intensities of the base-line separated individual isotope peaks (I_i_) with their corresponding masses (m_i_) divided by the total intensity of the peptide signal (see equation 1).

(1)

The percentage of isotope incorporation is then calculated through equation 2. MW_unlabelled_ and MW_labelled_ are the average molecular weights of the unlabelled and ^15^N-labelled control peptides, respectively.

(2)

If multiple test expressions are done in parallel the MS analysis can readily detect the conditions of undesired protein expression. The leaking of a promoter would result in two different populations of one peptide fragment in the splice product. We show this with two different small-scale expressions. The expression condition 1 was as follows: The cells are grown in LB-media up to an OD_600_ ~ 0.6 at 37 °C and the expression of the C-terminal half is induced upon the addition of 0.2 % Arabinose at 37 °C. After two hours, the media is exchanged to ^15^N minimal media (45 mM Na_2_HPO_4_, 25 mM KH_2_PO_4_, 8.5 mM NaCl, 0.1 mM CaCl_2_, 1 mM MgSO_4_, 0.03 mg/mL thiamine, 0.1 % ^15^NH_4_Cl, 0.2 % glucose, 22 nM FeCl_3_) and the expression of the N-terminal protein part is induced with 1.2 mM IPTG at 25 °C for 4 h. The SDS-PAGE band corresponding to the splice product was tryptic digested and analysed via MALDI-TOF MS (see also section small-scale expression of the SI). Comparison with the two reference spectra (unlabelled protein in black and completely labelled protein in red, see Figure S3A and B and a detailed analysis after equations 1 and 2 shows that an isotopic enrichment of 80 % has taken place in the N-terminal part and that the isotopic incorporation in the C-terminal part is below 1%. In expression condition 2, the second induction (with IPTG) is prolonged from 4 to 16 h compared with expression condition 1 (see Figure S3C and D, blue spectra). A tryptic digest of the splice product band in the SDS-PAGE gel, a MALDI-TOF MS analysis and the calculations according to equations 1 and 2 determine an isotopic enrichment of 97 % in the N-terminal part due to the longer incubation time in ^15^N-containing minimal media. For the fragment AS 230-242 in the C-terminal part two different populations can be found in the MALDI-TOF MS spectra. Therefore, the splice product contains molecules which are not labelled (similar pattern as the black control spectra) as well as molecules which are ^15^N-labelled (similar red control spectra) in the C-terminal part. The ratio of this undesired background labelling can be calculated from the signal intensities of the two fractions assuming that the ionisation probability is independent of the isotope incorporation and was determined with ~ 61 %. The longer incubation time in the ^15^N-containing minimal media presumably leads via lower glucose and higher cAMP levels to reduction of the catabolite repression and therefore to an undesired synthesis of the C-terminal intein fusion protein.


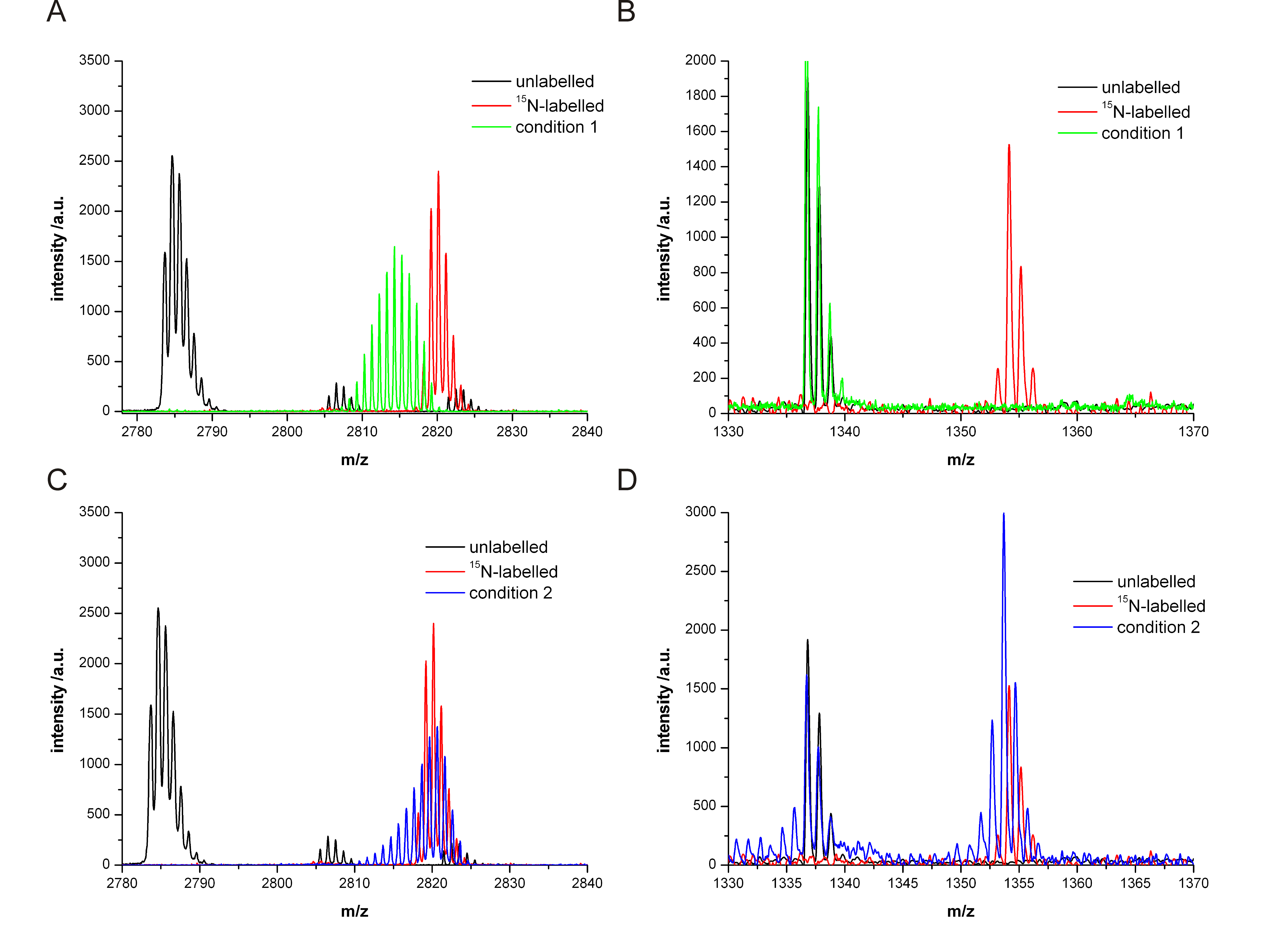


**Figure S3.**

**MALDI-TOF MS analysis of two different expression conditions for segmental isotopic labelling via *in vivo* PTS of the model protein ST-gpD-Trx-His_6_**. A) and B) MS spectra for condition 1 (green); C) and D) MS spectra for condition 2 (blue). All spectra are shown in comparison with a completely unlabelled (black) and a completely ^15^N-labelled (red) sample. A) and C) show an N-terminal fragment (amino acid sequence 15-41) and B) and D) show a C-terminal fragment (amino acid sequence 230-242) (for details on the expression conditions and MS analysis see text).

## Activity test for GrsB1 proteins - formation of D-Phe-L-Pro-Diketopiperazine

The enzymatic formation of the cyclic dipeptide D-Phe-L-Pro-diketopiperazine (DKP), and therefore the activity of the GrsB1 proteins, was tested according to previously published protocols . Apo proteins (ST-GrsA-His_6_, as well as ST-GrsB1-His_6_), dialyzed in assay buffer (50 mM HEPES pH 8.0, 100 mM NaCl, 1 mM EDTA, 2 mM DTT, 10 mM MgCl_2_) were converted into their holo form by incubation with coenzyme A (100 eq. of the apo protein) and recombinant Sfp (0.025 eq.) for 1 h at 37 °C. For DKP formation the holo GrsB1 proteins (1.8 µM) were mixed together with the holo GrsA protein (0.4 µM), L-Phe (1 mM) and L-Pro (1 mM) in a final volume of 100 µL. The reaction was started with the addition of ATP to a concentration of 2 mM and was performed at 37 °C. Negative controls were performed by omitting ATP, an amino acid, or one enzyme. The reaction was stopped after 2 h through the addition of 1 mL methanol and the proteins were precipitated overnight at ‑20 °C. After separation of the precipitated proteins by centrifugation (16000 g for 15 min) the supernatant was transferred to a fresh tube and removed under vacuum. The remainder was dissolved in 110 µL of 30 % (v/v) methanol and used for HPLC analysis. Separation of the reaction products was achieved by using an EC 150/4.6 Nucleodur C18 (Macherey-Nagel) column by applying an isocratic method at a flow rate of 0.6 mL min^-1^ with 30 % buffer B (0.045 formic acid/methanol, v/v; buffer A: 0.05 formic acid/water, v/v) as previously described . The products were identified by detection at 210 nm, collected and used for ESI-MS analysis (calc. mass of DKP 245.129 g/mol, meas. mass 245.129 g/mol).

## CobA activity test in E. coli

4 mL LB-media containing the corresponding antibiotics were inoculated from overnight *E. coli* cultures containing the CobA-plasmids (OD ~ 0.2). After one and a half hours at 37 °C the temperature was lowered to 25 °C and 1 mM δ-aminolevulinic acid (ALA) was added to each culture. All cultures were split into an induced and a non-induced sample. Additionally, the cultures with the integration plasmids (pSE25, 26, 35 and 36) were induced with 0.1 % Arabinose. 3.5 hours later, these cultures were shifted to 20 °C and induced for the second time with 0.05 mM IPTG. At the same time, the reference cultures (CobA WT and the 110FN mutant, N- and C-terminal halves) were induced. After 48 hours of incubation at 20 °C, the fluorescence intensity of the *E. coli* cultures was measured at an *Varian* Cary 100 Bio (Exc. 357 nm, Em. 605 nm). For that, a culture volume corresponding to an OD 1.0 was pelleted, washed once with 500 µL Dulbecco´s PBS without Ca^2+^ & Mg^2+^ and afterwards resuspended in 2 mL Dulbecco´s PBS.

# Extended Experimental Procedures

## The Mxe GyrA intein cassette

It was previously shown that the GyrA intein from *Mycobacterium xenopi* can artificially be split without the loss of splicing activity . The *Mxe* GyrA intein does not require a renaturation step for splicing activity and its nucleophile at the +1 position is a threonine.

After cloning the intein cassette in between the maltose binding protein (MBP) and the His_6_-Tag (see above for cloning details, for a schematic representation of the cassette see Figure S1, and the schematic PTS reaction can be found in Figure S4A), we transformed *E. coli* BL21 Gold (DE3) cells which already contained the helper plasmid pRSFara with the *Mxe* GyrA cassette plasmid. The western blot analysis of small-scale expressions is shown in Figure S4B). The selective induction of the generated protein parts is done via IPTG or with Arabinose. A successive induction of first the C-terminal protein part and after a media exchange of the N-terminal part leads to the formation of the splice product (MBP-His_6_), which can be detected by the two antibodies in a western blot analysis. To apply this intein cassette to proteins of interest it must be amplified including the FKBP as a 3’-extension of the 5’-fusion gene and a gpD fused to the 3’-intein fusion gene. These adaptations were made to increase the solubility of the fusion proteins as well as to retain the splice activity of the N-terminal protein half .

The results show that the *Mxe* GyrA intein is active in this particular extein context and that it can be used in the SPLICEFINDER technology to determine active insertion position in novel target proteins, either for the later use in the segmental isotopic enrichment of proteins, for NMR studies, or for the incorporation of fluorophores.


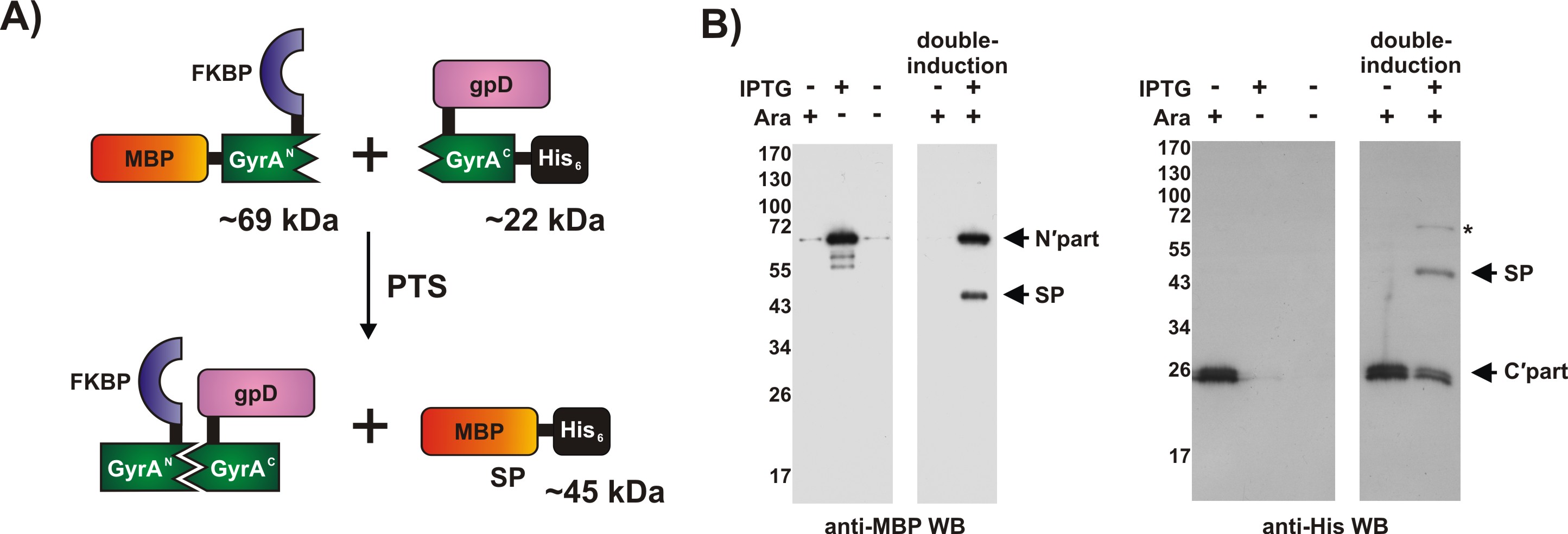


**Figure S4.**

**The Mxe GyrA intein cassette**. A) Schematic representation of the Mxe GyrA intein cassette mediated splice reaction B) Western blot analysis of small-scale expression of *E. coli* cells containing the intein cassette plasmid, as well as the helper plasmid pRSFara. The single inductions were done for 4 h at 37 °C (0.2 % arabinose or 0.4 mM IPTG). The dual inductions: 0.2 % arabinose for 2 h at 37 °C, then media exchange, and subsequent induction with 0.4 mM IPTG for 4 h, 25 °C. The theoretical molecular masses of the proteins are: SP = 44.7 kDa; N’Part = 69.3 kDa; C’Part = 22.2 kDa.

## Integration of the Ssp DnaB intein into the model protein gpD-Trx

As mentioned in the main text, we also integrated the *Ssp* DnaB intein cassette into the glycine linker of the model fusion gene gpD-Trx. Again, four different variations with flanking amino acids were prepared using approach **1)** and **2)** (see Table S1 for cloning details, Figure S1 for a schematic representation of the PTS cassette, and Figure S5A for a scheme of the PTS reaction). The resulting western blot analysis of the selectively induced small-scale expressions of the plasmids generated via approach **2),** all containing the helper plasmid, are shown in Figure S5 B) (the plasmids obtained via approach **1)** yielded identical results, however with reduced protein expression level, data not shown).


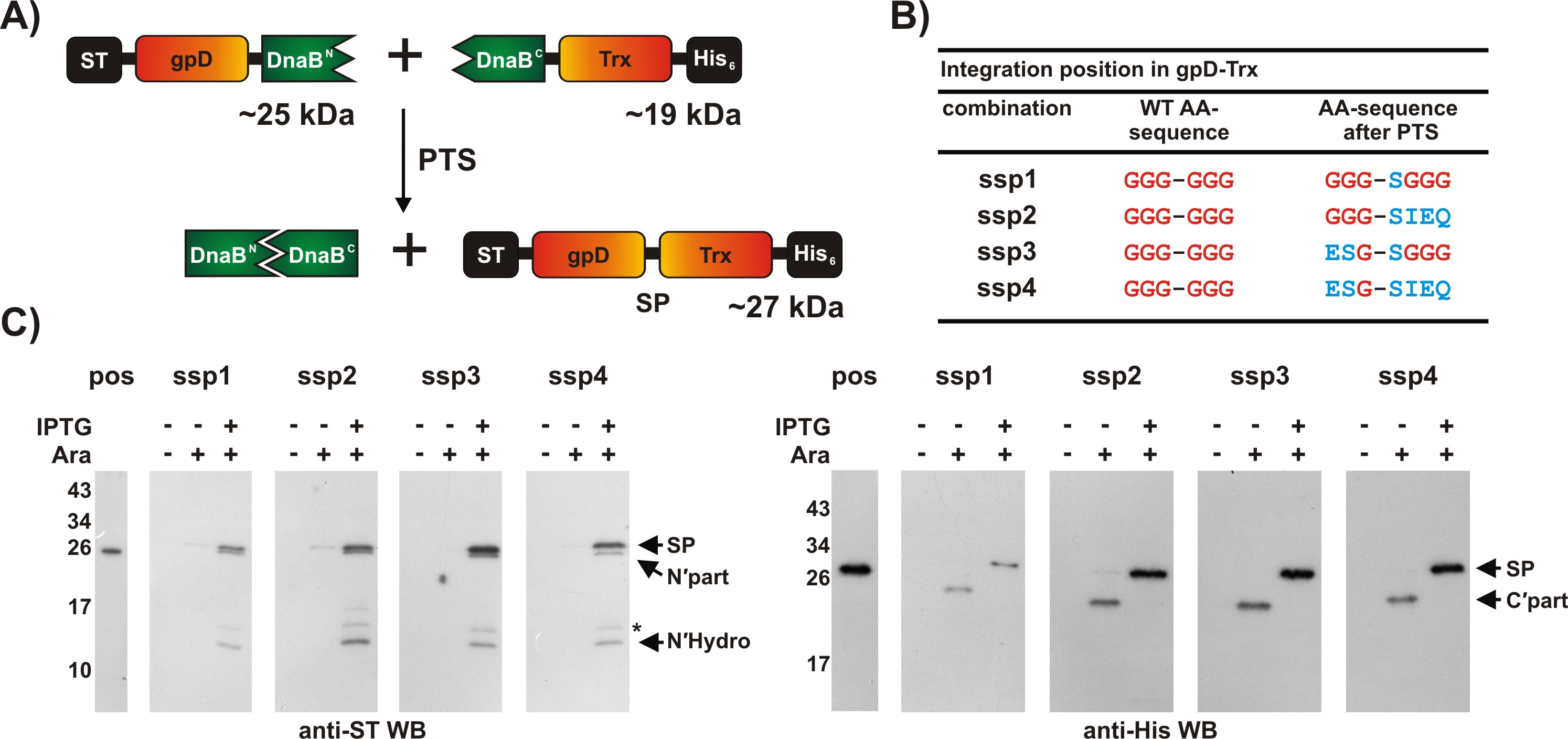


**Figure S5.**

**Integration of the *Ssp* DnaB intein cassette into gpD-Trx.** A) Schematic representation of the PTS reaction after the integration of the *Ssp* DnaB PTS cassette into ST-gpD-Trx-His_6_. B) Amino acid sequences at the splice junctions for the produced combinations in the linker region of ST-gpD-Trx-His_6_. Amino acids deviations after splicing from the original sequence (WT AA-sequence) are shown in blue. C) Western blot analysis of the four different flanking amino acids variations at the splice junction. All four combinations are splice active. The calculated molecular weights of the proteins are as follows: **SP** = 26.5-26.8 kDa; **N’Part** = 24.9-25.0 kDa; **C’Part** = 18.8-19.0 kDa; **N’Hydro** = 13.2 kDa. (pos = full length ST-gpD-Trx-His_6_).

The anti-His western blot in Figure S5C did show that all four variations resulted in an active intein, which was capable of linking the two extein halves together. Even the combination with three glycines at both splice junctions (ssp1), an inactive combination in the case of the *Npu* DnaE intein (Figure 2), showed splice product formation. Therefore, the *Ssp* DnaB intein seems to be more suitable for integration into glycine-rich linker regions than the *Npu* DnaE intein. Not surprisingly, when the amino acids at the N- and/or C-terminal splice junctions were adjusted to the naturally occurring extein sequences of this intein, an increased formation of splice product was observed. The anti-ST western blot detected, in addition to the splice product, a protein band corresponding to the size of the putative N-terminal hydrolysis product. This protein was observed independently of variable flanking amino acids, indicating that the N-terminal hydrolysis reaction might be an inherent property of the *Ssp* DnaB intein or this particular sequence context.

## Preparation of ^15^N-labelled gpD-Trx samples for NMR studies

**^15^N(ST-gpD-Trx-His_6_)**

For the complete ^15^N-labelling of ST-gpD-Trx-His_6_, *E. coli* BL21-Gold (DE3) (Stratagene) cells were transformed with the plasmid pSE02 (see cloning details above). The expression was done on a 300 mL scale. The cells were grown to an optical density of 0.6 at 37 °C in LB media, pelleted (centrifugation at 2500 g for 10 min) and washed once with 100 mL unlabelled minimal media. After another centrifugation (2500 g for 10 min), the cells were resuspended in 300 mL of ^15^N-M9 minimal media and incubated for 15 min with shaking at 28 °C before protein production was induced through the addition of 0.4 mM IPTG. After expression overnight, the cells were pelleted via centrifugation (7500 g for 15 min at 4 °C) and stored at -80 °C. The cell pellet was resuspended in buffer A (50 mM Tris/HCl at pH 8.0, 300 mM NaCl, 7.5 mM imidazole). Following a single purification step on a Ni^2+^-NTA column (Qiagen), according to the manufacturer’s recommendations, the pooled fractions were extensively dialyzed against NMR buffer (25 mM Tris/HCl at pH 7.0, 50 mM NaCl, 2 mM DTT). Concentrating the protein solution was achieved through VivaSpin columns MWCO 5000 (Sartorius) to a final concentration of approx. 500 µM.

**ST-gpD-^15^N(Trx-His_6_)**

The labelling of the C-terminal Trx-His_6_ fragment was achieved via *in vitro* splicing of the *Npu* DnaE intein with purified proteins. *E. coli* BL21-Gold (DE3) (Stratagene) cells were transformed with the plasmids pSE12 (coding for Int^C^-CFNK-Trx-His_6_) and pSE13 (coding for ST-gpD-GGG-Int^N^), respectively. Therefore, PTS with these recombinant protein yields the splice product with GGGCFNK at the splice junction. The intein fusion proteins were expressed and purified separately (see standard protein purification protocols above and lanes 1 and 2 in Figure S6). The C-terminal half was expressed in 600 mL ^15^N-minimal media. The proteins were both dialyzed against splice buffer (50 mM Tris, pH 7.0, 300 mM NaCl, 1 mM EDTA, 10 % (v/v) glycerol and 2 mM DTT). The splice reaction was performed at 4 °C overnight. The N-terminal part was used in an excess (1.5 eq., 20 µM) to ensure a complete conversion of the ^15^N-labelled C-terminal part (compare lanes 3 and 4 in Figure S6). Prior to a standard Ni^2+^-NTA affinity chromatography step (lane 5 in Figure S6), the mixture was dialyzed against buffer A (50 mM Tris, pH 8.0, 300 mM NaCl, 7.5 mM imidazole). To remove the co-eluted C-terminal cleavage by-product (also contains His_6_-affinity tag) from the desired splice product, the eluted fractions were pooled, dialyzed (100 mM Tris/HCl at pH 8.0, 150 mM NaCl, 1 mM EDTA) and applied to an additional Strep-Tactin purification (Iba) (lane 6 in Figure S6). Finally, the protein was extensively dialyzed against NMR buffer (25 mM Tris/HCl at pH 7.0, 50 mM NaCl, 2 mM DTT) and concentrated through VivaSpin columns MWCO 5000 (Sartorius) to a final concentration of approx. 200 µM.


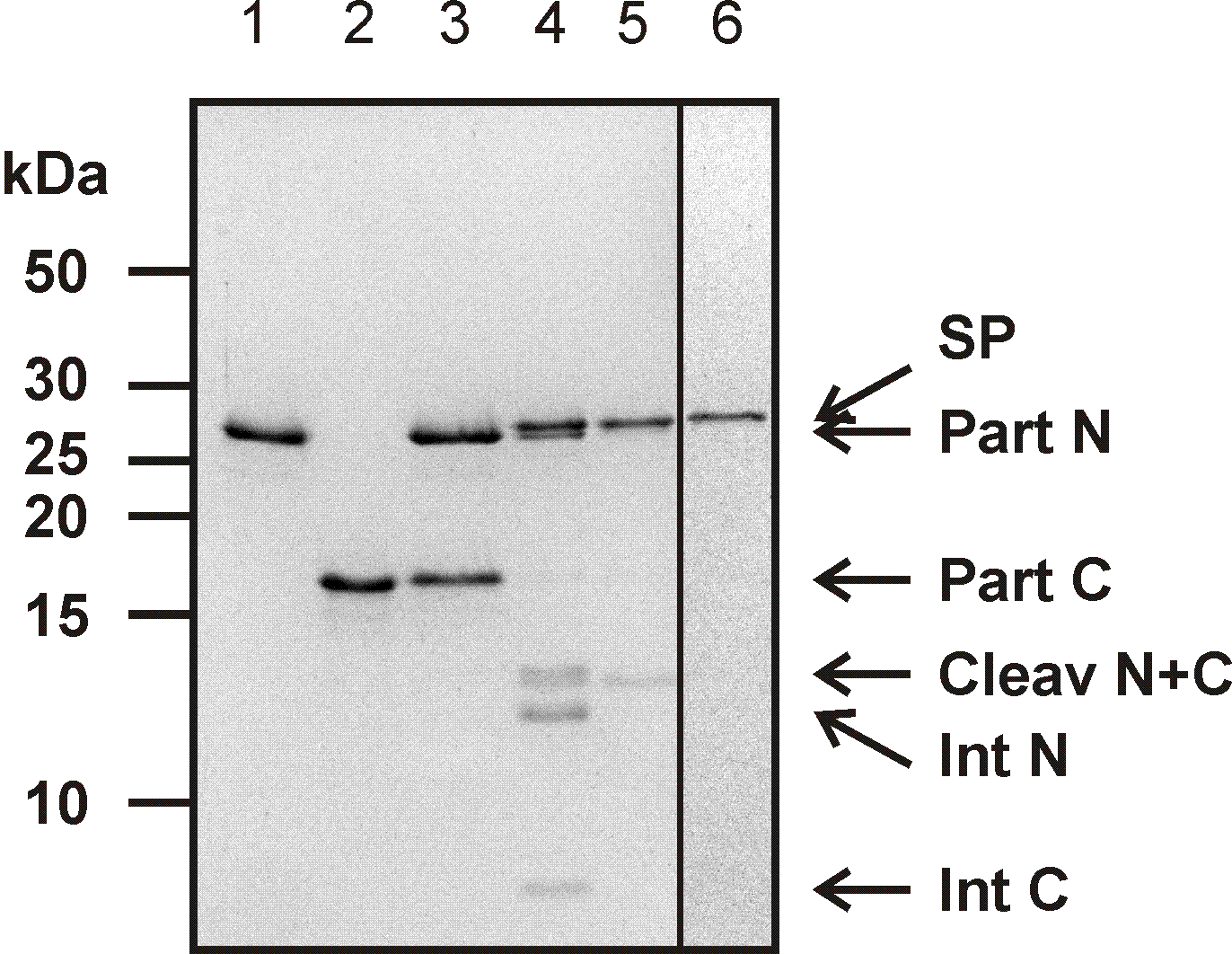


**Figure S6.**

***In vitro* PTS to obtain segmental labelled ST-gpD-^15^N(Trx-His_6_) with the *Npu* DnaE intein.** The SDS-PAGE gel of the PTS reaction and of the purification steps is shown in the Coomassie-staining. Lane 1: purified N-terminal part ST-gpD-Int^N^; lane 2: purified C-terminal part Int^C^-Trx-His_6_; lane 3: PTS-reaction at 0h; lane 4: PTS-reaction at 16 h; lane 5: combined elution fractions after Ni^2+^-NTA chromatography; lane 6: combined elution fractions after Strep-Tactin purification. The theoretical molecular masses of the proteins are as follows: **SP** = 26.7 kDa; **Part N** = 25.0 kDa; **Part C** = 17.6 kDa; **Cleav N** = 13.2 kDa; **Cleav C** = 13.5 kDa; **Int N** = 11.9 kDa; **Int C** = 4.1 kDa.

The labelling efficiency was determined through a tryptic digest of the splice product containing SDS-PAGE band and subsequent MALDI-TOF MS analysis (see Figure S7). Detailed analysis after equations 1 and 2 (see above) showed that the isotopic enrichment in the C-terminal part was 95 %, whereas the enrichment in the gpD part was below 1%.


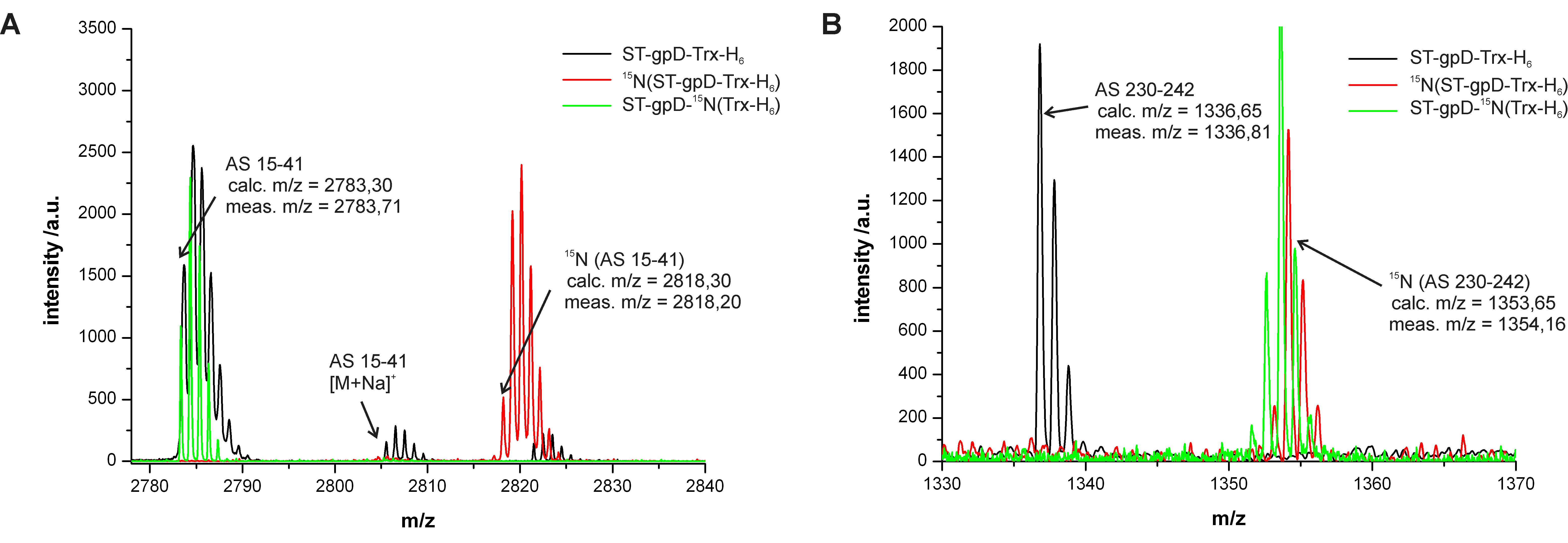


**Figure S7.**

**MALDI-TOF MS analysis of the segmental labelled gpD-^15^N(Trx) splice product.** A) Analysis of an N-terminal fragment AS 15-41, B) Analysis of a C-terminal fragment AS 230-242. Spectra of the unlabelled (black) and complete ^15^N labelled references (red) are shown in comparison with the segmental isotopically labelled gpD-^15^N(Trx) splice product (green) obtained through *in vitro* splicing with the *Npu* DnaE intein.

**^15^N(ST-gpD)-Trx-His_6_**

The isotopic enrichment of the N-terminal ST-gpD fragment was done via *in vivo* splicing with the *Ssp* DnaB intein. *E. coli* BL21-Gold (DE3) (Stratagene) cells carrying the helper plasmid (pRSFara) were transformed with the plasmid pTS218 (see above). After occurrence of protein splicing the protein does have the amino acid sequence: ESGSGGG at the splice junction. The cells were grown in 300 mL of LB-media to an OD_600_ of ~ 0.6 at 37 °C. Then, the expression of the C-terminal part Int^C^-Trx-His_6_ was induced through the addition of 0.2 % arabinose for 2 h still at 37 °C. The cells were pelleted (centrifugation at 2500 g for 10 min) and washed once with 100 mL unlabeled minimal media to remove residual arabinose. After another centrifugation step at 2500 g for 10 min the pellet was resuspended in 300 mL ^15^N-containing minimal media and incubated at 25 °C with shaking at 250 rpm for 15 min. The N-terminal protein part ST-gpD-Int^N^ was induced upon the addition of 1.2 mM IPTG and the whole culture was incubated for further 4 h at 25 °C. The purification of the splice product was done through a single His_6_-Tag purification step (standard protocol, see above), because the C-terminal part which also contains a His_6_-Tag was completely consumed in the PTS reaction. We observed only traces of C-terminal cleavage products (data not shown). The pooled protein fractions were again extensively dialyzed against NMR buffer (25 mM Tris/HCl at pH 7.0, 50 mM NaCl, 2 mM DTT) and concentrated with VivaSpin columns MWCO 5000 (Sartorius) to a final concentration of approximately 500 µM. The labelling efficiency was determined through a tryptic digest of the splice product containing SDS-PAGE band and subsequent MALDI-TOF MS analysis (see Figure S8). Detailed analysis after equations 1 and 2 (see above) showed that the isotopic enrichment in the N-terminal part was 83 %, whereas the enrichment in the Trx part was below 1 %.


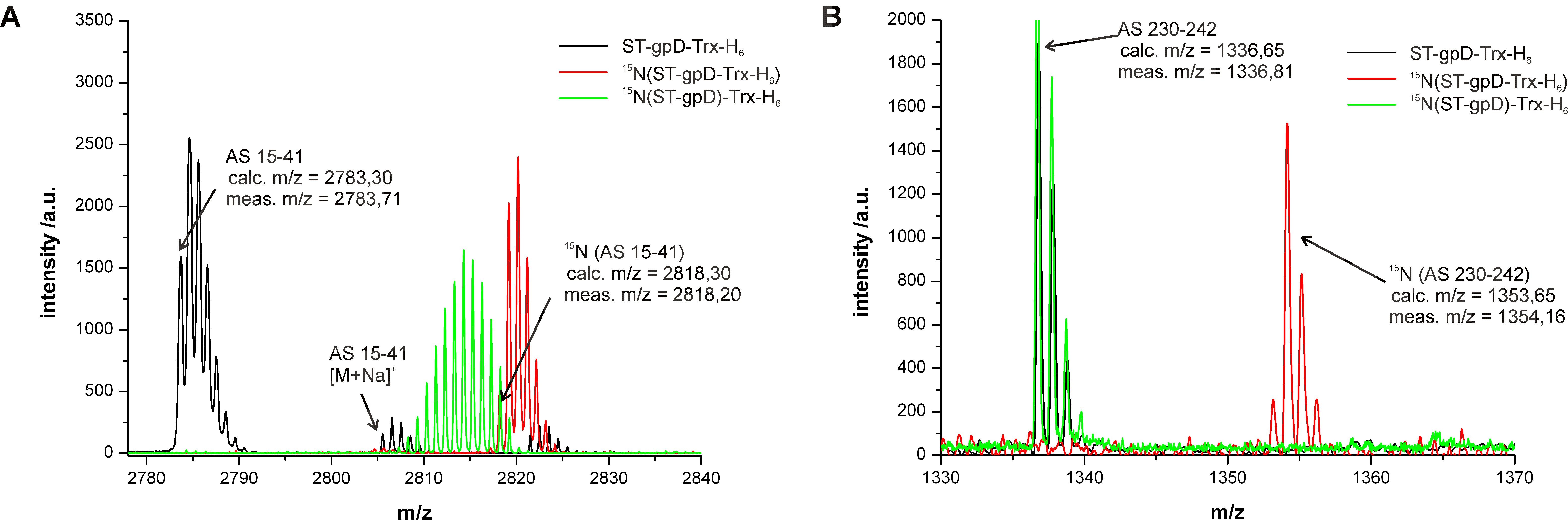


**Figure S8.**

**MALDI-TOF MS analysis of the segmental labelled ^15^N(gpD)-Trx splice product.** A) Analysis of an N-terminal fragment AS 15-41, B) Analysis of a C-terminal fragment AS 230-242. Spectra of the unlabelled (black) and complete ^15^N labelled references (red) are shown in comparison with the segmental isotopically labelled ^15^N(gpD)-Trx splice product (green) obtained through *in vivo* splicing with the *Ssp* DnaB intein.

## Integration of the Ssp DnaB intein into the complete non-ribosomal peptide synthetase module GrsB1

As model protein for SPLICEFINDER we chose the second module of the Gramicidin S biosynthesis pathway (see Figure S9, GrsB1).


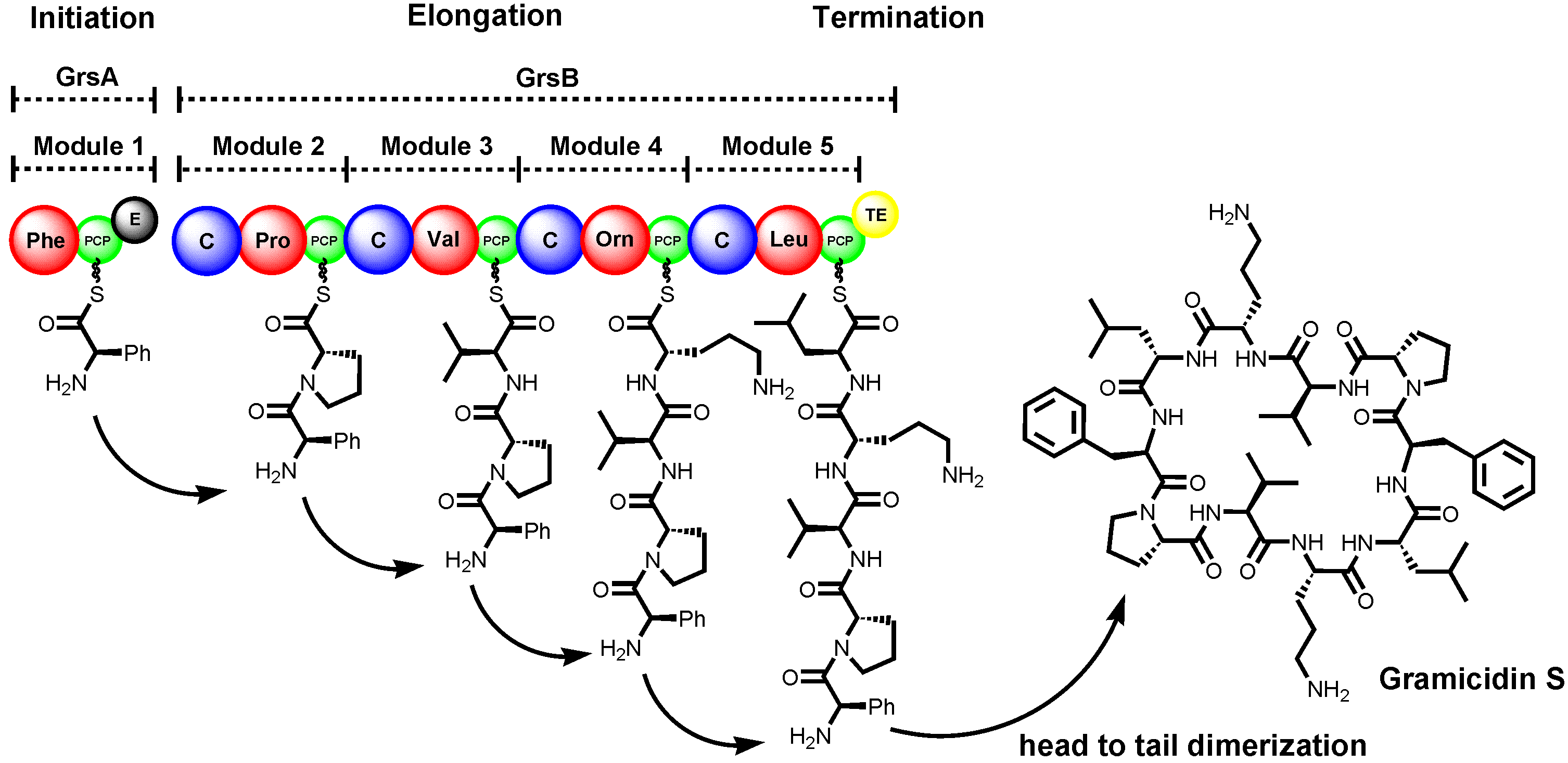


**Figure S9.**

**Biosynthesis of the antibiotic Gramicidin S.** Two NRPS multi-domain proteins are responsible for the formation of Gramicidin S, namely Gramicidin S Synthetase I (GrsA) and Gramicidin S Synthetase II (GrsB). In the first round, each module (one in GrsA and four in GrsB) incorporates one amino acid into the growing peptide chain tethered as thioesters on the phosphopantetheinyl group of the peptidyl carrier protein (PCP) domains. This leads to a pentapeptide (*D*-Phe-Pro-Val-Orn-Leu) which is transferred onto the thioesterase (TE) domain. After a second round of pentapeptide formation, the two peptides are dimerized and cyclized in a head to tail manner to yield Gramicidin S.

As the intein insertion position we chose the linker region between the adenylation (A) and the PCP domain (amino acid sequence in the linker region LEG_961_IVN - see Figure 4B). We tested four different variations of flanking amino acids at that particular position: splice product of combination GrsB1 ssp1 would contain only a serine insertion compared to the wild type sequence. In combination GrsB1 spp4 the flanking amino acids on both sites are optimised for the naturally occurring *Ssp* DnaB intein context.

The four different combinations (pTS227, pTS228, pTS179 and pTS174, see Table S3) were analysed for their splice activity in small-scale expressions. Again, a selective induction was achieved through the helper plasmid pRSFara. The results of the western blot analysis are shown in Figure S10.


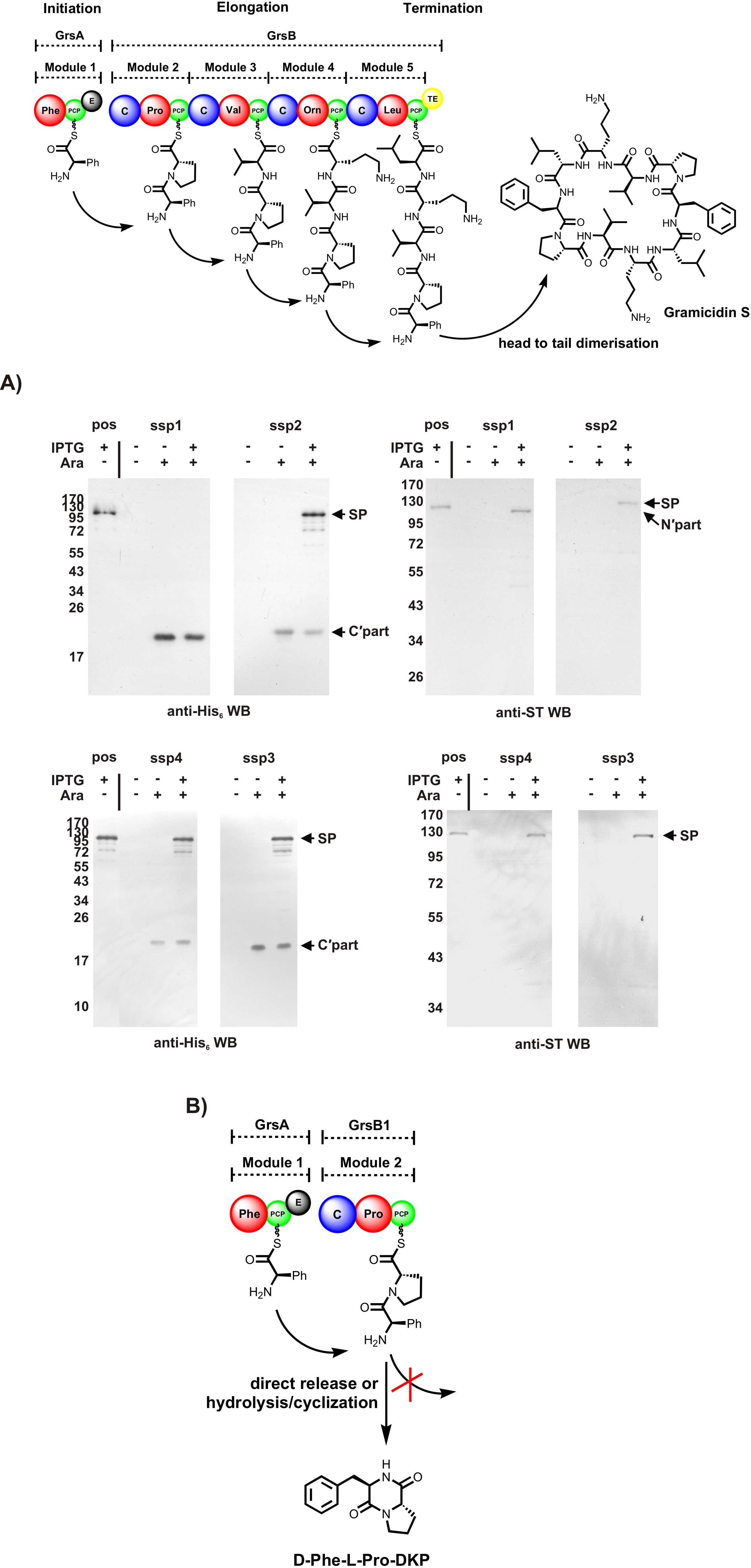


**Figure S10.**

**Integration of the *Ssp* DnaB intein cassette into ST-GrsB1-His_6_.** Western blot analysis of the four of flanking amino acids combinations at the splice junction (GrsB1 ssp1-4, Figure 4B). Arabinose induction lasted for 2 h; with an additional 3 h for the IPTG double-induction. Purified WT ST-GrsB1-His_6_ protein is indicated as pos. The theoretical molecular masses of the proteins are as follows: **SP** = 124.2 kDa; **Part N** = 124.9 kDa; **Part C** = 16.7 kDa.

In only one out of these four cases (GrsB1 ssp1, see Figure 4B), the one in which the splice product would only contain a single serine insertion compared with the wild type linker sequence of GrsB1, it was not possible to detect the splice product. The presence of bands corresponding to the C-terminal half in the anti His WB, as well as the N-terminal half in the anti-ST WB (running slightly below the splice product) indicated that this combination is indeed not splice active. Adjusting two amino acids at the +3 and +4 positions to the native extein residues of the DnaB intein restored intein activity (GrsB1 ssp2). The same was observed for the -3 and -2 positions in the N-terminal part (GrsB1 ssp3) as well as for a combination of the double adjustment (GrsB1 ssp4).

The large scale preparative formation of the GrsB1 splice products was achieved via *in vivo* PTS with subsequent protein induction. To obtain the splice product with the combination LEGSIEQ at the splice junction we used the available plasmid prepared via approach **2)** (pTS228). The two other splice active combinations (pTS174 and pTS179, approach **1**) showed only little protein expression on the larger scale, therefore we recloned the individual N- and C-terminal halves in bi-inducible plasmids (see cloning details above). The expression and splice product formation were done as follows: *E. coli* BL21-Gold (DE3) (Stratagene) cells were co-transformed with the combination pJZ76/pJZ78 or pJZ76/79 (see cloning part). Additionally, the same *E. coli* cells carrying the helper plasmid (pRSFara) were transformed with the plasmid pTS228 (see above). The cells were grown in 300 mL LB-media to an OD_600_ of ~ 0.6 at 37 °C. Then, the expression of the C-terminal part Int^C^-PCP-His_6_ was induced through the addition of 0.2 % arabinose for 1.5 h still at 37 °C. The temperature was lowered to 25 °C and the N-terminal protein part ST-C-A-Int^N^ was induced upon the addition of 1.2 mM IPTG and the whole culture was incubated overnight at 25 °C (for representative expression levels see Figure 4 C).

The purification of the splice product was achieved via a Ni^2+^-NTA purification step (standard protocol). The combined elution fractions contained additionally to the GrsB1 splice product impurities at a molecular weight of 20 kDa and below (e.g. the not completely consumed C-terminal part). Therefore, we dialyzed the elution fractions against assay buffer (50 mM HEPES pH 8.0, 100 mM NaCl, 1 mM EDTA, 2 mM DTT and 10 mM MgCl_2_) and performed an additional gel filtration purification on a Superdex 200 10/300 GL column (GE Healthcare).

To test whether a spliced GrsB1 protein is still capable of performing peptide synthesis we used the previously described D-Phe-L-Pro-diketopiperazine assay (see Figure S11) . Here, the incubation of the first two modules of the Gramicidin S biosynthesis pathway together with the appropriate substrates yields a dipeptide tethered as thioester onto the PCP domain of GrsB1 (see above for experimental details). Spontaneous, uncatalyzed cyclization results in the release of D-Phe-L-Pro-DKP which can be detected via HPLC analysis.


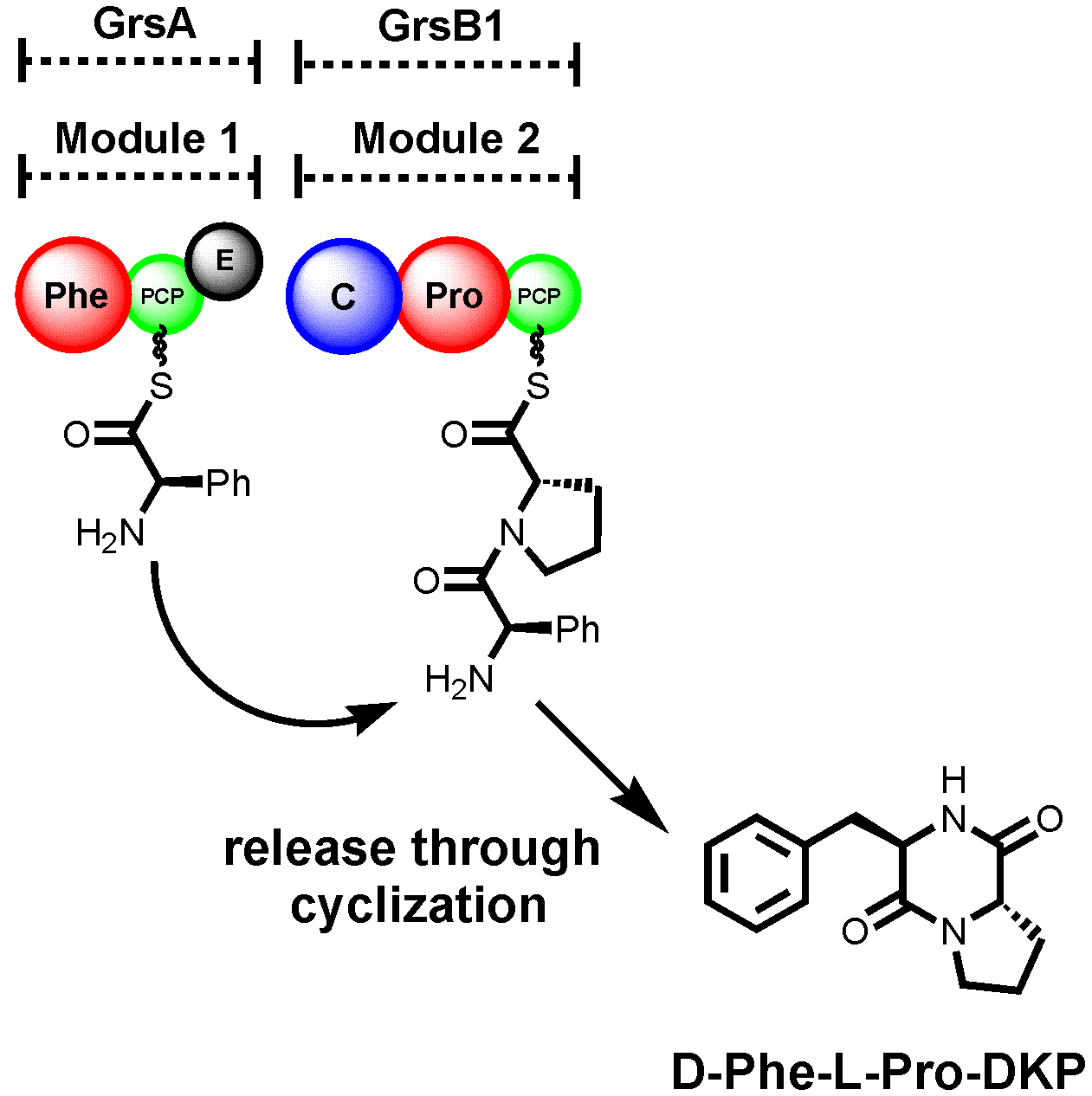


**Figure S11.**

**Scheme of the formation of D-Phe-L-Pro-DKP with the first two modules of Gramicidin S biosynthesis, GrsA and GrsB1.**

We first had to evaluate if a serine insertion after G_961_ in the linker region between the A and the PCP domain of GrsB1 would result in a loss of activity in the DKP assay. We therefore constructed the plasmid through site-directed mutagenesis (see cloning details), expressed and purified the protein ST-GrsB1_S962(insertion)_-His_6_ recombinantly and subjected it to the DKP assay. The formation of the cyclic dipeptide indicated that the serine insertion in the linker region is tolerated (data not shown). Interestingly, all three GrsB1 proteins obtained through PTS with the *Ssp* DnaB intein were able to catalyse the formation of DKP (see Figure 4C). This suggests that the linker region between the A- and the PCP-domains in NRPS tolerates amino acid insertions and substitutions.

## Integration of the Npu DnaE intein into the uroporphyrinogen III methyltransferase CobA

The uroporphyrinogen III methyltransferase (CobA) of *Propionibacterium freudenreichii* is involved in the tetrapyrrole biosynthesis and catalyzes the conversion of uro(porphyrino)gen III to precorrin-2 . S-Adenosylmethionin (SAM) acts as the methyl group donor for the two methylation reactions at the tetrapyrrol ring (see Figure S12 A for the reaction scheme).

For the integration into CobA we choose the *Npu* DnaE cassette. Because the *Npu* DnaE intein possesses a cysteine residue at the +1 position we selected residue 109, the only native cysteine, as our first integration region (Position 1, Figure S12 B). For a second position of integration we looked for an unconserved region of the protein, because we assumed a higher probability of the protein to tolerate amino acid substitutions. After aligning CobA of *P. freudenreichii* and 12 homologue proteins, we selected the serine residue 159 to integrate the intein cassette and thus converting it into a cysteine (Position 2, Figure S12 B). Corresponding positions 109 & 159 are highlighted in the structure of the *Thermus thermophiles* uroporphyrin III methyltransferase (Figure S12 C), arising from the sequence alignment with CobA of *P. freudenreichii*.


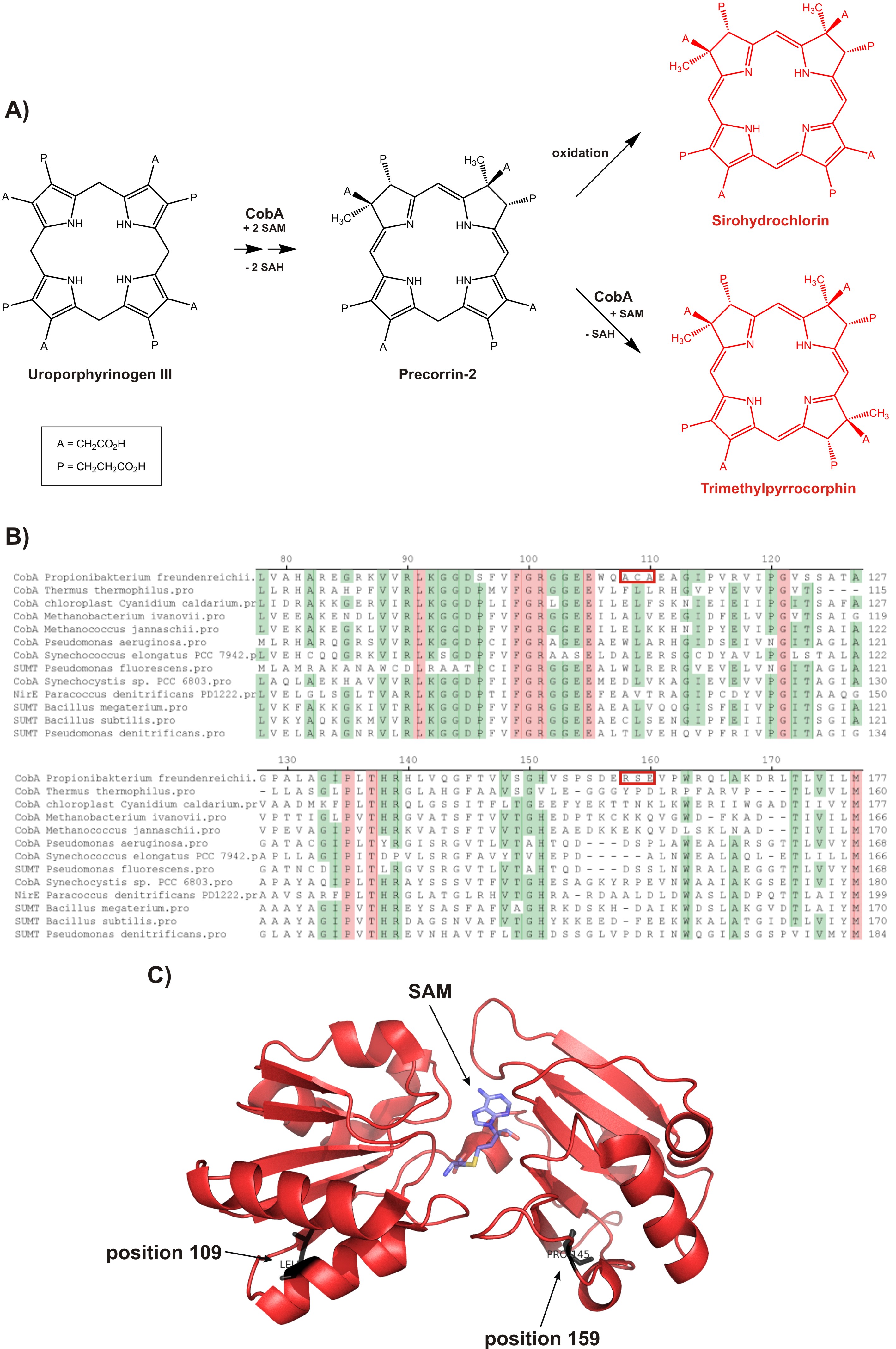


**Figure S12**.

**The uroporphyrinogen III methyltransferase (CobA).** A) Reaction pathway of the uroporphyrinogen III methyltransferase (CobA). CobA catalyzes the conversion of uroporphyrinogen III to precorrin-2 through the consumption of two molecules of SAM. An overproduction of CobA results in an accumulation of the red fluorescent compounds sirohydrochlorin and trimethylpyrrocorphin. (A = acetate, P = propionate). B) Sequence alignment of uroporphyrinogen III methyltransferases from diverse organisms. Complete invariant residues are coloured in red, conserved residues with at least 8 out of 13 are shown in green; the two insertion positions of the *Npu* DnaE intein are indicated. C) Crystal structure of the uroporphyrin III methyltransferase from *Thermus thermophilus* (pdb-code 1V9A ). After sequence alignment with the uroporphyrinogen III methyltransferase of *P. freudenreichii*, the equivalent insertion positions are indicated.

The integration of the split intein cassette on the genetic level was done via approach **2)** (see cloning details, and Table S4) and we performed small-scale expressions to determine the splicing ability of the corresponding intein halves of the integration at residue 109.

Despite the fact that the splice product as well as the affinity tagged CobA proteins were expressed at 37 °C (data not shown), no red fluorescence could be observed in the pelleted cells indicating a loss of activity. Therefore the expression temperature was reduced to 20 °C and after 48 hours samples were taken to investigate the PTS reaction (Figure 5C) and to determine the fluorescence intensity of the *E. coli* cells (Figure 5 D).

All combinations for integration sites 109 and 159 showed formation of splice product, nearly depleting the arabinose induced expression of the C-terminal intein fusion protein (Figure 5C). Western Blot analysis detected that the amount of splice product was only decreased for the combination CobA npu1, confirming the results obtained at 37 °C (data not shown). The measurements of the fluorescence intensity indicated that all via PTS assembled ST-CobA-His_6_ proteins were still active (Figure 5D).

To further determine if the loss of activity of the CobA proteins at higher temperatures originates from the insolubility of the proteins, we expressed full length ST-CobA_110FN_-His_6_ and its corresponding PTS cassette plasmid, both resulting in the same protein. Expression was performed at 37 and 20 °C and the proteins were purified via the His affinity tag (Figure S13**)**.


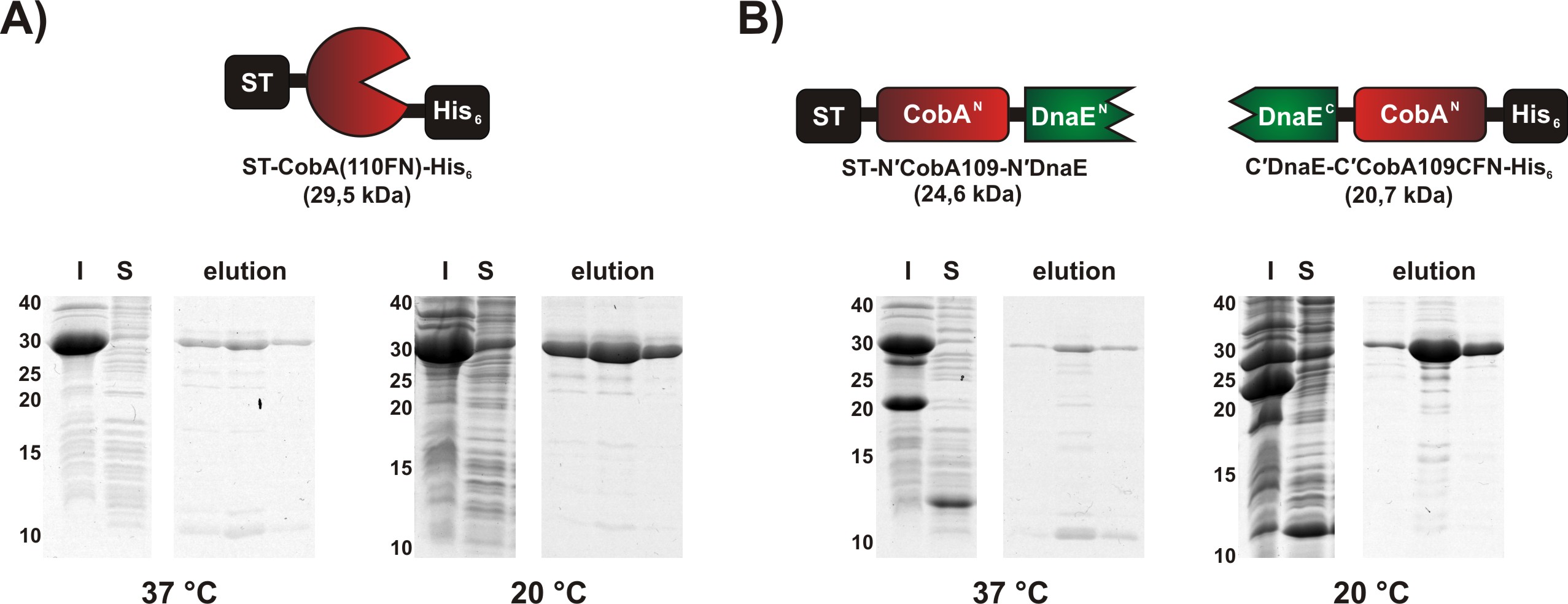


**Figure S13.**

**SDS-PAGE analysis of the expression and subsequent purification of full length and spliced ST-CobA-His_6_ proteins at different temperatures.** A) The mutant protein ST-CobA110FN-His_6_. B) CobA splice product formation (identically to ST-CobA_110FN_-His_6_) after co-induction of both fusion genes. (I = insoluble fraction after cell lysis; S = soluble fraction after cell lysis; elution = the first three elution fractions of the Ni^2+^-NTA affinity chromatography)

In both cases the soluble fraction as well as the amount of purified ST-CobA_110FN_-His_6_ protein increased by at least a factor of 10 at 20 °C in comparison to 37 °C. Interestingly at both temperatures the C-terminal intein fusion protein (C’DnaE-C’CobA-His_6_) was neither soluble nor was it possible to purify it.

# Acknowledgements

We thank Michael Albers for cloning of plasmids pJZ75, pJZ76, pJZ77, pJZ78 and pJZ79 and help with the GrsB1 project. The plasmid p425-TDH was a kind gift from Prof. Lill (Institut für Zytobiologie und Zytopathologie, Philipps-Universität Marburg). We also thank Dr. Christina Ludwig for providing plasmid pCL20, Annika Urbanek for pAU04, pAU06, pAU07 and pAU08, Annika Inkemann for plasmid pAI13 and Dania Kendziora for pDK01.

# Literature

1. Wach A, Brachat A, Alberti-Segui C, Rebischung C, Philippsen P (1997) Heterologous HIS3 marker and GFP reporter modules for PCR-targeting in Saccharomyces cerevisiae. Yeast 13: 1065-1075.

2. Sonntag T, Mootz HD (2011) An intein-cassette integration approach used for the generation of a split TEV protease activated by conditional protein splicing. Mol Biosyst 7: 2031-2039.

3. Zettler J, Schutz V, Mootz HD (2009) The naturally split Npu DnaE intein exhibits an extraordinarily high rate in the protein trans-splicing reaction. FEBS Lett 583: 909-914.

4. Brachmann CB, Davies A, Cost GJ, Caputo E, Li JC, et al. (1998) Designer deletion strains derived from Saccharomyces cerevisiae S288C: a useful set of strains and plasmids for PCR-mediated gene disruption and other applications. Yeast 14: 115-132.

5. van den Ent F, Lowe J (2006) RF cloning: a restriction-free method for inserting target genes into plasmids. J Biochem Biophys Methods 67: 67-74.

6. Ludwig C, Pfeiff M, Linne U, Mootz HD (2006) Ligation of a synthetic peptide to the N terminus of a recombinant protein using semisynthetic protein trans-splicing. Angew Chem Int Ed Engl 45: 5218-5221.

7. Turgay K, Krause M, Marahiel MA (1992) 4 Homologous Domains in the Primary Structure of Grsb Are Related to Domains in a Superfamily of Adenylate-Forming Enzymes. Molecular Microbiology 6: 529-546.

8. Sattler I, Roessner CA, Stolowich NJ, Hardin SH, Harris-Haller LW, et al. (1995) Cloning, sequencing, and expression of the uroporphyrinogen III methyltransferase cobA gene of Propionibacterium freudenreichii (shermanii). J Bacteriol 177: 1564-1569.

9. Goddard TD, Kneller DG SPARKY 3. University of California, San Francisco.

10. Miyada CG, Stoltzfus L, Wilcox G (1984) Regulation of the araC gene of Escherichia coli: catabolite repression, autoregulation, and effect on araBAD expression. Proc Natl Acad Sci U S A 81: 4120-4124.

11. Guzman LM, Belin D, Carson MJ, Beckwith J (1995) Tight regulation, modulation, and high-level expression by vectors containing the arabinose PBAD promoter. J Bacteriol 177: 4121-4130.

12. Stachelhaus T, Mootz HD, Bergendahl V, Marahiel MA (1998) Peptide bond formation in nonribosomal peptide biosynthesis - Catalytic role of the condensation domain. Journal of Biological Chemistry 273: 22773-22781.

13. Linne U, Stein DB, Mootz HD, Marahiel MA (2003) Systematic and quantitative analysis of protein-protein recognition between nonribosomal peptide synthetases investigated in the tyrocidine biosynthetic template. Biochemistry 42: 5114-5124.

14. Kurpiers T, Mootz HD (2008) Site-specific chemical modification of proteins with a prelabelled cysteine tag using the artificially split Mxe GyrA intein. Chembiochem 9: 2317-2325.

15. Reuter K, Mofid MR, Marahiel MA, Ficner R (1999) Crystal structure of the surfactin synthetase-activating enzyme Sfp: a prototype of the 4 '-phosphopantetheinyl transferase superfamily. Embo Journal 18: 6823-6831.

16. Warren MJ, Roessner CA, Santander PJ, Scott AI (1990) The Escherichia coli cysG gene encodes S-adenosylmethionine-dependent uroporphyrinogen III methylase. Biochem J 265: 725-729.

17. Vevodova J, Graham RM, Raux E, Schubert HL, Roper DI, et al. (2004) Structure/function studies on a S-adenosyl-L-methionine-dependent uroporphyrinogen III C methyltransferase (SUMT), a key regulatory enzyme of tetrapyrrole biosynthesis. J Mol Biol 344: 419-433.

18. Rehse PH, Kitao T, Tahirov TH (2005) Structure of a closed-form uroporphyrinogen-III C-methyltransferase from Thermus thermophilus. Acta Crystallogr D Biol Crystallogr 61: 913-919.
